# Interstrand Aminoacyl Transfer in a tRNA Acceptor Stem-Overhang Mimic

**DOI:** 10.1021/jacs.1c05746

**Published:** 2021-07-20

**Authors:** Long-Fei Wu, Meng Su, Ziwei Liu, Samuel J. Bjork, John D. Sutherland

**Affiliations:** MRC Laboratory of Molecular Biology, Francis Crick Avenue, Cambridge Biomedical Campus, Cambridge CB2 0QH, United Kingdom

## Abstract

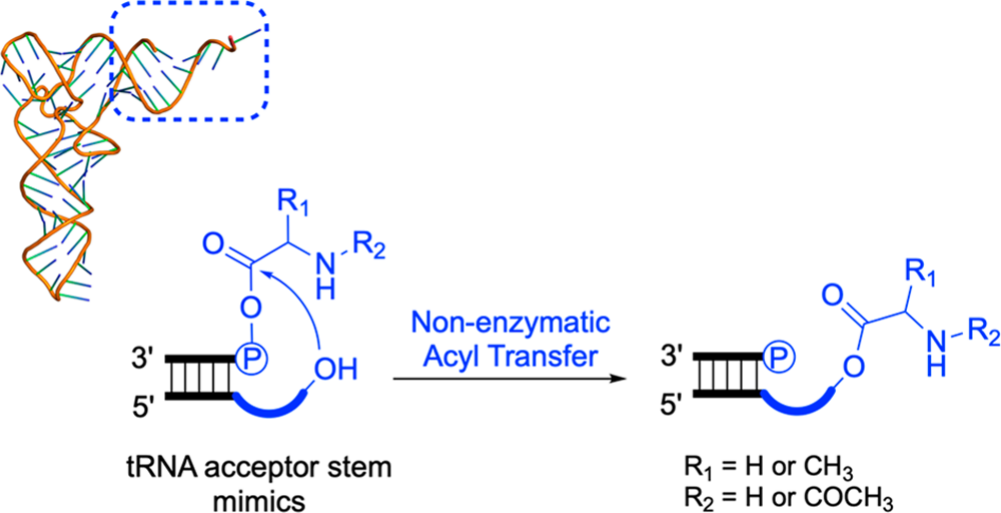

Protein-catalyzed aminoacylation of the 3′-overhang of tRNA by an aminoacyl-adenylate could not have taken place prior to the advent of genetically coded peptide synthesis, and yet the latter process has an absolute requirement for aminoacyl-tRNA. There must therefore have been an earlier nonprotein-catalyzed means of generating aminoacyl-tRNA. Here, we demonstrate efficient interstrand aminoacyl transfer from an aminoacyl phosphate mixed anhydride at the 5′-terminus of a tRNA acceptor stem mimic to the 2′,3′-diol terminus of a short 3′-overhang. With certain five-base 3′-overhangs, the transfer of an alanyl residue is highly stereoselective with the l-enantiomer being favored to the extent of ∼10:1 over the d-enantiomer and is much more efficient than the transfer of a glycyl residue. *N*-Acyl-aminoacyl residues are similarly transferred from a mixed anhydride with the 5′-phosphate to the 2′,3′-diol but with a different dependence of efficiency and stereoselectivity on the 3′-overhang length and sequence. Given a prebiotically plausible and compatible synthesis of aminoacyl phosphate mixed anhydrides, these results suggest that RNA molecules with acceptor stem termini resembling modern tRNAs could have been spontaneously aminoacylated, in a stereoselective and chemoselective manner, at their 2′,3′-diol termini prior to the onset of protein-catalyzed aminoacylation.

## Introduction

tRNA is the key adaptor molecule in the translation of a nucleic acid message into a protein sequence.^1^ Although the L-shaped 3D structure of tRNA and the way in which it functions in translation are now known in atomic detail, the reasons for tRNA being the size and shape it is are still unknown. Also unknown is how the two-step mechanism of tRNA aminoacylation via an aminoacyl-adenylate came to be. Although the idea that primitive tRNA might have been “its own activating enzyme” is very attractive as regards the emergence of translation,^2^ it has not yet received any experimental support.

**Figure 1:**
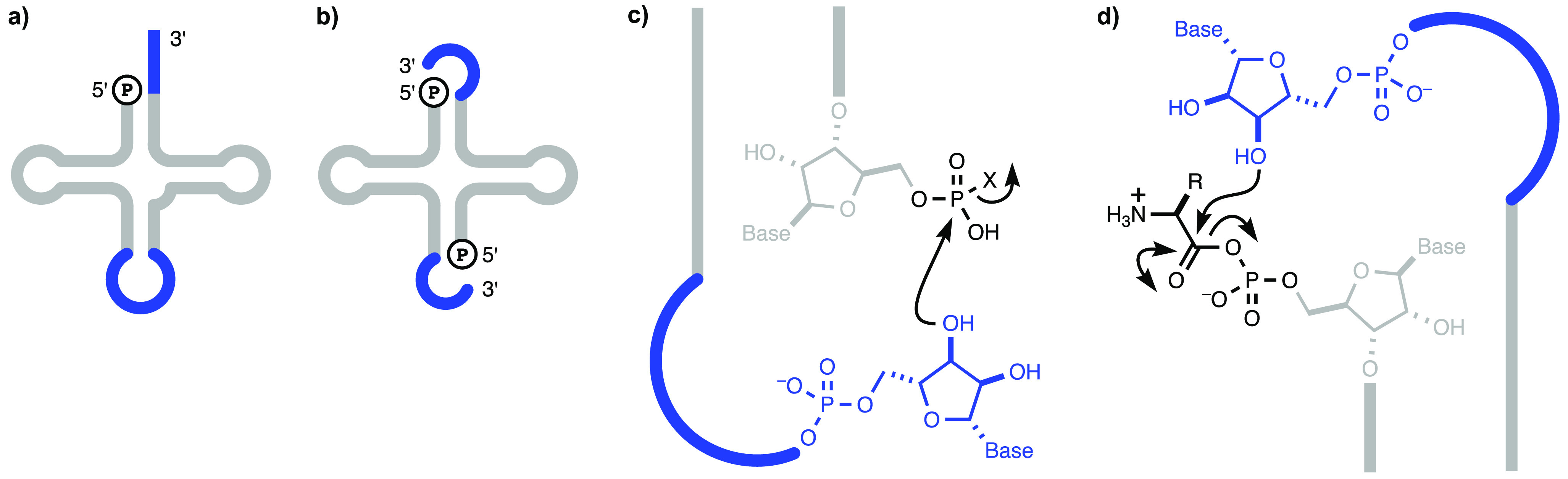
Model for the origin of tRNA by duplication suggests the possibility of interstrand aminoacyl transfer at the acceptor stem overhang. (a) Cloverleaf 2D structure of extant tRNA with the anticodon loop and extended conformation 3′-overhang highlighted (dark blue). (b) Two copies of RNA annealed to each other to generate a cloverleaf proto-tRNA with two folded-back conformation 3′-overhangs (dark blue). (c) Close up of the stem 3′-overhang at the bottom of the structure depicted in b showing the proximity that would be required for attack of the 3′-hydroxyl group on the activated 5′-phosphate (black) to generate a loop. (d) Close up of the stem 3′-overhang at the top of the structure depicted in b showing how the same proximity shown in c might allow transfer of an aminoacyl group from a mixed anhydride with the 5′-phosphate (black) to the 3′-hydroxyl group of the folded-back conformation 3′-overhang.

tRNA has a quasi-symmetric cloverleaf 2D structure (Figure 1a), and this along with the hint of symmetry derived from sequence alignment of the 5′-half and 3′-half^3−5^ and the phenomenon of split tRNA genes^6^ has led to suggestions that tRNA might have arisen by duplication.^3−5^ Such a duplication could occur in a number of ways,^5,7,8^ but we were intrigued by the suggestion that two copies of an RNA half the length of tRNA became joined end to end as this places the join in the anticodon loop (Figure 1b).^5,6^ The synthesis of aminoacyl phosphate mixed anhydrides has been demonstrated under prebiotically plausible conditions,^9−11^ and we now wondered if the proximity of the 3′- and 5′-termini that would be required to seal the anticodon loop by ligation might, at the symmetry-related acceptor termini of a proto-tRNA, allow transfer of an aminoacyl group from the 5′-phosphate to the 2′,3′-diol of a 3′-overhang with a folded-back conformation (Figure 1c and 1d). The proximity between the 3′- and the 5′-termini of a tRNA acceptor stem microhelix with a variant 3′-overhang has been demonstrated by NMR spectroscopy,^12^ supporting this idea.

In seminal work, Tamura and Schimmel demonstrated transfer of an aminoacyl group from the 5′-phosphate of a separate helper oligonucleotide to the 2′,3′-diol of a tRNA acceptor stem-overhang mimic using an additional bridging oligonucleotide that brought the reactive termini together in the form of a nicked duplex (Figure S1a).^13,14^ Although this work demonstrated that aminoacylation of a tRNA acceptor stem-overhang mimic is possible, we felt that it suffers from a number of shortcomings from an evolutionary perspective. First, it provides no explanation as to why the 3′-overhang of a tRNA should be the length and sequence it is, as any sequence of overhang longer than a few nucleotides could allow nicked duplex transfer using appropriate helper and bridging oligonucleotides. Second, there is no obvious way apparent for the sequence of the duplex to influence the side chain selectivity of the aminoacyl transfer—indeed, the aminoacyl transfer that was demonstrated for alanyl, leucyl, and phenylalanyl residues showed no chemoselectivity.^13^ Finally, it does not explain why, in extant biology, tRNA has a 5′-phosphate introduced by pre-tRNA cleavage in a reaction that has all the hallmarks of being the vestige of an ancient process.^15^ In the case of the aminoacyl transfer that we envisaged from a 5′-phosphate to the 2′,3′-diol of a folded-back 3′-overhang—hereinafter nicked loop transfer (Figure S1b)—the proximity required to facilitate aminoacyl transfer would derive from the structure of the acceptor stem-overhang mimic itself^12^ rather than requiring helper and bridging oligonucleotides. We felt that nicked loop transfer might better explain aspects of tRNA structure from an evolutionary perspective for several reasons. First, not all lengths and sequences of overhang are likely to adopt any of the range of conformations that one could imagine would be conducive to aminoacyl transfer. Finding a correlation between those that do and the nature of the tRNA 3′-overhang in extant biology would offer a hint as to why tRNA has the 3′-overhang length and sequence it does, though other functions could have influenced this, witness, for example, the binding of 3′-CCA termini to the 23S rRNA in extant biology.^16,17^ Second, the range of overhang conformations potentially allowing aminoacyl transfer would afford opportunities for both stereoselectivty and chemoselectivity resulting from interactions between the aminoacyl group and the RNA during transfer. Finally, were we to demonstrate nicked loop transfer from an aminoacyl mixed anhydride of the 5′-phosphate, it would suggest why tRNA ancestors might have had to have 5′-phosphate termini (Figure 1a and 1d), though again other functions could also have contributed to selection, for example, the role of the 5′-phosphate in peptidyl-tRNA hydrolysis.^18^

## Results and Discussion

Experimentally, we envisaged using HPLC to analyze aminoacyl transfer from a mixed anhydride donor strand to a complementary acceptor strand with a 3′-overhang. The large number of permutations of amino acid and 3′-overhang lengths and sequences coupled to a relatively low-throughput analytical method meant that we needed to establish starting points. On the basis of the idea that tRNA is the result of duplication, we designed an initial tRNA acceptor stem-overhang mimic by convergence from anticodon loop and acceptor stem-overhang lengths and sequences in extant biology (Figure S2). Our plan was then to modify the length and sequence of this initial mimic. Alanine was chosen as the first amino acid to investigate because it is one of the simplest of the chiral proteinogenic amino acids.

**Figure 2:**
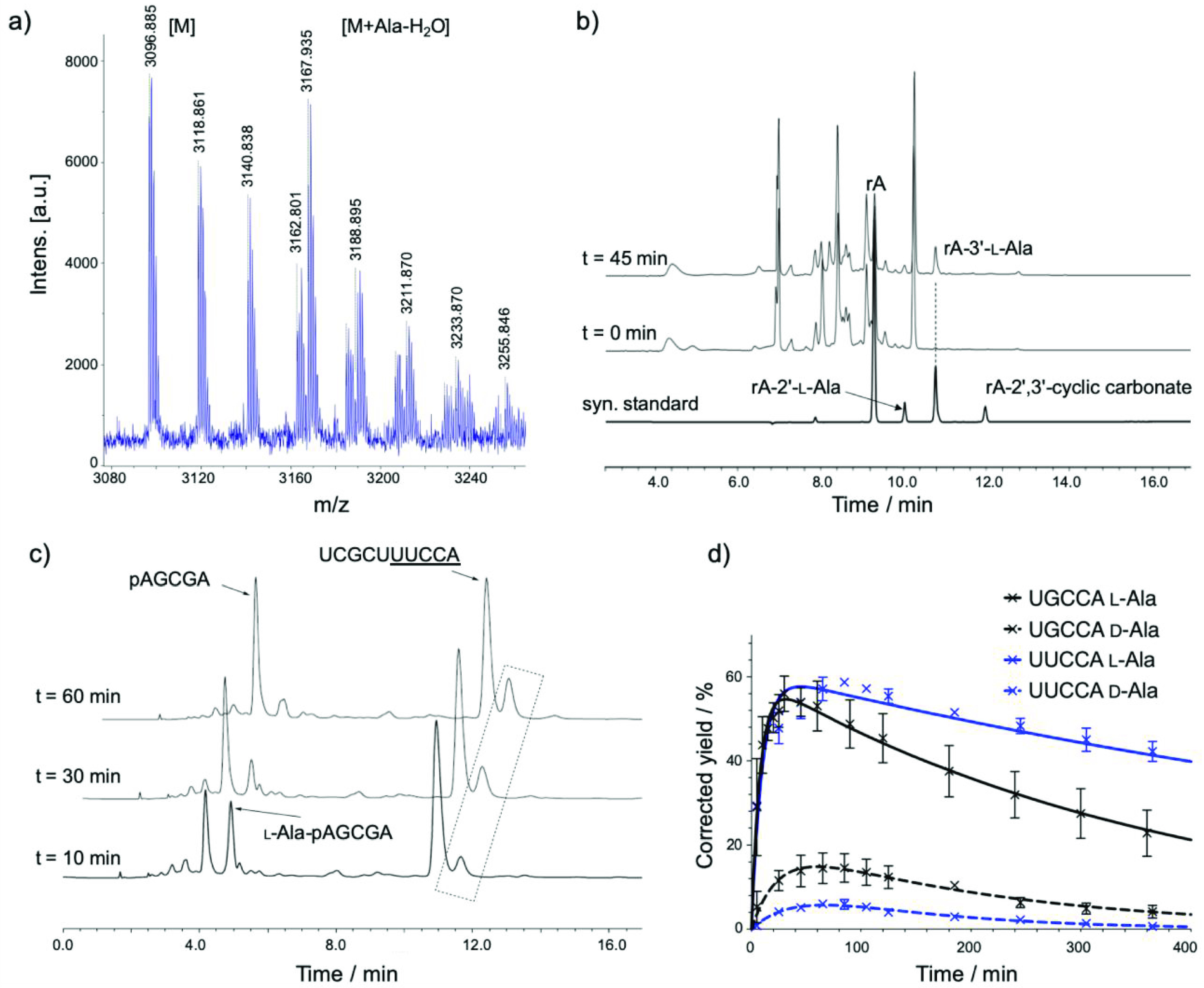
Characterization of nicked loop l-alanyl-transfer. (a) MALDI-TOF mass spectrum of products of aminoacyl transfer from 5′-l-Ala-pAGCGA-3′ to an acceptor strand with sequence 5′-UCGCUUGCCA-3′. Mass clusters for the acceptor strand (found 3096.885, calcd 3096.44) and its l-alanyl diol ester product (5′-UCGCUUGCCA-l-Ala, found 3167.935, calcd 3167.44) are indicated. Measured mass difference, 71.050, C_3_H_5_NO [Ala-H_2_O], calcd 71.037. (b) Enzyme digestion confirming that the diol of the acceptor strand’s 3′-terminal adenosine is aminoacylated by aminoacyl transfer. (c) l-Ala transfer in a tRNA acceptor arm mimic: 5′-UCGCUUUCCA-3′; 5′-l-Ala-pAGCGA-3′. Peaks for the donor, donor mixed anhydride, and acceptor strands are indicated. Peak presumed to be due to the diol ester transfer product is highlighted by the dashed box. (d) Time courses showing the stereoselectivity for l-Ala over d-Ala nicked loop transfer with acceptor strands UCGCUUGCCA and UCGCUUUCCA. Conditions: each oligoribonucleotide 100 μM, NaCl 100 mM, MgCl_2_ 5 mM, HEPES 50 mM, pH 6.8, 10 °C.

Conventional synthesis was used to aminoacylate a 5′-phosphorylated oligonucleotide (5′-pAGCGA-3′). The l-alanyl-phosphate mixed anhydride donor strand (5′-l-Ala-pAGCGA-3′) sample thereby prepared also contained the starting oligonucleotide either because the aminoacylation failed to proceed to completion or because of product hydrolysis. The mixed anhydride donor strand was then annealed to an acceptor strand (5′-UCGCUUGCCA-3′, underlining indicates overhang sequence) in a near neutral pH solution, optionally containing added salts (Supporting Information and Table S1). Aliquots of the reaction mixture were then taken over a period of time and analyzed by HPLC whereupon it became apparent that the peak for the mixed anhydride donor strand decreased over time (Figure S3). As this peak decreased, the peak for the 5′-phosphorylated donor strand (5′-pAGCGA-3′) increased, the peak for the acceptor strand decreased, and a new peak appeared. This new peak reached a maximum and then decreased, albeit more slowly than the peak for the mixed anhydride. This slower hydrolysis behavior was consistent with the peak being due to the aminoacyl-transfer product as aminoacyl esters are more stable than aminoacyl phosphate mixed anhydrides.^19^ A MALDI-TOF mass spectrum of the reaction mixture had peaks with masses consistent with both the unreacted acceptor strand and an alanyl derivative thereof (Figure 2a). Proof that the derivative was an l-alanyl ester of the 2′,3′-diol of the acceptor strand (and not an amide of a nucleobase amino group, or an ester of an internal 2′-hydroxyl group) was obtained by digesting the RNA in the reaction mixture with RNase A^19^ and comparing the HPLC elution profile of the degradation products with similar profiles for authentic standards of the 2′,3′-diol esters of adenosine with l- and d-alanine (Figure 2b and Figures S4–S6). Taken with the other data, the observation of the 2′,3′-diol esters of adenosine with l-alanine among the digestion products confirms that aminoacyl transfer takes place from the donor strand to the 2′,3′-diol terminus of the acceptor strand. We then screened the effects of added salts, pH, and temperature on the reaction (Table S1). It was found that under near optimal conditions (100 μM of each aminoacyl donor and acceptor strands, 100 mM NaCl, 5 mM MgCl_2_, 50 mM HEPES at pH 6.8, 10 °C), the observed yield of l-alanyl transfer was 30%. Taking into account the impurity of the mixed anhydride donor strand and its hydrolysis, the corrected yield was 55% (Table 1, Figure S3, Method).

**Table 1:**
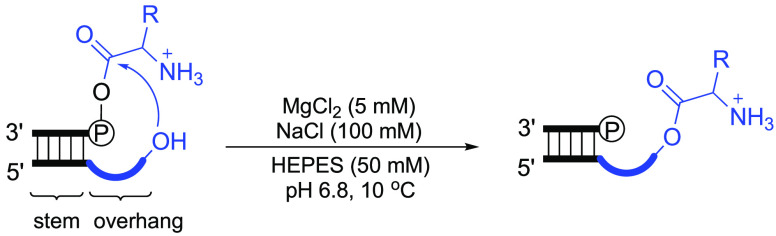
Nicked Loop Aminoacyl Transfer of tRNA Acceptor Arm Mimics^a^

	aminoacyl donor RNA strand					
	5′-l-Ala-pAGCGA		5′-d-Ala-pAGCGA		5′-Gly-pAGCGA	
acceptor RNA strand	observed yield	corrected yield	observed yield	corrected yield	observed yield	corrected yield
5′-UCGCUU**G**CCA-3′	30%	55%	10%	15%	9%	11%
5′-UCGCUU**A**CCA-3′	16%	30%	N.D.	N.D.	N.D.	N.D.
5′-UCGCUU**C**CCA-3′	N.D.	N.D.	N.D.	N.D.	N.D.	N.D.
5′-UCGCUU**U**CCA-3′	34%	57%	4%	6%	2%	3%
5′-UCGCUUGCC**C**-3′	N.D.	N.D.	N.D.	N.D.	N.D.	N.D.
5′-UCGCUUGCC**G**-3′	14%	25%	8%	12%	N.D.	N.D.
5′-UCGCUUGCC**U**-3′	N.D.	N.D.	N.D.	N.D.	N.D.	N.D.
5′-UCGCUUGCC**A**(2′d)-3′	4%	6%	–	–	–	–
5′-UCGCUUGCC**A**(3′d)-3′	N.D.	N.D.	–	–	–	–
5′-UCGCU**UGCCCA**-3′	22%	38%	15%	25%	17%	23%
5′-UCGCU**ACCA**-3′	N.D.	N.D.	N.D.	N.D.	N.D.	N.D.
5′-UCGCU**UCCA**-3′	1%	2%	2%	4%	N.D.	N.D.

a N.D.: product not detected. −:, experiments not performed.

We then proceeded to investigate the effect of overhang length and sequence and probe potential stereoselectivity and chemoselectivity of the transfer with d-alanine and other amino acids, but we first addressed a slight concern. Our random choice of G as the additional residue to insert in the overhang to make it five nucleotides long gave a sequence—UGCCA—that was partially self-complementary. Binding of two stem-overhang complexes to each other could therefore potentially generate a doubly nicked duplex where aminoacyl transfer in the configuration described by Tamura and Schimmel might be possible,^13^ although the C:C mismatch would be expected to significantly destabilize a duplex comprising two such overhangs arranged in an antiparallel fashion. Accordingly, we synthesized additional oligonucleotides in which the G of the overhang was changed to the other three canonical nucleotides, thereby removing self-complementarity. No transfer was detectable with the overhang with C in place of the G, but in the other two cases l-alanyl-transfer was efficient (corrected yields with the UACCA and UUCCA overhangs were 30% and 57%, respectively. Table 1, Figure 2c, Table S2, and Figures S7–S9). As the transfer with the UACCA or UUCCA overhangs must go through the folded-back conformation, these results suggest that the transfer with the UGCCA overhang similarly proceeds via the folded-back conformation as we envisaged.

We next addressed the stereochemistry question. Incubation of a conventionally synthesized d-alanyl-phosphate mixed anhydride donor strand (5′-d-Ala-pAGCGA-3′, along with 5′-pAGCGA-3′) with the acceptor strand (5′-UCGCUUGCCA-3′) resulted in only low amounts (≤15% corrected yield) of the aminoacyl-transfer product according to HPLC analysis (Table 1, Figure 2d, and Figure S10), giving an l:d stereoselectivity of ∼4:1. However, a considerably higher l:d stereoselectivity of ∼10:1 was observed when using an acceptor strand with the UUCCA overhang (Figure 2d and Figure S11), and the stereoselectivity with the UACCA overhang is likely similar (though we could not quantitate it because in this case the l-alanyl-transfer yield is lower and the d-alanyl-transfer product could not be detected) (Table 1). Tamura and Schimmel reported a stereoselectivity of ∼4:1 with the preference also being for transfer of l-configured aminoacyl residues, but in their case, for synthetic expediency, the donor strand was composed of DNA (Figure S1a).^13^ We measured the l:d stereoselectivity for nicked duplex transfer in an all-RNA system and found it to be ∼2:1 (Table S3 and Figures S12–S14).

Moving on to the question of whether there is any aminoacyl side chain chemoselectivity in the transfer, we opted to investigate glycyl-transfer next. This decision was predicated on the expectation that as glycine is likely to have been one of the most abundant prebiotic amino acids,^20,21^ transfer of other aminoacyl residues would have had to contend with significant competing glycyl transfer. We studied glycyl transfer using the same four acceptor strands that we had used before (Table 1 and Table S4). With the UCCCA acceptor strand there was again no detectable transfer, and while there was transfer to the UGCCA (11% corrected, Figures S15 and S16) and UUCCA (3% corrected) acceptor strands with glycyl donor, both were lower than in the case of l-alanyl transfer. Intriguingly, we observed no transfer of a glycyl residue with the UACCA acceptor strand, in stark contrast to the good transfer yield of an l-alanyl residue (30% corrected). Transfer to the UUCCA and UACCA acceptor strands is thus both highly stereoselective for an l-alanyl residue over a d-alanyl residue and highly chemoselective for transfer of an l-alanyl residue over a glycyl residue (Table 1). In addition, we found that nicked duplex transfer of a glycyl residue in our all*-*RNA system still took place in reasonable yield (17% corrected, Figure S17), thus strengthening the case for nicked duplex transfer being significantly less chemoselective than nicked loop transfer.

We then changed our focus to the 3′-terminal nucleotide of the overhang. We synthesized three more acceptor strands based on our original UGCCA acceptor strand but in which the 3′-terminal A was changed to the other canonical nucleotides. We found that l-alanyl transfer to the acceptor strand with a UGCCG overhang was still reasonably efficient (25% corrected, Figure S18), and d-alanyl transfer was less so, but glycyl transfer was not detected. No alanyl or glycyl transfer with pyrimidine-terminated acceptor strands was detected. The preference for a purine at the 3′-terminus of the acceptor strand is thus apparent, and we suggest that it might be explained by the stabilization of the folded-back conformation by stacking of the nucleobase of the last overhang residue on top of the last base pair of the stem. In an attempt to get an indication of the regioselectivity of transfer to the 2′,3′-diol, we employed deoxynucleotide-terminated acceptor strands, but no significant aminoacyl transfer was observed with either a UGCC-2′-dA overhang or a UGCC-3′-dA overhang (less than 6%, Figures S19 and S20).

Lastly, we tried to interrogate the effect of overhang length on the nicked loop aminoacyl transfer. With an acceptor strand having a UGCCCA overhang, we observed transfer of l-alanyl, d-alanyl, and glycyl residues in corrected yields of 38%, 25%, and 23%, respectively (Table 1, Figures S21–S23). When we shortened the overhang to ACCA or UCCA, there was no significant transfer of either l- or d-alanyl residues or glycyl residues (no more than 4% corrected yield, Table 1 and Figures S24 and S25). We therefore conclude from a sparse sampling of different aminoacyl groups and overhang sequence space that efficient chemo- and stereoselective nicked loop transfer in a tRNA acceptor stem-overhang mimic is possible with acceptor strands having overhangs of five or six nucleotides. The stereo- and chemoselectivity of aminoacyl transfer is dependent on the nature of the overhang sequence. However, ribosomal peptide synthesis uses not only aminoacyl tRNA but also *N*-acylaminoacyl tRNA, so we now switched our attention to investigate whether such *N*-acylaminoacyl species could also be produced by interstrand transfer in a tRNA acceptor stem-overhang mimic.

We started our investigation of *N*-acylaminoacyl transfer using a conventionally synthesized *N*-acetylglycyl phosphate mixed anhydride (5′-Ac-Gly-pAGCGA-3′) as the donor strand. Incubation of an acceptor strand (5′-UCGCUUGCCA-3′) with the donor strand was again monitored by HPLC time course analysis (Methods and Supporting Information). Under optimal conditions, the transfer to the UGCCA overhang proceeded in good yield (32% observed, 46% corrected, Table 2, Tables S5 and S6, and Figures S26–S28). Changing the second base of the overhang had little effect on yield in complete contrast to the case with the transfer of an unprotected glycyl residue (Table 2 and Figures S29–S31). In particular, for the acceptor strand with a UCCCA overhang, very efficient *N*-acetylglycyl transfer was observed (36% observed, 46% corrected, Figure S31). Changing the last base of the overhang showed that a purine is again important for efficient transfer (Table 2 and Figures S32–S34). Increasing the length was tolerated, the UGCCCA overhang undergoing *N*-glycyl transfer in good yield (32% corrected, Table 2 and Figure S35). Decreasing the length was now also tolerated, the UCCA overhang undergoing transfer of an *N*-acetylglycyl residue in 37% corrected yield (Figure S36), although the ACCA overhang was a poor substrate (6% corrected, Figure S37). The better aminoacyl transfer yield with a UCCA overhang relative to an ACCA overhang correlates with the dominant folded-back conformation of the former relative to the latter.^12^ Efficient transfer was also observed to deoxy-terminated acceptor strands. On this occasion, both UGCC-2′-dA and UGCC-3′-dA overhangs underwent efficient reaction (23% and 19% corrected, respectively, Figures S38 and S39), suggesting that transfer to the diol-terminated overhang of the same sequence might not be regioselective. The significant difference between the transfer of *N*-acetylglycyl residues and of unprotected glycyl residues indicates that the protonated amino group of the latter might play an important role in this process, possibly through intramolecular salt-bridge formation with attendant conformational effects.

**Table 2:**
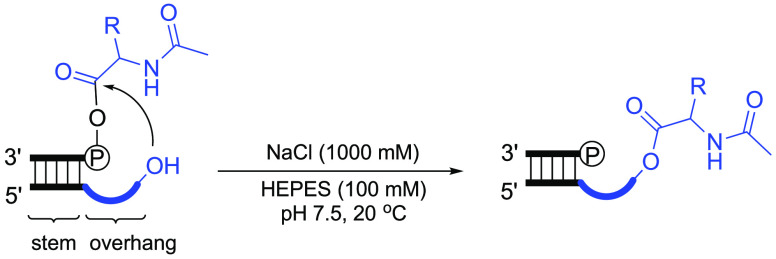
Nicked Loop *N*-Acetylaminoacyl Transfer of tRNA Acceptor Arm Mimics^a^

	*N*-acetylaminoacyl donor RNA strand			
	5′-Ac-l/d-Ala-pAGCGA		5′-Ac-Gly-pAGCGA	
acceptor RNA strand	observed yield	corrected yield	observed yield	corrected yield
5′-UCGCUU**G**CCA-3′	20%	25%	32%	46%
5′-UCGCUU**A**CCA-3′	9%	10%	27%	36%
5′-UCGCUU**C**CCA-3′	8%	9%	36%	46%
5′-UCGCUU**U**CCA-3′	14%	16%	21%	28%
5′-UCGCUUGCC**C**-3′	N.D.	N.D.	5%	7%
5′-UCGCUUGCC**G**-3′	12%	13%	25%	36%
5′-UCGCUUGCC**U**-3′	N.D.	N.D.	5%	7%
5′-UCGCUUGCC**A**(2′d)-3′	3%	4%	17%	23%
5′-UCGCUUGCC**A**(3′d)-3′	2%	2%	14%	19%
5′-UCGCU**UGCCCA**-3′	11%	14%	20%	32%
5′-UCGCU**ACCA**-3′	10%	13%	23%	37%
5′-UCGCU**UCCA**-3′	2%	3%	4%	6%

a N.D.: product not detected.

Intrigued, we next dealt with stereochemistry issues associated with *N*-acetylalanyl transfer. Activation of the carboxylate group of *N*-acyl amino acids can lead to the formation of 5(4*H*)-oxazolones that are prone to racemization via their enol tautomers.^11,22^ Accordingly, we first developed coupling chemistry which largely circumvents this issue and thereby prepared *N*-acetyl-l-alanyl-phosphate and *N*-acetyl-d-alanyl-phosphate mixed anhydride donor strands (5′-Ac-l-Ala-pAGCGA-3′ and 5′-Ac-d-Ala-pAGCGA-3′) starting from either enantiomer of *N*-acetylalanine or a mixture of both (5′-Ac-l/d-Ala-pAGCGA-3′) starting from the racemate^11^ (see Supporting Information for details). We then incubated the donor strand 5′-Ac-l/d-Ala-pAGCGA-3′ with a UGCCA overhang acceptor strand and observed transfer products in an overall 25% corrected yield (Table 2, Tables S7 and S8, and Figure S40). Digestion of the transfer products with RNase A followed by HPLC analysis revealed that the 2′,3′-diol esters of adenosine with *N*-acetyl-l-alanine had approximately the same abundance as the 2′,3′-diol esters of adenosine with *N*-acetyl-d-alanine (Figures S41–S43). Digestion of the transfer products from the 5′-Ac-l-Ala-pAGCGA-3′ donor strand with RNase followed by HPLC analysis revealed that the 2′,3′-diol esters of adenosine with *N*-acetyl-l-alanine greatly predominated over those of *N*-acetyl-d-alanine, the latter resulting from a slight loss of enantiomeric purity during preparation of the mixed anhydride. Similarly, using the 5′-Ac-d-Ala-pAGCGA-3′ donor strand gave predominantly the 2′,3′-diol esters of adenosine with *N*-acetyl-d-alanine after RNase digestion. Furthermore, using a 1:1 mixture of both donor strands (made by mixing an equal amount of separately made 5′-Ac-l-Ala-pAGCGA-3′ and 5′-Ac-d-Ala-pAGCGA-3′) resulted in approximately equal amounts of the 2′,3′-diol esters of adenosine with *N*-acetyl-l-alanine and *N*-acetyl-d-alanine after RNase A digestion (Figures S41–S43). These experiments showed that there is no stereoselectivity in the transfer of *N*-acetyl-alanyl residues to the acceptor stand with the UGCCA overhang, which differs significantly from the high stereoselectivity observed in the transfer of unacylated alanyl residues. The transfer still took place when the G of the UGCCA overhang was changed to the other three canonical bases, and again, it was found that there was a big preference for a purine at the last position (Table 2 and Figures S44–S49). Finally, the effect of overhang length on the transfer of *N*-acetylalanyl was similar to the case with *N*-acetylglycyl transfer (Table 2 and Figures S50–S52).

## Conclusions

On the basis of a model for the origin of tRNA by duplication^3,5,6^ and structural data on acceptor stem variants,^12^ a range of tRNA acceptor stem-overhang mimics was found to undergo interstrand aminoacyl and *N*-acetylaminoacyl transfer without the need for other oligonucleotides or auxiliaries.

Depending on the nature of the aminoacyl residue and the overhang length and sequence, the transfer reactions can be highly stereoselective and chemoselective as well as efficient. The prebiotic formation of aminoacyl phosphate mixed anhydrides is feasible,^9,10^ and taken together with the aminoacyl-transfer process revealed herein, this suggests that tRNA acceptor stems or their forerunners could have become aminoacylated before the evolution of aminoacyl-tRNA synthetase ribozymes or enzymes. This is consistent with early speculation that primitive tRNA might have been “its own activating enzyme”.^2^ Furthermore, the chemistry of the transfer reaction closely resembles the second step of the reaction catalyzed by aminoacyl-tRNA synthetase enzymes^23^ and could have foreshadowed it evolutionarily in keeping with the principle of continuity.^24^

Although the generation of selectively aminoacylated tRNA-like molecules is a necessary prelude to coded peptide synthesis, it is not sufficient as aminoacyl transfer from one such species to the aminoacyl group of another would generate a dipeptidyl-RNA that would be extremely prone to diketopiperazine loss.^25^ Thus, the finding that *N*-acylaminoacyl residues can similarly be transferred is also important—the prebiotic synthesis of *N*-acylaminoacyl phosphate mixed anhydrides also is feasible^11^ (see also Supporting Information). Development of a high-throughput assay will allow the aminoacyl-transfer reaction to be systematically explored across stem and overhang sequence space for all of the canonical amino acids or a subset thereof. This should shed further light on potential stereochemical relationships between amino acid side chains and RNA sequences.^2,7,24,26^

Finally, we suggest that the chemistry we uncovered might partly explain certain structural aspects of tRNA. The most striking one is the overall shape of the tRNA acceptor arm, especially the characteristic termini. The processing of tRNA precursors by RNase P to generate a 5′-phosphate is clearly ancient, and yet the 5′-phosphate still plays a role in tRNA function. We suggest that the 5′-phosphate had a crucial functional role in early aminoacylation chemistry, and when the transition to the modern mechanism of aminoacylation took place, the 5′-phosphate adapted to novel functions in the Central Dogma. We further suggest an ancestral function for a 3′-overhang of 4–7 nucleotides and a sequence with a U at the beginning and a purine at the end, allowing adoption of a folded-back conformation^12^ that stacked on the last base pair of the stem and enabled nicked loop aminoacyl transfer. We speculate that during the evolutionary transition from using an aminoacyl mixed anhydride of the 5′-phosphate of the tRNA acceptor arm to using an aminoacyl adenylate, an overhang was retained at the 3′-terminus but its length and sequence underwent a slight change to give the near-canonical ACCA of extant biology.^5^

## References

[ref1] Crick F. H. C. The genetic code – yesterday, today, and tomorrow. Cold Spring Harbor Symp. Quant. Biol. 1966, 31, 3–9. 10.1101/SQB.1966.031.01.007. 5237190

[ref2] Crick F. H. C. The origin of the genetic code. J. Mol. Biol. 1968, 38, 367–379. 10.1016/0022-2836(68)90392-6. 4887876

[ref3] Di Giulio M. On the origin of the transfer RNA molecule. J. Theor. Biol. 1992, 159, 199–214. 10.1016/S0022-5193(05)80702-7. 1294846

[ref4] Widmann J.; Di Giulio M.; Yarus M.; Knight R. tRNA Creation by hairpin duplication. J. Mol. Evol. 2005, 61, 524–530. 10.1007/s00239-004-0315-1. 16155749

[ref5] Di Giulio M. The origin of the tRNA molecule: Independent data favor a specific model of its evolution. Biochimie 2012, 94, 1464–1466. 10.1016/j.biochi.2012.01.014. 22305822

[ref6] Randau L.; Münch R.; Hohn M. J.; Jahn D.; Söll D. *Nanoarchaeum equitans* creates functional tRNAs from separate genes for their 5′- and 3′-halves. Nature 2005, 433, 537–541. 10.1038/nature03233. 15690044

[ref7] Hopfield J. J. Origin of the genetic code: a testable hypothesis based on tRNA structure, sequence, and kinetic proofreading. Proc. Natl. Acad. Sci. U. S. A. 1978, 75, 4334–4338. 10.1073/pnas.75.9.4334. 279919 PMC336109

[ref8] Eigen M.; Winkler-Oswatitsch R. Transfer-RNA, an Early Gene?. Naturwissenschaften 1981, 68, 282–292. 10.1007/BF01047470. 7266675

[ref9] Biron J. P.; Parkes A. L.; Pascal R.; Sutherland J. D. Expeditious, potentially primordial, aminoacylation of nucleotides. Angew. Chem., Int. Ed. 2005, 44, 6731–6734. 10.1002/anie.200501591. 16187390

[ref10] Leman L. J.; Orgel L. E.; Ghadiri M. R. Amino acid dependent formation of phosphate anhydrides in water mediated by carbonyl sulfide. J. Am. Chem. Soc. 2006, 128, 20–21. 10.1021/ja056036e. 16390101

[ref11] Liu Z.; Wu L.-F.; Xu J.; Bonfio C.; Russell D. A.; Sutherland J. D. Harnessing chemical energy for the activation and joining of prebiotic building blocks. Nat. Chem. 2020, 12, 1023–1028. 10.1038/s41557-020-00564-3. 33093680 PMC7610406

[ref12] Puglisi E. V.; Puglisi J. D.; Williamson J. R.; RajBhandary U. L. NMR analysis of tRNA acceptor stem microhelices: Discriminator base affects tRNA conformation at the 3′-end. Proc. Natl. Acad. Sci. U. S. A. 1994, 91, 11467–11471. 10.1073/pnas.91.24.11467. 7972085 PMC45252

[ref13] Tamura K.; Schimmel P. Chiral-selective aminoacylation of an RNA minihelix. Science 2004, 305, 1253 10.1126/science.1099141. 15333830

[ref14] Tamura K.; Schimmel P. Chiral-selective aminoacylation of an RNA minihelix: Mechanistic features and chiral suppression. Proc. Natl. Acad. Sci. U. S. A. 2006, 103, 13750–13752. 10.1073/pnas.0606070103. 16950872 PMC1564265

[ref15] Altman S. Ribonuclease P.. Philos. Trans. R. Soc., B 2011, 366, 2936–2941. 10.1098/rstb.2011.0142. PMC3158923 21930585

[ref16] Bokov K.; Steinberg S. V. A hierarchical model for evolution of 23S ribosomal RNA. Nature 2009, 457, 977–980. 10.1038/nature07749. 19225518

[ref17] Petrov A. S.; Gulen B.; Norris A. M.; Kovacs N. A.; Bernier C. R.; Lanier K. A.; Fox G. E.; Harvey S. C.; Wartell R. M.; Hud N. V.; Williams L. D. History of the ribosome and the origin of translation. Proc. Natl. Acad. Sci. U. S. A. 2015, 112, 15396–15401. 10.1073/pnas.1509761112. 26621738 PMC4687566

[ref18] Fromant M.; Plateau P.; Schmitt E.; Mechulam Y.; Blanquet S. Receptor site for the 5′-phosphate of elongator tRNAs governs substrate selection by peptidyl-tRNA hydrolase. Biochemistry 1999, 38, 4982–4987. 10.1021/bi982657r. 10213600

[ref19] Zachau H. G.; Acs G.; Lipmann F. Isolation of adenosine amino acid esters from a ribonuclease digest of soluble, liver ribonucleic acid. Proc. Natl. Acad. Sci. U. S. A. 1958, 44, 885–889. 10.1073/pnas.44.9.885. 16590285 PMC528662

[ref20] Johnson A. P.; Cleaves H. J.; Dworkin J. P.; Glavin D. P.; Lazcano A.; Bada J. L. The Miller volcanic spark discharge experiment. Science 2008, 322, 404 10.1126/science.1161527. 18927386

[ref21] Patel B. H.; Percivalle C.; Ritson D. J.; Duffy C. D.; Sutherland J. D. Common origins of RNA, protein and lipid precursors in a cyanosulfidic protometabolism. Nat. Chem. 2015, 7, 301–307. 10.1038/nchem.2202. 25803468 PMC4568310

[ref22] Slebioda M.; St-Amand M. A.; Chen F. M. F.; Benoiton N. L. Studies on the kinetics of racemization of 2,4-disubstituted-5(4*H*)-oxazolones. Can. J. Chem. 1988, 66, 2540–2544. 10.1139/v88-398.

[ref23] Freist W. Mechanisms of aminoacyl-tRNA synthetases: A critical consideration of recent results. Biochemistry 1989, 28, 6787–6795. 10.1021/bi00443a001. 2684265

[ref24] Orgel L. E. Evolution of the genetic apparatus. J. Mol. Biol. 1968, 38, 381–393. 10.1016/0022-2836(68)90393-8. 5718557

[ref25] Weber A. L.; Orgel L. E. Poly(U)-directed peptide-bond formation from the 2′(3′)-glycyl esters of adenosine derivatives. J. Mol. Evol. 1980, 16, 1–10. 10.1007/BF01732065. 7441777

[ref26] Woese C. R. The Genetic Code; Harper & Row: New York, 1967; pp 128–129.

