# Supplementary material for: Interstrand Aminoacyl Transfer in a tRNA Acceptor Stem-Overhang Mimic
Source: J Am Chem Soc. 2021 Jul 20;143(30):11836–42. doi: 10.1021/jacs.1c05746 (PMC8397310; doi:10.1021/jacs.1c05746)
Supplement: Supplementary file 1 — ja1c05746_si_001.pdf [file ja1c05746_si_001.pdf]

Supplementary Materials for

INTERSTRAND AMINOACYL-TRANSFER IN A TRNA ACCEPTOR STEM-OVERHANG MIMIC

Long-Fei Wu[1]‡†, Meng Su[1]‡, Ziwei Liu[1], Samuel J. Bjork[1] and John D. Sutherland[1]*

*Correspondence to: johns@mrc-lmb.cam.ac.uk

This PDF file includes:

Materials and Methods
Supplementary Text
Figs. S1 to S53
Tables S1 to S8
References 26-27

Materials and Methods

Reagents and solvents were obtained from *Acros Organics*, *Alfa Aesar*, *Santa Cruz Biotechnology*, *Sigma-Aldrich*, *SYNTHON Chemicals GmbH & Co. KG* and *VWR International*, and were used without further purification unless otherwise stated. For solid phase RNA synthesis, primer Support 5G for A, G, C, U or 2'-dA (with loading ~300 µmol/g) were purchased from GE Healthcare. 3'-dA-CPG (with loading 50 µmol/g, item number 20-2004-01) was purchased from Glen Research. Phosphoramidites for RNA synthesis were purchased from Sigma-Aldrich or Link Technologies. RNA oligomers used in this study were synthesized using an ÄKTA™ oligopilot™ plus 10 (*GE Healthcare*) on a 5 to 50 µmol scale. *MettlerToledo* SevenEasy pH Meter S20 combined with a *ThermoFisher Scientific* Orion 8103BN Ross semi-micro pH electrode was used to measure and adjust the pH to the desired value. ^{1}H-, ^{31}P-, and ^{13}C-nuclear magnetic resonance (NMR) spectra were acquired using a *Bruker* Ultrashield 400 Plus or *Bruker* Ascend 400 operating at 400.13, 161.97, and 100.62 MHz, respectively. Samples consisting of H_2O/D_2O mixtures were analyzed using HOD suppression to collect ^{1}H-NMR spectroscopy data. The notations s, d, t, and q represent the multiplicities singlet, doublet, triplet, and quartet, respectively. Chemical shifts (δ) are shown in ppm. Mass spectra were acquired on an *Agilent* 1200 LC-MS system equipped with an electrospray ionization (ESI) source and a 6130 quadrupole spectrometer (LC solvents: A, 0.2 % formic acid in H_2O – B, and 0.2 % formic acid in acetonitrile), or on a Bruker Ultraflex III MALDI-TOF. High-Pressure Liquid Chromatography (HPLC) was run on Dionex Ultimate 3000 (Thermo Scientific) using an Atlantis™ T3, 5 µm, 4.6 x 250 mm column or an Atlantis™ T3, 3 µm, 4.6 x 150 mm column. Oligonucleotide concentrations were determined by UV absorbance at 260 nm using a NanoDrop® ND-1000 spectrophotometer.

Chemical synthesis of RNA oligomers

After automated synthesis, RNAs were first cleaved from the solid support by treating with 3 mL of a 1:1 mixture of 28% wt NH_3/H_2O solution and 33% wt CH_3NH_2/EtOH solution at 55 °C for 30 minutes in a tube with a sealed cap. The solid was removed by filtration and washed with 50 % EtOH/H_2O. The solution and washings were combined and evaporated to dryness under reduced pressure. Silyl protecting groups were then removed by treating the residues with 3 mL of 1:1 mixture of triethylamine trihydrofluoride and DMSO at 55°C for 90 minutes in a tube with a sealed cap. After brief cooling at -32 °C, 30 mL of cold 50 mM $NaClO_4$ in acetone was added to the solution to precipitate the RNA product. The resulting mixture was centrifuged and the pellet of RNA was re-dissolved in 10 mL of water and passed through a

Waters Sep-Pack C18 Cartridge, 5 g sorbent (Cartridge was pre-washed with 20 mL of MeOH then 100 mL of water before sample loading, then washed with 100 mL of H_2O, 20 mL of 10 % MeOH/H_2O, 25 mL of 20 % MeOH/H_2O, 25 mL of 50 % MeOH/H_2O and 20 mL of MeOH sequentially). Eluates containing RNA were combined and lyophilized. The resulting RNA was stored as a solid or dissolved in neutral pH solution at -32 °C for future usage.

Chemical synthesis of *N*-4-monomethoxytritylglycine (MMtr-Gly-OH) and *N*-tritylalanine (Trt-Ala-OH)

Trimethylsilyl chloride (1.27 mL, 10 mmol) was added to a magnetically stirred suspension of amino acid (10 mmol) in chloroform/acetonitrile (18 mL, v/v 5/1) at room temperature. The reaction mixture was heated under reflux for 2 h then allowed to cool to room temperature. A chloroform solution (10 mL) of triethylamine (2.79 mL, 20 mmol) and 4-monomethoxytrityl chloride or trityl chloride (10 mmol, 1 eq.) was added to the mixture. The reaction was further stirred at room temperature for 1 hour before the addition of methanol (2 mL, 50 mmol). The resulting mixture was evaporated to dryness and the residue was treated with a mixture of diethyl ether (50 mL) and a pre-cooled aqueous solution of citric acid (5%, 50 mL). The organic phase was separated and washed with 1 M NaOH solution (2 × 20 mL) and water (2 × 20 mL). The aqueous layers were combined and neutralized to pH = 7 with concentrated HCl on ice, then extracted using diethyl ether. The organic phase was dried over Na_2SO_4, and concentrated to give the desired product as a yellowish or white foam.

N-4-monomethoxytritylglycine (**MMTr-Gly-OH**), 55% yield.

^{1}H NMR (400 MHz, d_5-pyridine) δ = 7.50 (4H, m, MMTr), 7.34 (4H, m, MMTr), 7.10 (4H, m, MMTr), 7.00 (2H, m, MMTr), 3.42 (3H, s, OC$\boldsymbol{H_3}$), 3.28 (2H, s, C$\boldsymbol{H_2}$COOH)

^{13}C NMR (100 MHz, d_5-pyridine) δ = 174.5 (**C**OOH), 158.3 (C4'), 146.7 (2×C1), 138.2 (C1'), 130.2 (2×C4), 128.8 (2×C3, 2×C5), 128.1 (2×C2, 2×C5), 126.5 (C2', C6'), 113.5 (C3', C5'), 70.6 (**C**Ph), 54.9 (O**C**H_3), 46.5 (**C**H_2COOH).

Mass: $C_{22}H_{19}N_1O_3^-$ [M-H$^+$]: calc. 346.2, found 346.1.

N-trityl-L-alanine (**Trt-L-Ala-OH**), 52% yield.

^{1}H NMR (400 MHz, $CDCl_3$) δ = 7.34 (6H, m, Trt), 7.19 (6H, m, Trt), 7.13 (3H, m, Trt), 3.33 (1H, q, J = 7.1 Hz, C**H**CH_3), 1.16 (3H, J = 7.1 Hz, C$\boldsymbol{H_3}$).

^{13}C NMR (100 MHz, $CDCl_3$) δ = 178.8 (***C***OOH), 145.4 (3×C1), 128.7 (3×C3, 3×C5), 128.0 (3×C2, 3×C6), 126.8 (3×C4), 71.5 (***C***Ph), 52.5 (***C***HCH_3), 21.0 (CH***C***H_3).
Mass: $C_{22}H_{20}N_1O_2^-$ [M-H$^+$]: calc. 330.2, found 330.1.

N-trityl-D-alanine (**Trt-D-Ala-OH**), 53% yield.
^{1}H NMR (400 MHz, $CDCl_3$) δ = 7.30 (6H, m, Trt), 7.09 (6H, m, Trt), 7.01 (3H, m, Trt), 3.16 (1H, q, *J* = 7.0 Hz, C***H***CH_3), 1.02 (3H, d, *J* = 7.0 Hz, C***H₃***).
^{13}C NMR (100 MHz, $CDCl_3$) δ = 179.1 (***C***OOH), 145.9 (3×C1), 128.8 (3×C3, 3×C5), 127.9 (3×C2, 3×C6), 126.5 (3×C4), 71.5 (***C***Ph), 52.7 (***C***HCH_3), 21.4 (CH***C***H_3).
Mass: $C_{22}H_{20}N_1O_2^-$ [M-H$^+$]: calc. 330.2, found 330.1.

Chemical synthesis of RNA mixed anhydride with *N*-trityl or *N*-4-monomethoxytrityl amino acid (Trt-Ala-pAGCGA or MMTr-Gly-pAGCGA)

To a mixture of *N*-tritylalanine or *N*-4-monomethoxytritylglycine (5.9 μmol, 2 eq.), *N*-(3-dimethylaminopropyl)-*N*′-ethylcarbodiimide hydrochloride (EDCI, 11.2 mg, 58.6 μmol, 20 eq.) and 5'-pAGCGA (4.8 mg, 2.9 μmol, 1 eq.) was added pyridine (600 μL) and formamide (300 μL). The resulting suspension was stirred on an ice bath for 30 min. Then the solution was then centrifuged, and the clarified solution was transferred to another tube. A pre-cooled acetone solution of $NaClO_4$ (50 mM, 3.5 mL) was added to the solution to precipitate the products. The pellet of product was collected by centrifugation and was briefly dried under vacuum to remove the remaining acetone and pyridine. The white solid was immediately stored dry at -70°C until use.

Chemical synthesis of RNA mixed anhydride with *N*-acetyl amino acid (Ac-Gly-pAGCGA, or Ac-L-Ala-pAGCGA or Ac-D-Ala-pAGCGA)

300 μL of an aqueous solution of pAGCGA (1 mM) and *N*-acetyl amino acid (200 mM, *N*-Ac-Gly, or *N*-Ac-L-Ala, or *N*-Ac-D-Ala, or 1:1 *N*-Ac-L-Ala and *N*-Ac-D-Ala) was adjusted to pH 4.8 using 5 M HCl and 5 M NaOH. Methyl isonitrile (6 μL, 350 mM) was added and the solution was vigorously shaken before being frozen at -32 °C. To monitor progress of the reaction, aliquots (2 μL) were taken over time by temporarily thawing the sample and added to 18 μL of H_2O. 10 μL of the resulting mixture was analyzed by HPLC to quantify the accumulation of RNA mixed anhydride with *N*-acetyl amino acid (Ac-Gly-pAGCGA or Ac-Ala-pAGCGA) using UV detection at 260 nm. Yields of the desired mixed anhydride of between 60 % to 90 % were determined after 30 days incubation at -32 °C. The solution was

kept in the frozen state and being used directly as a stock solution for further experiments. (During the synthesis of Ac-L-Ala-pAGCGA or Ac-D-Ala-pAGCGA, there was minimal racemization/epimerization of the *N*-acylalanyl moiety as inferred following the *N*-acylalanyl transfer reactions by RNase digestion and HPLC analysis).

Chemical synthesis of standard products of 2'/3'-aminoacyl adenosine (Ade-2'/3'-Ala or Ade-2'/3'-Gly)

A solution of adenosine (1.3 mg, 4.9 μmol) in 25 μL DMSO was added to 425 μL of water in a 1.5 mL Eppendoff tube, and the resulting mixture was heated using a heat-gun until a clear solution was obtained. In another 1.5 mL Eppendoff tube, *N*-Boc-aminoacid (4.8 mg for *N*-Boc-glycine, 5.2 mg for *N*-Boc-L-alanine, or 5.2 mg for *N*-Boc-D-alanine respectively, 27.4 μmol) was stirred in 250 μL acetonitrile, and carbonyldiimidazole (CDI, 4.4 mg, 27.4 μmol) was added. The resulting acetonitrile solution was vortexed for 3 min at room temperature and then 50 μL of it was added to the previously made adenosine solution. The resulting mixture was shaken for 10 min at room temperature and then adjusted to pH 4 using 1 M HCl before evaporation to dryness under reduced pressure. Trifluoroacetic acid (TFA, 200 μL) was added and the solution was shaken at room temperature for 10 min. TFA was then removed by evaporation under reduced pressure. 500 μL of D_2O was added to the residue to give a pD ~ 1 solution. The formation of the desired products (Ade-2'/3'-L-Ala, Ade-2'/3'-D-Ala, or Ade-2'/3'-Gly respectively) was confirmed by direct ^{1}H-NMR analysis.

Chemical synthesis of standard products of 2'/3'-(*N*-acetyl) aminoacyl adenosine (Ade-2'/3'-Ala-Ac or Ade-2'/3'-Gly-Ac)

Adenosine (1.3 mg, 4.9 μmol) in 25 μL DMSO was added to 425 μL of water (50 μL D_2O and 375 μL of H_2O) in a 1.5 mL Eppendoff tube, and the resulting mixture was heated using a heat-gun until a clear solution was obtained. In another 1.5 mL Eppendoff tube, *N*-acetyl amino acid (3.2 mg for *N*-acetylglycine, 3.5 mg for *N*-acetyl-L-alanine, or 3.5 mg for *N*-acetyl-D-alanine respectively, 27.4 μmol) was stirred in 250 μL acetonitrile, and carbonyldiimidazole (CDI, 4.4 mg, 27.4 μmol) was added. The resulting acetonitrile solution was then vortexed for 3 min at room temperature and then 50 μL of it was added to the previously made adenosine solution. The resulting mixture was shaken for 10 min at room temperature. The pH of this solution was found to be ~ 7.2. The formation of the desired products (Ade-2'/3'-L-Ala-Ac, Ade-2'/3'-D-Ala-Ac, or Ade-2'/3'-Gly-Ac respectively) was confirmed by direct ^{1}H-NMR analysis.

Aminoacyl transfer on tRNA acceptor arm mimics using RNA mixed anhydrides with free amino acids (Gly-pACGCA, or L-Ala-pAGCGA, or D-Ala-pAGCGA, or Ac-L&D-pAGCGA)

The above synthesized RNA mixed anhydride with *N*-tritylalanine or *N*-4-monomethoxytritylglycine (Trt-Ala-pAGCGA or MMTr-Gly-pAGCGA, 2 mM in NH_4OAc buffer, 50 mM, pH = 4.5, 6 μL) was mixed with formic acid (0.2% aq., 6 μL). The mixture was incubated at 20°C for 20 min for MMTr-Gly-pAGCGA or 1 hr for Trt-L-Ala-pAGCGA (or Trt-D-Ala-pAGCGA), respectively. Afterwards, 12 μL 1 M NaCl aq., 12 μL 50 mM $MgCl_2$ aq., 12 μL 0.5 M HEPES aq. (pH = 7.3), 12 μL 1 mM acyl acceptor RNA and ddH_2O were added to a final volume of 120 μL. The mixture was incubated at 10 °C. Aliquots (15 μL) were taken at specific time points, quenched with 1 μL of 0.4 mM HCl solution, and kept in liquid nitrogen before HPLC analysis. HPLC was monitored at 260 nm UV detection (Atlantis™ T3, 3 μm, 4.6 x 150 mm column; flow rate 1 mL/min; LC solvents: A, 10 mM triethylammonium acetate, pH 4.8 in water and B, acetonitrile. Column compartment temperature at 25°C)

Calculation of the corrected yields: Because of the incomplete coupling chemistry and/or background hydrolysis of the alanyl- (or glycyl-) phosphate mixed anhydride donor strand, the yields of aminoacyl-transfer product directly determined by integration of HPLC peaks did not correspond to the yields that would have been determined had the mixed anhydride donor strand been pure. From the amount of the 5'-phosphorylated donor strand at the initial time point and the composite decay curve of the peak for the mixed anhydride thereof (by hydrolysis and transfer), it was possible to use nonlinear regression analysis to infer the relative amounts of these two materials at time zero. The yield of aminoacyl-transfer product observed by HPLC could then be scaled to a corrected yield based upon the calculated amount of mixed anhydride at time zero.

Nicked duplex transfer of an aminoacyl group using RNA mixed anhydrides with free amino acid (Gly-pACGCA, or L-Ala- pAGCGA, or D-Ala-pAGCGA)

The procedure followed is the same as that described above except for the inclusion of 1 equivalent of a third RNA strand (5'-UGGCA-3') as the new aminoacyl acceptor RNA.

(*N*-acetyl)-aminoacyl transfer on tRNA acceptor arm mimics using RNA mixed anhydrides with *N*-acetyl amino acids (Ac-Gly-pACGCA, or Ac-L-Ala-pAGCGA, or Ac-D-Ala-pAGCGA, or Ac-L&D-Ala-pAGCGA)

A mixture containing the above synthesized RNA mixed anhydride with *N*-acetyl amino acid (100 µM, Ac-Gly-pAGCGA, or Ac-L&D-Ala-pAGCGA, or Ac-L-Ala-pAGCGA, or Ac-D-Ala-pAGCGA), acyl acceptor RNA (100 µM), NaCl (1 M) in HEPES buffer (100 mM, pH 7.5) was incubated at 20 °C. Aliquots (10 µL) were taken at specific time points and injected directly to an HPLC for analysis at 260 nm UV detection (Atlantis™ T3, 5 µm, 4.6 x 250 mm column; flow rate 1 mL/min; LC solvents: A, 25 mM triethylammonium acetate, pH 4.5 in water and B, acetonitrile. Column compartment temperature was 25°C).

Calculation of the corrected yields: Because the RNA mixed anhydride with (*N*-acetyl)-aminoacyl were fairly stable during freezing storage at -32°C, transfer yields were corrected simply on the basis of the measured amounts of 5'-p-AGCGA and 5'-Ac-Gly/Ala-p-AGCGA in the starting samples without donor strand.

RNase digestion experiments to confirm that the aminoacyl or *N*-acetylaminoacyl group transfer is to the 2'/3'-diol of the acceptor RNA

An aliquot (9 µL) of the above acyl transfer reaction mixture was added to 1 µL of quenching buffer (NaAc/HAc, 0.5 M, pH 4.0), then 0.2 µL of RNase A solution (10 mg/mL, Thermo Fischer, catalog number: EN0531) was added. The resulting solution was incubated for 30 min at 20 °C then 10 µL of methanol was added and the mixture was centrifuged. The supernatant was analyzed by HPLC using 260 nm UV detection and comparison to chromatograms of synthetic standards of Ade-2'/3'-L-Ala, Ade-2'/3'-D-Ala, Ade-2'/3'-L-Ala-Ac or Ade-2'/3'-D-Ala-Ac, respectively.

Stereoselectivity of *N*-acetylalanyl transfer on tRNA acceptor arm mimics

Three reactions were run in parallel as described above, one with Ac-L-Ala-pAGCGA, one with Ac-D-Ala-pAGCGA and a third with an equal amount of 1:1 Ac-L-Ala-pAGCGA and Ac-D-Ala-pAGCGA as acyl donors respectively. After incubation for a certain time, an aliquot (9 µL) of the above acyl transfer reaction mixture was added to 1 µL of quenching buffer (NaAc/HAc, 0.5 M, pH 4.0), then 0.2 µL of RNase A solution (10 mg/mL, Thermo Fischer, catalog number: EN0531) was added. The resulting solution was incubated for 30 min at 20 °C then 10 µL of methanol was added and the mixture was centrifuged. The supernatant was analyzed by HPLC using 260 nm UV detection (Atlantis™ T3, 5 µm, 4.6 x 250 mm column; flow rate 1 mL/min; LC solvents: A, 25 mM triethylammonium acetate, pH 4.5 in water and B, acetonitrile. Column compartment temperature at 25 °C) and comparison to chromatograms

of synthetic standards of Ade-2'/3'-L-Ala, Ade-2'/3'-D-Ala, Ade-2'/3'-L-Ala-Ac or Ade-2'/3'-D-Ala-Ac, respectively.

HPLC quantification and kinetic regression analysis

The peak areas of mixed anhydride donor strand, hydrolysed mixed anhydride, acceptor strand and diol ester product were determined at different time points. The half-life and the inferred percentage of mixed anhydride at time zero were calculated by nonlinear regression using a one-phase exponential decay, e.g., $ln(P_{mixed\ anhydride}) = -kt + b$, where t refers to the time. Nonlinear regression using a two-phase exponential association decay, e.g. $Yield = A\left(e^{-k_1 t} - e^{-k_2 t}\right)$ was used to calculated k_{transfer} (k_2) and $k_{\text{hydrolysis}}$ (k_1). The half-life of the diol ester was calculated by $t_{1/2} = 0.69 / k_1$. Baseline, determined by the HPLC trace after at least three half-lives, was deducted from the peak areas of transfer products before the regression analysis. The time at which the concentration of the diol ester was maximum was calculated as $t_{max} = \frac{1}{(k_2 - k_1) ln\,(k_2/k_1)}$. The calculated maximum yield was the results of maximum yield from the nonlinear regression, e.g. $Yield_{calc.} = A\left(e^{-k_1 t_{peak}} - e^{-k_2 t_{peak}}\right)$, over the percentage of mixed anhydride at time zero.

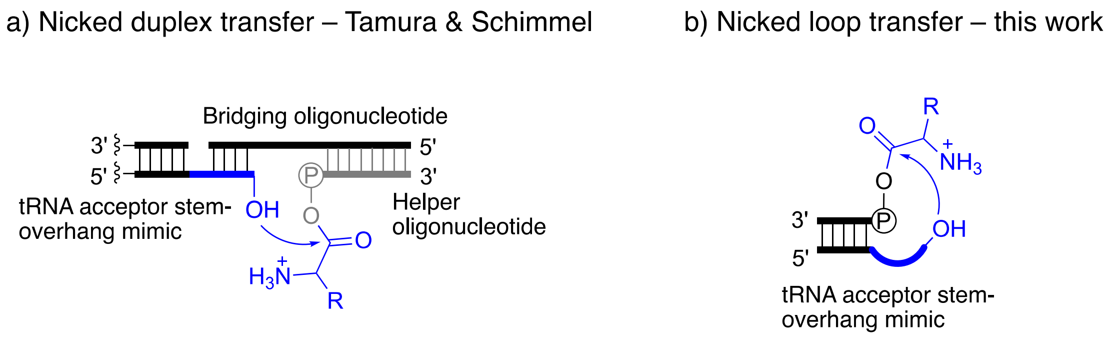

Fig. S1. Nicked duplex and nicked loop aminoacyl-transfer. a) RNA aminoacylation by nicked duplex transfer facilitated by helper and bridging oligonucleotides as described by Tamura & Schimmel *(13,14).* The grey colour indicates a DNA strand. b) RNA aminoacylation by nicked loop transfer in this work in which the RNA stem-overhang mimics the tRNA acceptor stem-overhang.

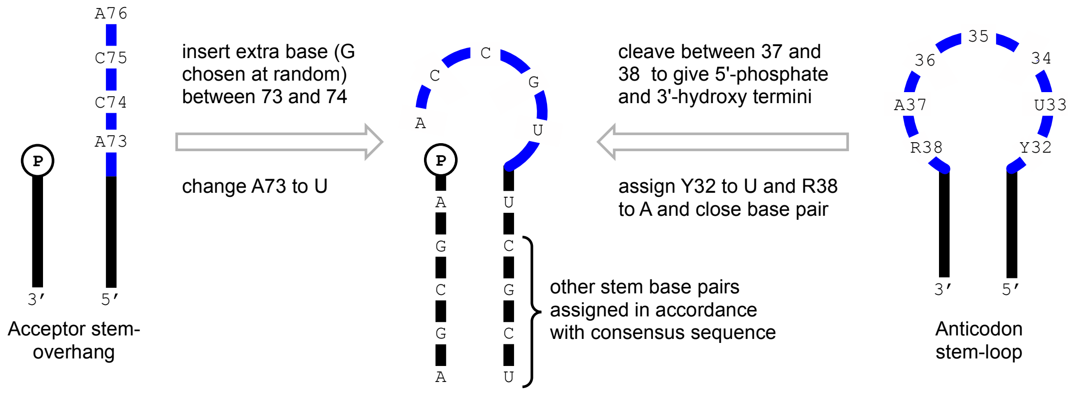

Fig. S2. Reconstruction of a potentially ancient tRNA stem-3'-overhang by a convergent approach. The normal acceptor overhang length of modern tRNAs – 4 nucleotides – is shorter than the anticodon loop length – 7 nucleotides. Accordingly, to converge upon a plausible precursor of both substructures, we lengthened the acceptor overhang by insertion of a nucleotide and shortened the anticodon loop length by closing a base pair between what are now residues 32 and 38. We used consensus sequence data – see *(27)* in particular Fig. 4 and legend – and some structural information to guide our attempted resurrection of an ancient stem-3'-overhang common to both sets of termini of a proto-tRNA dimeric complex. Thus, as U33 in the anticodon loop is responsible for the U-turn of the loop *(28)* and U in place of A73 in the acceptor stem-overhang is known to allow adoption of a folded-back conformation *(12)*, we settled on U as the first nucleotide of the ancestral overhang sequence. Given that the A of the canonical CCA 3'-terminus of the acceptor stem-overhang could correspond with the conserved purine at position 37 of the anticodon loop and the CC would then correspond with the variable anticodon residues 35 and 36 we retained the CCA 3'-terminus in our ancestral reconstruction. This then left us with the choice of the nucleotide to insert into the acceptor overhang before the CCA and we chose G at random. Shifting our attention to the stem region, we made U:A the last base pair because of our decision to close a base pair between residues 32 and 38 and because of the consensus for a pyrimidine at 32 and a purine at 38. If a folded-back 7-nucleotide overhang with a non-Watson-Crick base pair between residues 32 and 38 were instead necessary for aminoacyl-transfer, we reasoned that it would be easier to temporarily access this from a U:A Watson-Crick pair than from a stronger G:C pair.

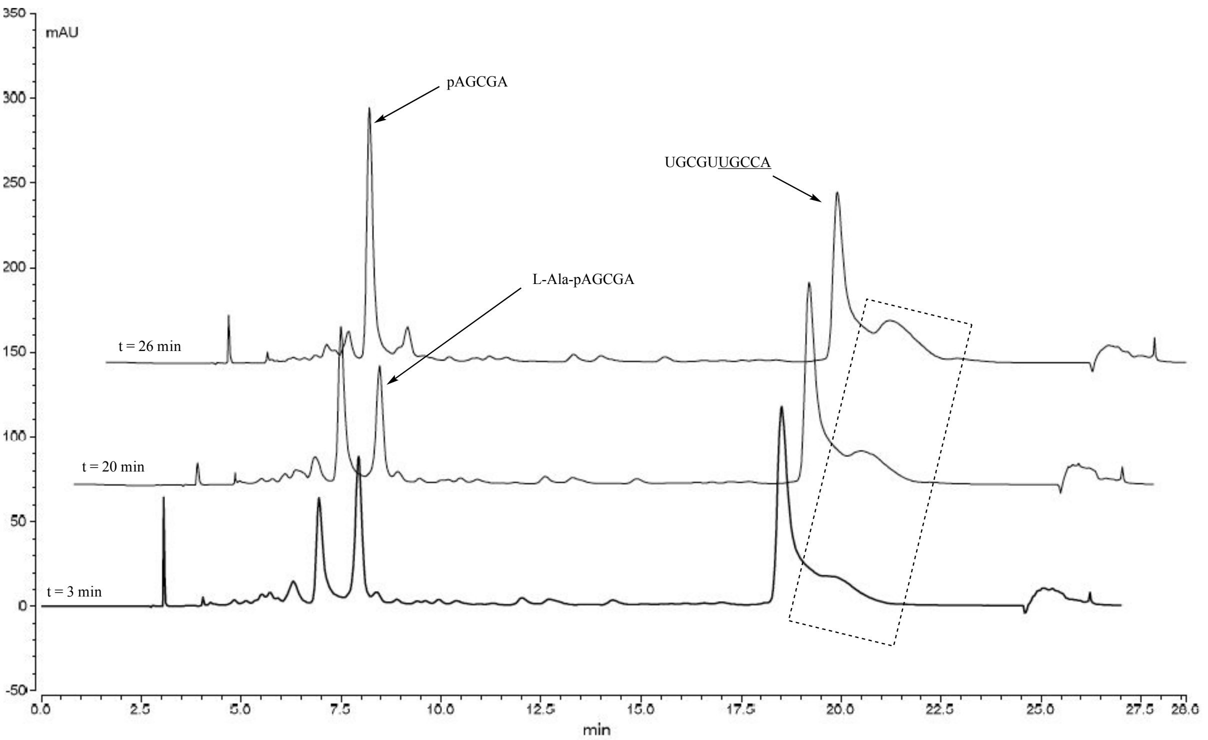

Fig. S3. L-Ala transfer in a tRNA acceptor arm mimic. Loop duplex sequence:

5′UCGCUUGCCA

3′AGCGAp-L-Ala

Transfer was monitored using HPLC with 260 nm UV detection. The solution was incubated at 10°C and aliquots of 8 µL were injected into an HPLC at different time points. Peaks for the donor, the donor mixed anhydride and acceptor strands are indicated. The peak presumed to be due to the diol ester transfer product is highlighted by the dashed box. Conditions: both oligos (100 µM), NaCl (100 mM), $MgCl_2$ (5 mM), HEPES (50 mM, pH 6.8).

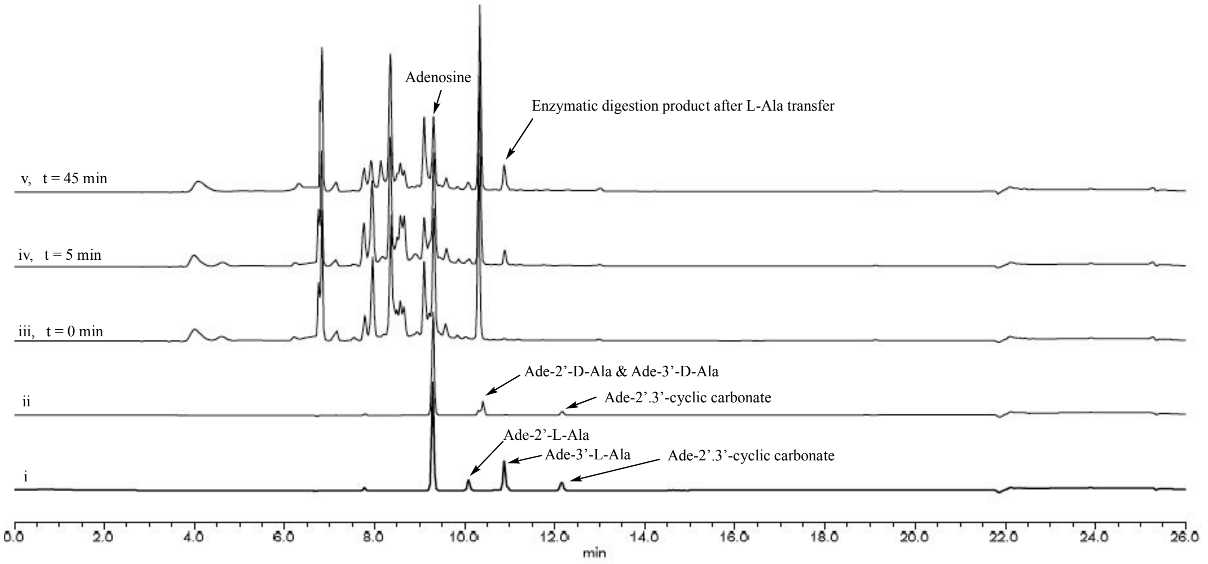

Fig. S4. Enzyme digestion confirming that the acceptor strand 3'-terminal adenosine is aminoacylated by aminoacyl-transfer. A reaction mixture comprising 100 μL L-Ala-pAGCGA (100 μM), UGCGUUGCCA (100 μM), NaCl (100 mM), $MgCl_2$ (5 mM), HEPES (50 mM, pH 6.8) was incubated at 10°C. Then aliquots of 9 μL were added to 1 μL of quenching buffer at the times indicated. 0.2 μL of RNase A (10 mg/mL) was then added to each aliquot and the sample then incubated at 20°C for 30 min. 10 μL of methanol was added, and the resulting mixture was centrifugated. The supernatant was analyzed by HPLC at 260 nm UV detection by comparison with chromatograms of synthetic standards of adenosine-2'/3'-L-Ala and adenosine-2'/3'-D-Ala, respectively. i) Synthetic standards of adenosine-2'/3'-L-Ala, including adenosine-2',3'-cyclic carbonate and adenosine. ii) Synthetic standard of adenosine-2'/3'-D-Ala, including adenosine-2',3'-cyclic carbonate and adenosine. iii-v) Reaction time t = 0 min, 5 min and 45 min, respectively, followed by RNase A digestion.

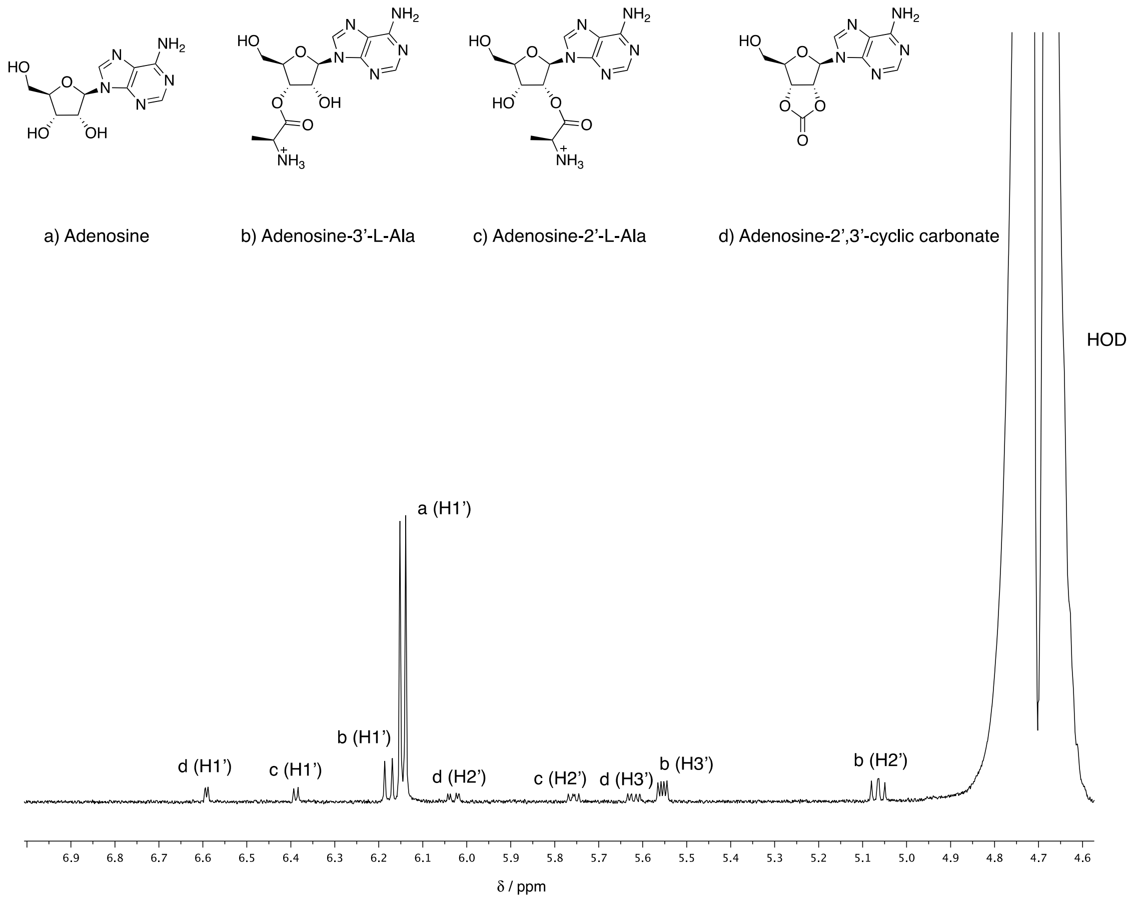

Fig. S5. ^{1}H-NMR spectrum of synthetic standard of L-alanyl diol ester of adenosine (adenosine-3'-L-Ala and adenosine-2'-L-Ala), including adenosine and adenosine-2',3'-cyclic carbonate.

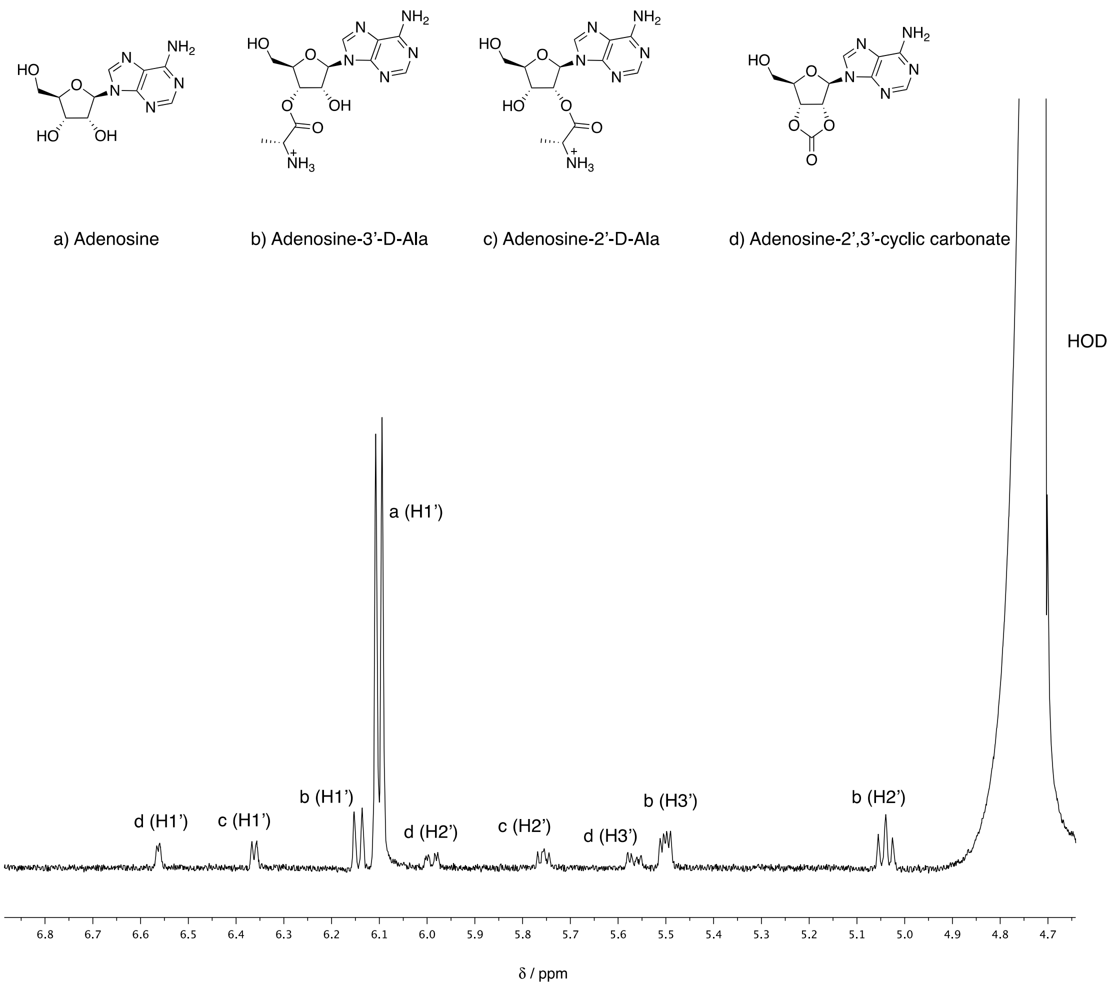

Fig. S6. ^{1}H-NMR spectrum of synthetic standard of D-alanyl diol ester of adenosine (adenosine-3'-D-Ala and adenosine-2'-D-Ala), including adenosine and adenosine-2',3'-cyclic carbonate.

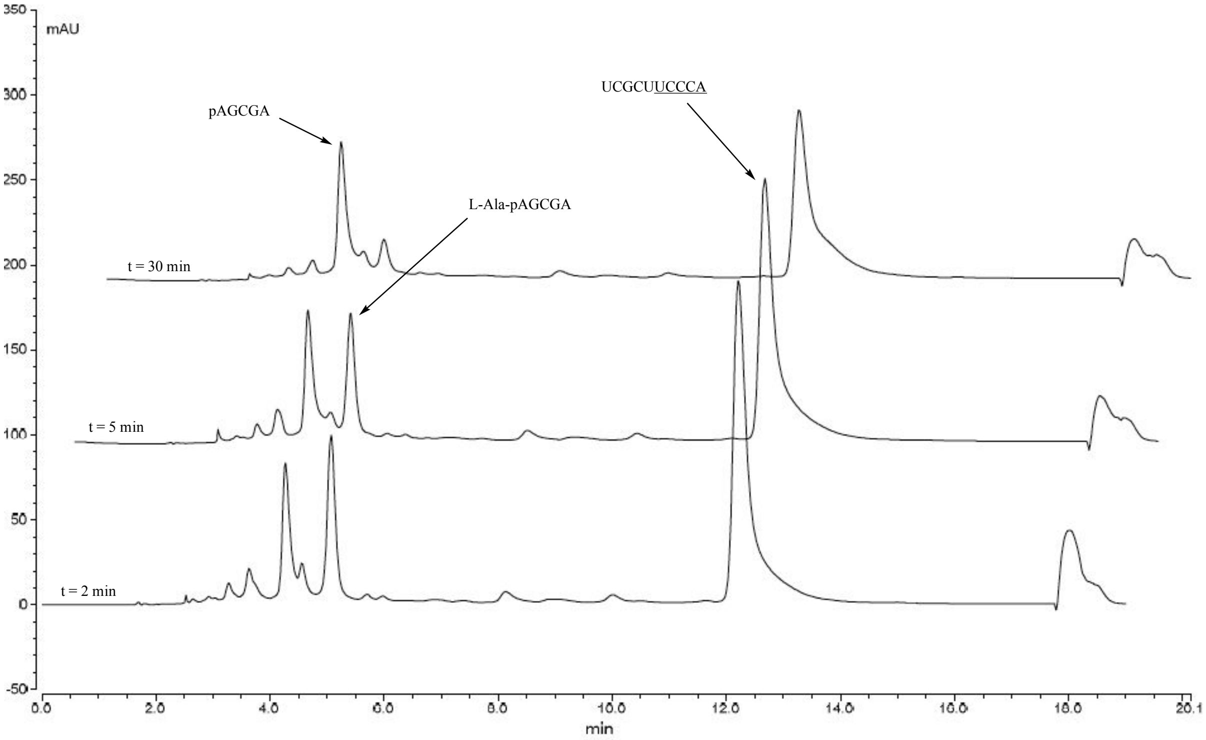

Fig. S7. L-Ala transfer in a tRNA acceptor arm mimic. Loop duplex sequence:

`5′UCGCUUCCCA`

`3′AGCGAp-L-Ala;`

Transfer was monitored using HPLC with 260 nm UV detection. The solution was incubated at 10°C and aliquots of 8 μL were injected into an HPLC at different time points. Peaks for the donor, the donor mixed anhydride and acceptor strands are indicated. Conditions: both oligos (100 μM), NaCl (100 mM), $MgCl_2$ (5 mM), HEPES (50 mM, pH 6.8).

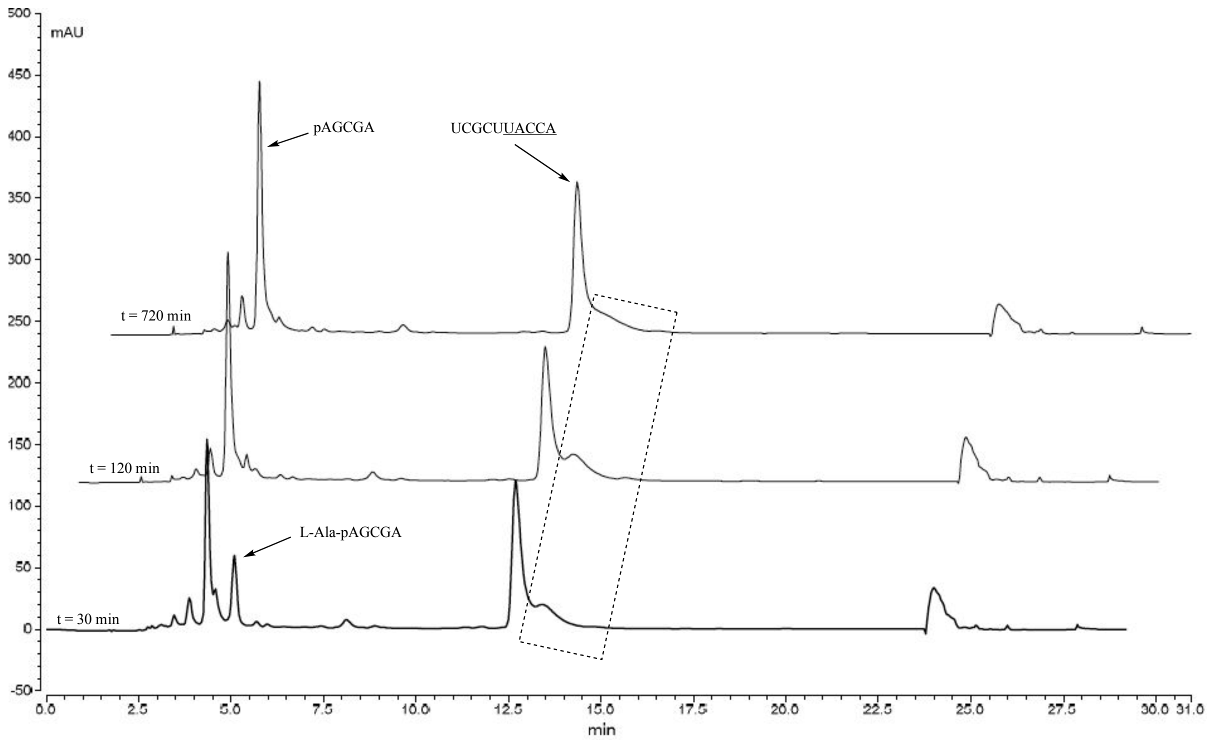

Fig. S8. L-Ala transfer in a tRNA acceptor arm mimic. Loop duplex sequence:

`5′UCGCUUACCA`

`3′AGCGAp-L-Ala;`

Transfer was monitored using HPLC with 260 nm UV detection. The solution was incubated at 10°C and aliquots of 8 μL were injected into an HPLC at different time points. Peaks for the donor, the donor mixed anhydride and acceptor strands are indicated. The peak presumed to be due to the diol ester transfer product is highlighted by the dashed box. Conditions: both oligos (100 μM), NaCl (100 mM), $MgCl_2$ (5 mM), HEPES (50 mM, pH 6.8).

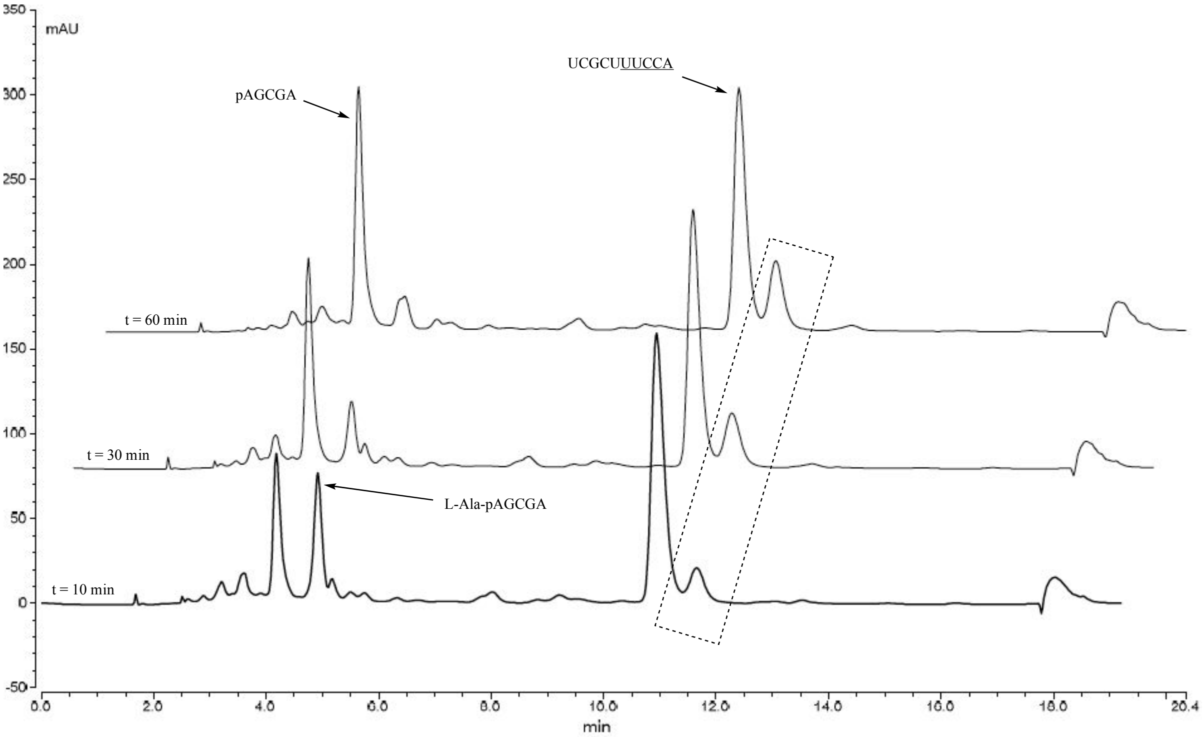

Fig. S9. L-Ala transfer in a tRNA acceptor arm mimic. Loop duplex sequence:

```
5′UCGCUUUCCA
3′AGCGAp-L-Ala;
```

Transfer was monitored using HPLC with 260 nm UV detection. The solution was incubated at 10°C and aliquots of 8 µL were injected into an HPLC at different time points. Peaks for the donor, the donor mixed anhydride and acceptor strands are indicated. The peak presumed to be due to the diol ester transfer product is highlighted by the dashed box. Conditions: both oligos (100 µM), NaCl (100 mM), $MgCl_2$ (5 mM), HEPES (50 mM, pH 6.8).

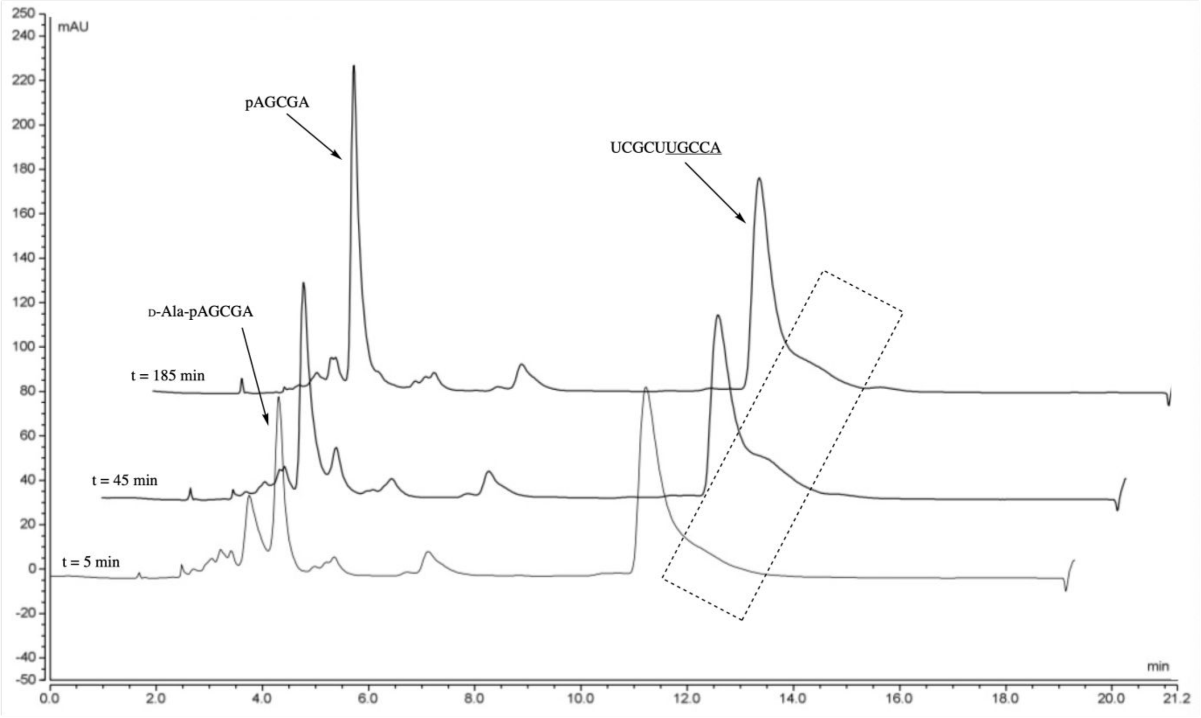

Fig. S10. D-Ala transfer in a tRNA acceptor arm mimic. Loop duplex sequence:

5′UCGCUUGCCA

3′AGCGAp-D-Ala

Transfer was monitored using HPLC with 260 nm UV detection. The solution was incubated at 10°C and aliquots of 8 µL were injected into an HPLC at different time points. Peaks for the donor, the donor mixed anhydride and acceptor strands are indicated. Conditions: both oligos (100 µM), NaCl (100 mM), $MgCl_2$ (5 mM), HEPES (50 mM, pH 6.8).

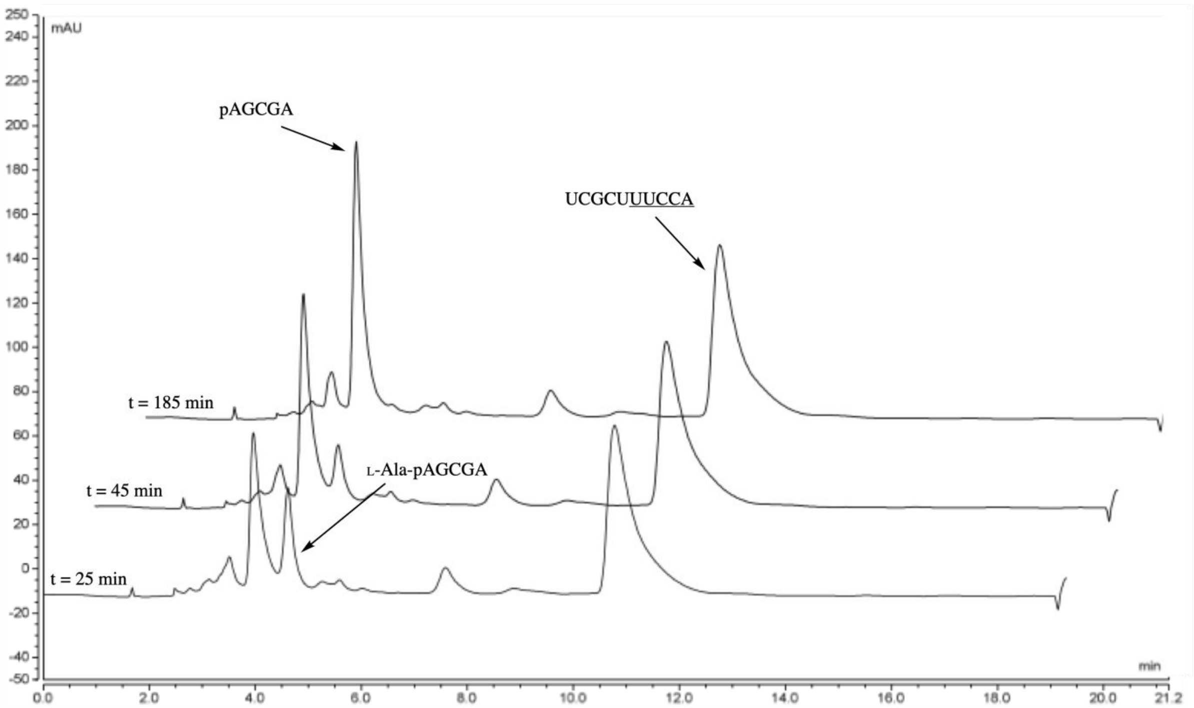

Fig. S11. D-Ala transfer in a tRNA acceptor arm mimic. Loop duplex sequence:

5′UCGCUUUCCA

3′AGCGAp-D-Ala

Transfer was monitored using HPLC with 260 nm UV detection. The solution was incubated at 10°C and aliquots of 8 μL were injected into an HPLC at different time points. Peaks for the donor, the donor mixed anhydride and acceptor strands are indicated. Conditions: both oligos (100 μM), NaCl (100 mM), $MgCl_2$ (5 mM), HEPES (50 mM, pH 6.8).

Fig. S12. Time courses showing the stereoselectivity of L-Ala over D-Ala in nicked loop transfer and nicked duplex transfer.

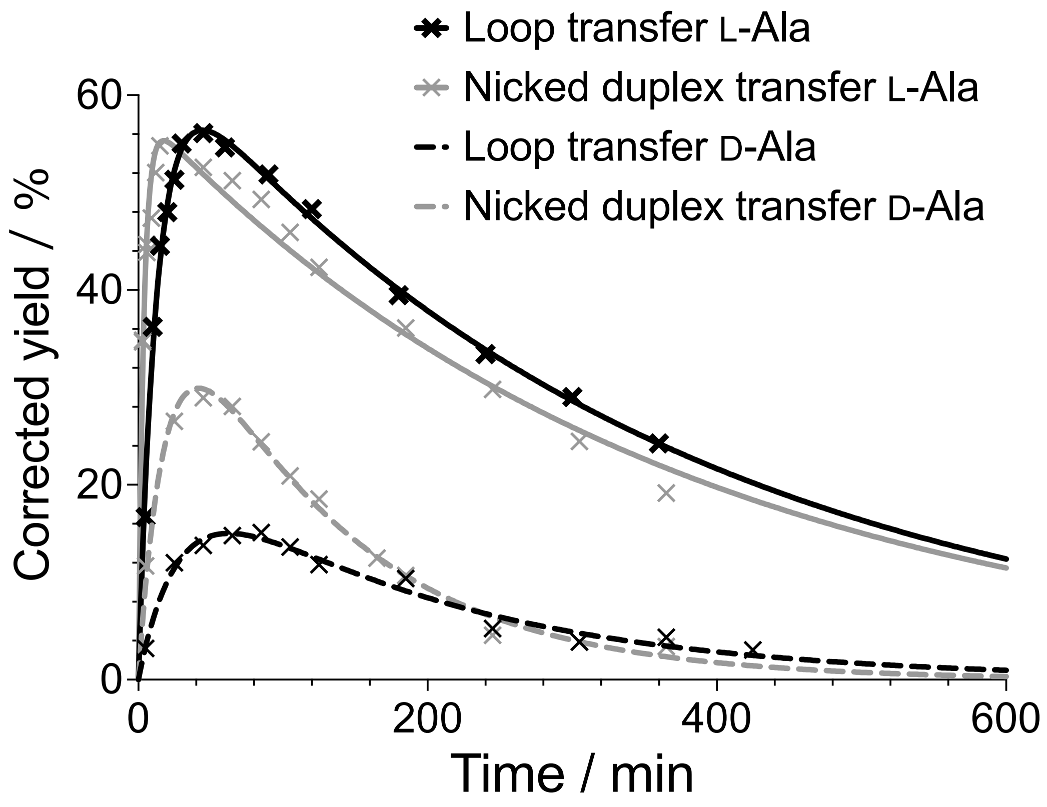

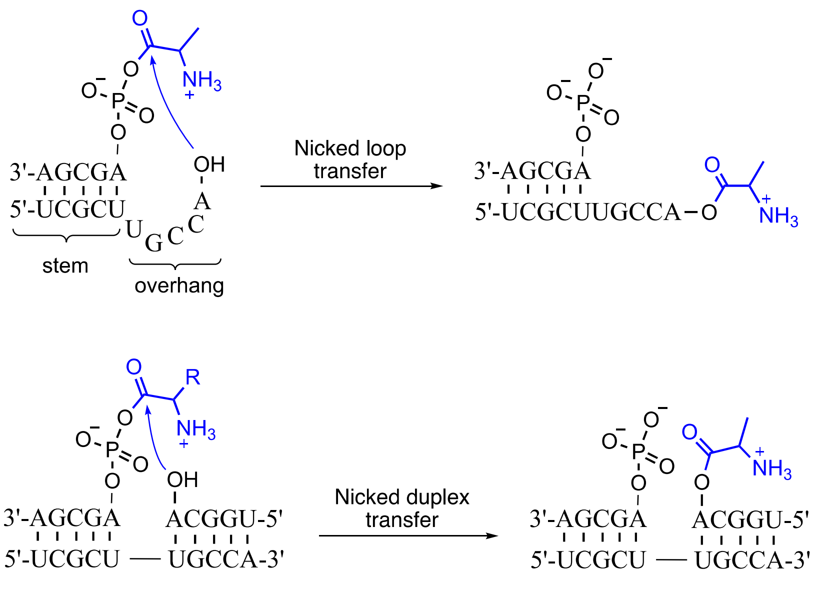

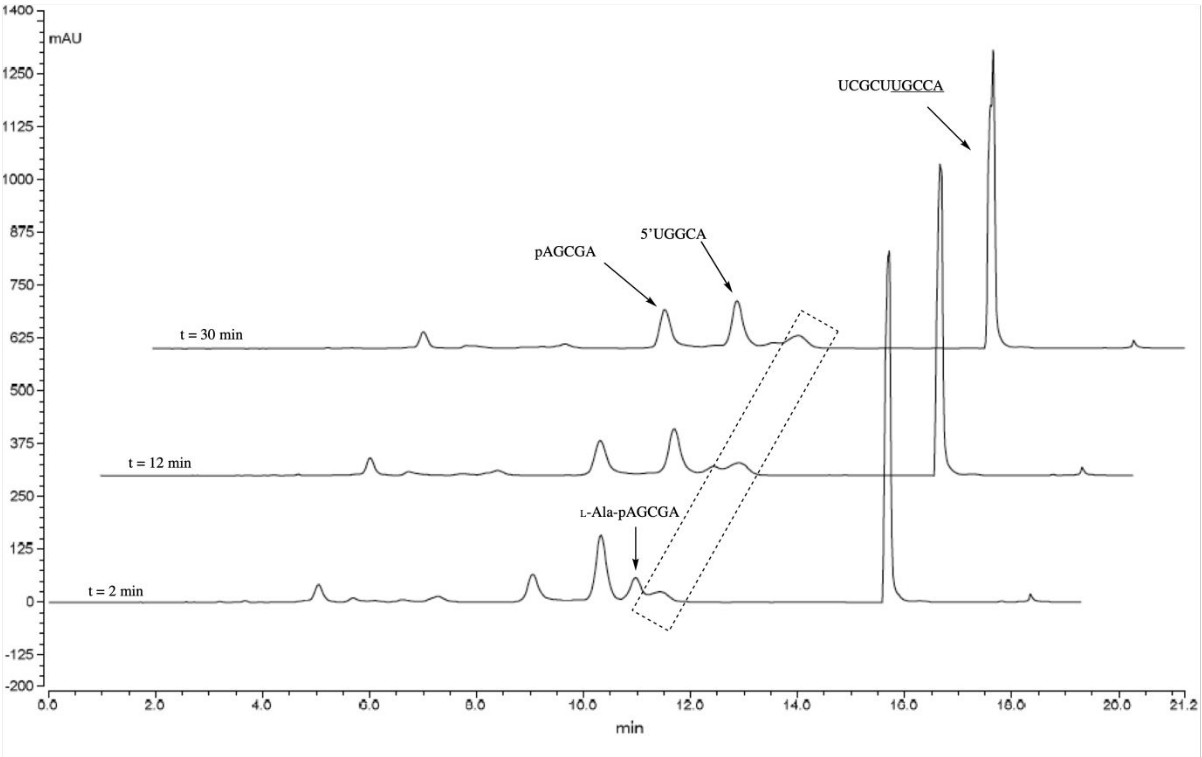

Fig. S13. L-Ala transfer in a nicked RNA duplex. Duplex sequence:

5′UCGCUUGCCA

3′AGCGAp-L-Ala

3′ACGGU

Transfer was monitored using HPLC with 260 nm UV detection. The solution was incubated at 10°C and aliquots of 8 μL were injected into an HPLC at different time points. Peaks for the donor, the donor mixed anhydride and acceptor strands are indicated. The peak presumed to be due to the diol ester transfer product is highlighted by the dashed box. Conditions: each of the three oligos (100 μM), NaCl (100 mM), $MgCl_2$ (5 mM), HEPES (50 mM, pH 6.8).

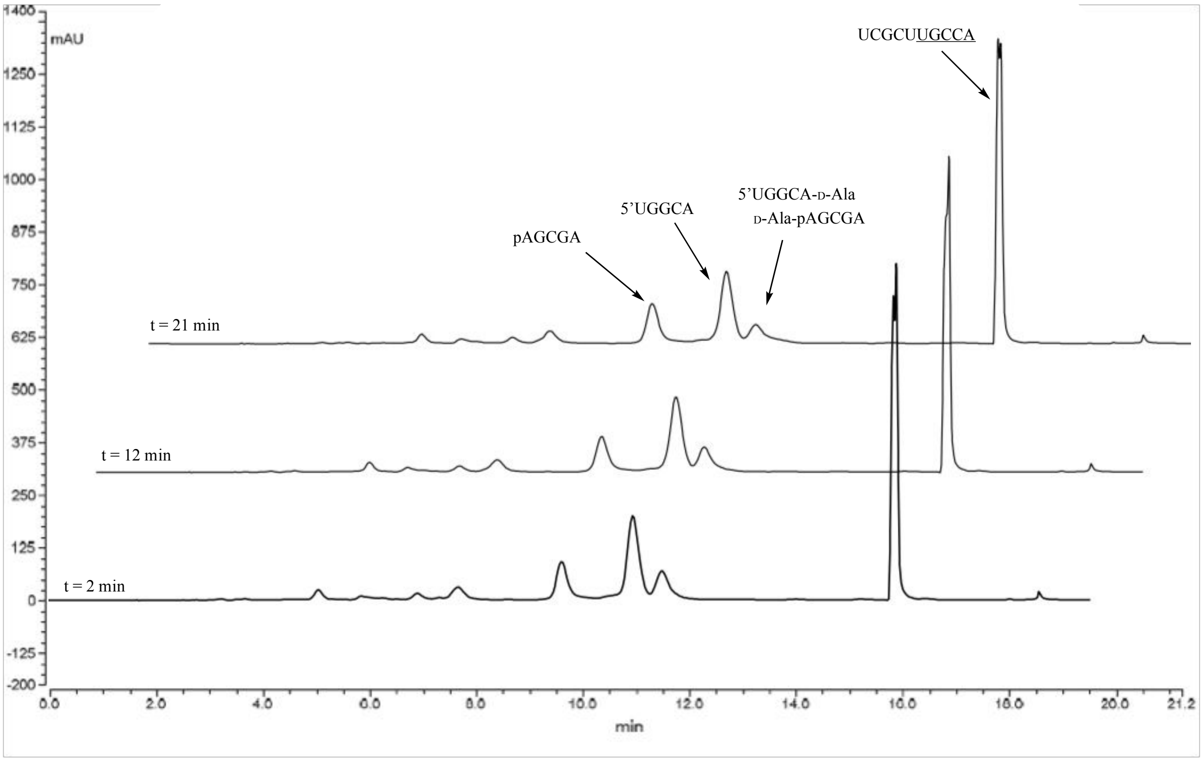

Fig. S14. D-Ala transfer in a nicked RNA duplex. Duplex sequence:

```
5′UCGCUUGCCA
3′AGCGAp-D-Ala
     3′ACGGU
```

Transfer was monitored using HPLC with 260 nm UV detection. The solution was incubated at 10°C and aliquots of 8 µL were injected into an HPLC at different time points. Peaks for the donor, the donor mixed anhydride and acceptor strands are indicated. Conditions: each of the three oligos (100 µM), NaCl (100 mM), $MgCl_2$ (5 mM), HEPES (50 mM, pH 6.8).

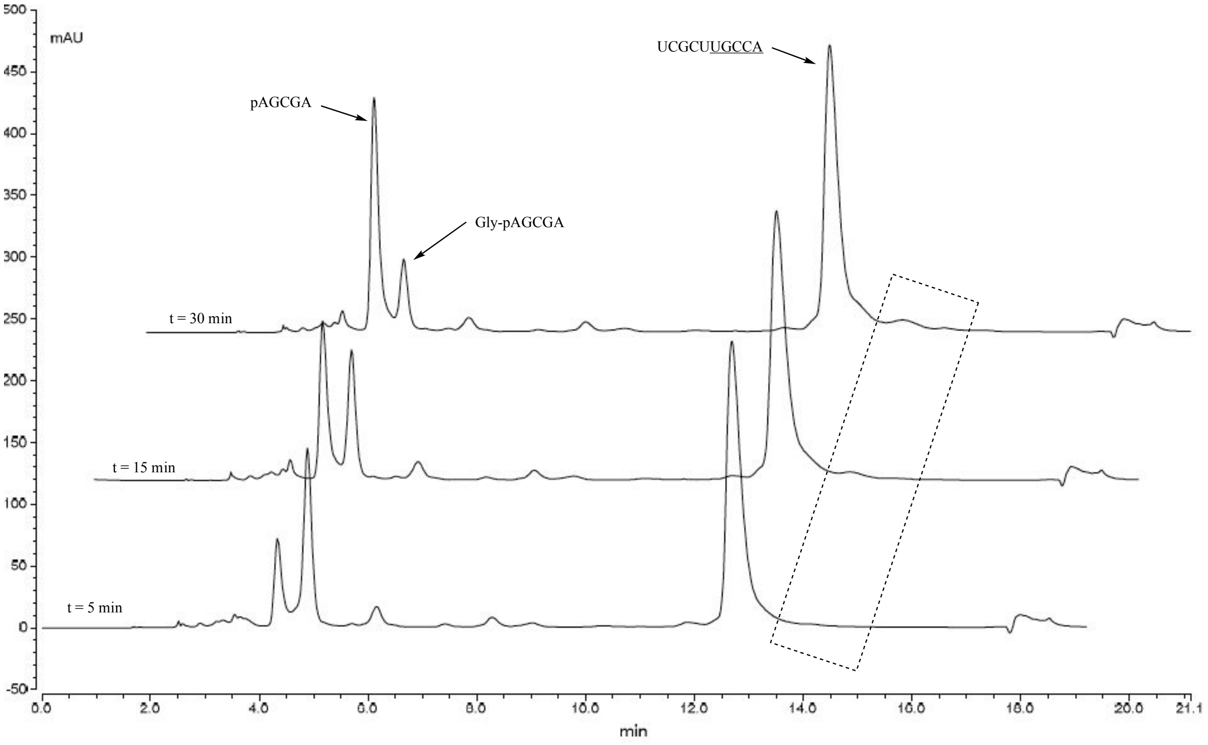

Fig. S15. Gly transfer in a tRNA acceptor arm mimic. Loop duplex sequence:

5′UCGCUUGCCA

3′AGCGAp-Gly

Transfer was monitored using HPLC with 260 nm UV detection. The solution was incubated at 10°C and aliquots of 8 μL were injected into an HPLC at different time points. Peaks for the donor, the donor mixed anhydride and acceptor strands are indicated. The peak presumed to be due to the diol ester transfer product is highlighted by the dashed box. Conditions: both oligos (100 μM), NaCl (100 mM), $MgCl_2$ (5 mM), HEPES (50 mM, pH 6.8).

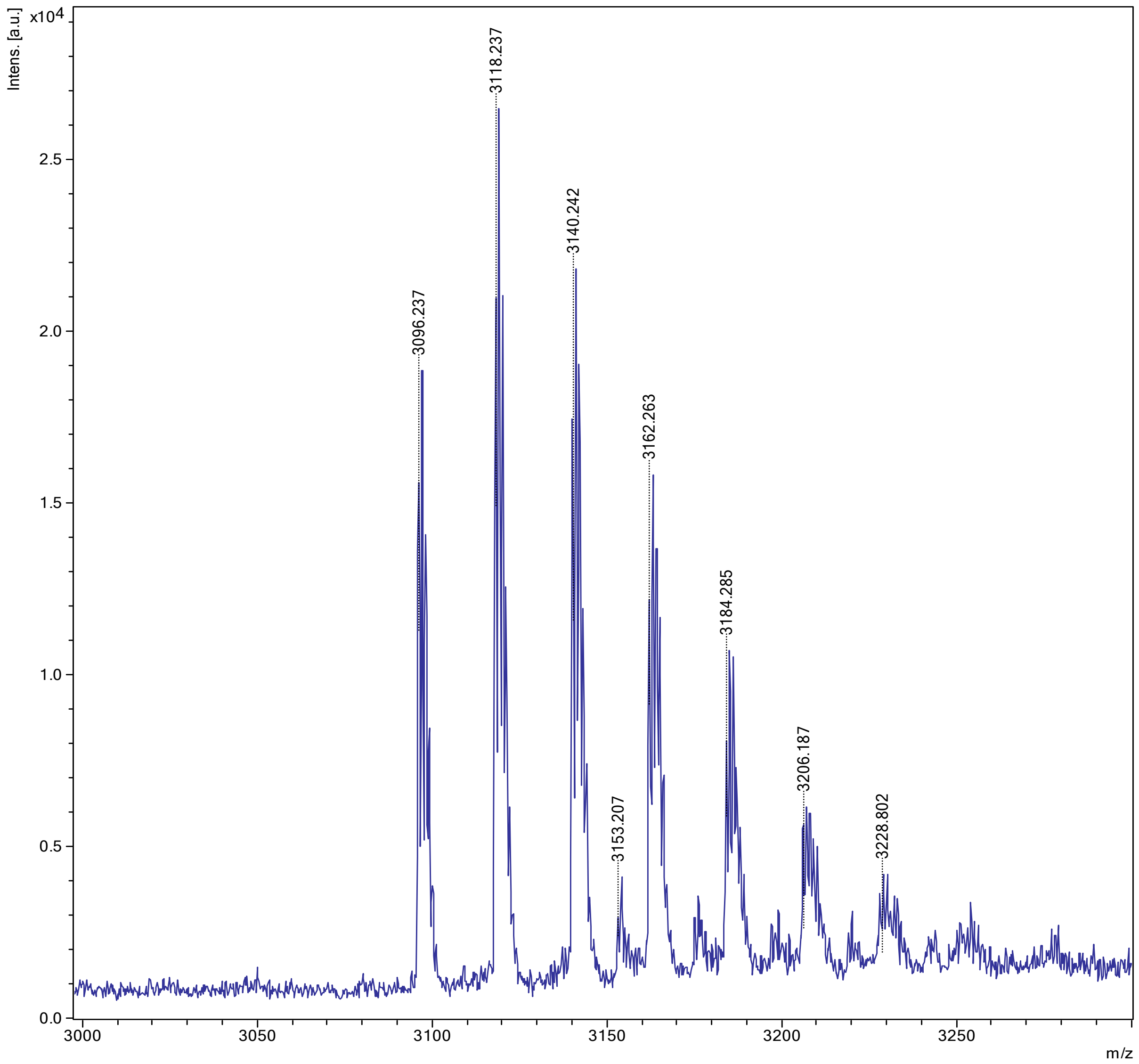

Fig. S16. MALDI-TOF mass spectrum of the reaction products described in Fig. S12a. Mass clusters for the acceptor strand (UCGCUUGCCA, MW = 3096.237 Da) and its L-glycyl diol ester product (UCGCUUGCCA-Gly, MW = 3153.207 Da) are indicated. Measured mass difference, 56.970, C_2H_3NO [Gly-H_2O], calculated 57.022.

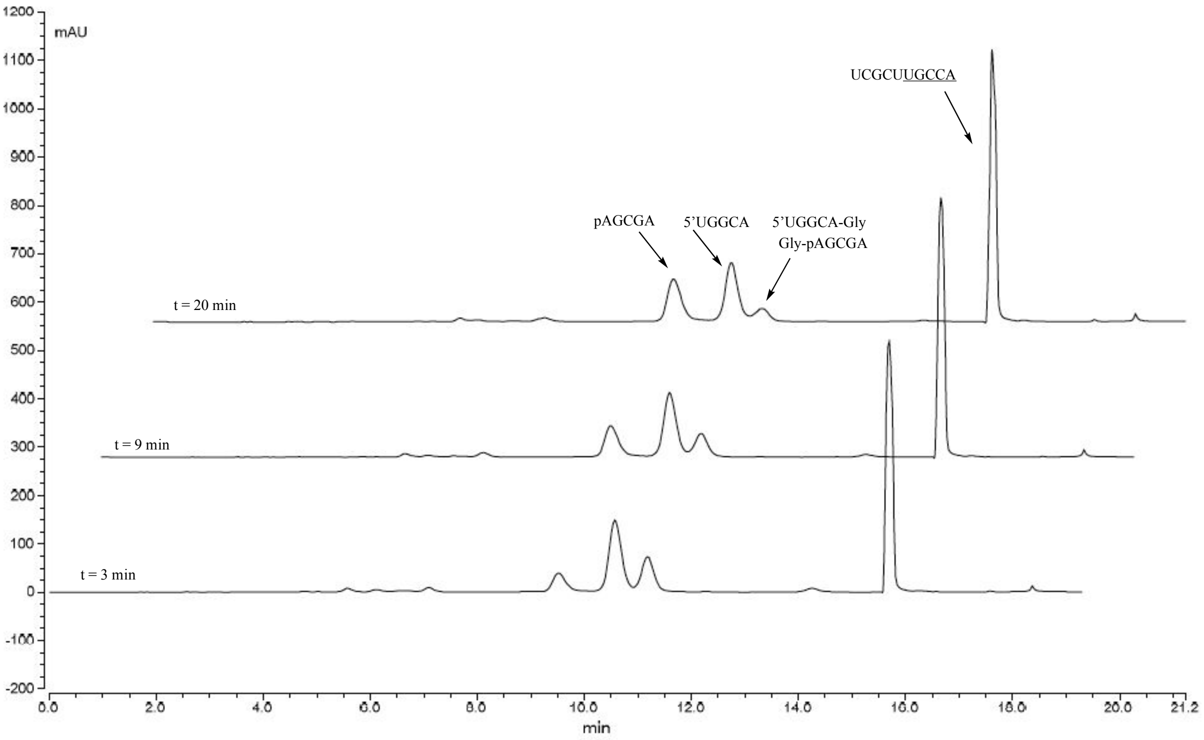

Fig. S17. Gly transfer in a nicked RNA duplex. Duplex sequence:

```
5′UCGCUUGCCA
3′AGCGAp-Gly
     3′ACGGU
```

Transfer was monitored using HPLC with 260 nm UV detection. The solution was incubated at 10°C and aliquots of 8 µL were injected into an HPLC at different time points. Peaks for the donor, the donor mixed anhydride and acceptor strands are indicated. Conditions: each of the three oligos (100 µM), NaCl (100 mM), $MgCl_2$ (5 mM), HEPES (50 mM, pH 6.8).

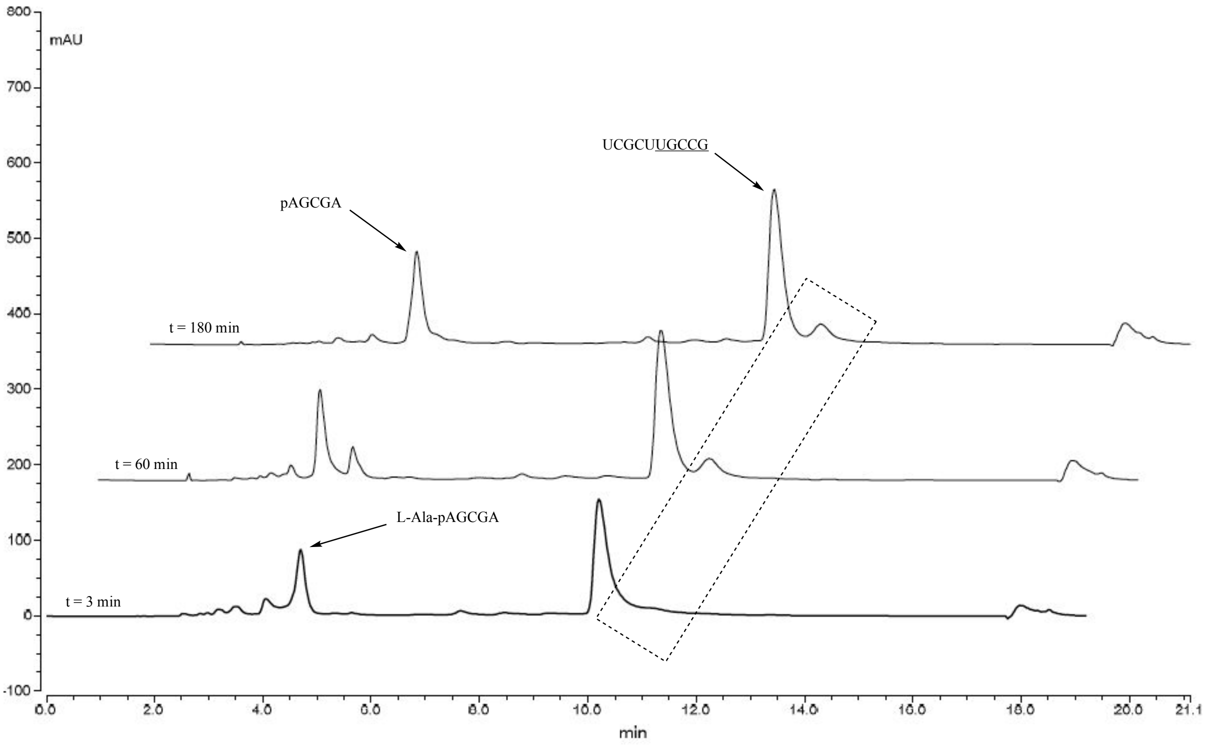

Fig. S18. L-Ala transfer in a tRNA acceptor arm mimic. Loop duplex sequence:

5′UCGCUUGCCG

3′AGCGAp-L-Ala;

Transfer was monitored using HPLC with 260 nm UV detection. The solution was incubated at 10°C and aliquots of 8 μL were injected into an HPLC at different time points. Peaks for the donor, the donor mixed anhydride and acceptor strands are indicated. The peak presumed to be due to the diol ester transfer product is highlighted by the dashed box. Conditions: both oligos (100 μM), NaCl (100 mM), $MgCl_2$ (5 mM), HEPES (50 mM, pH 6.8).

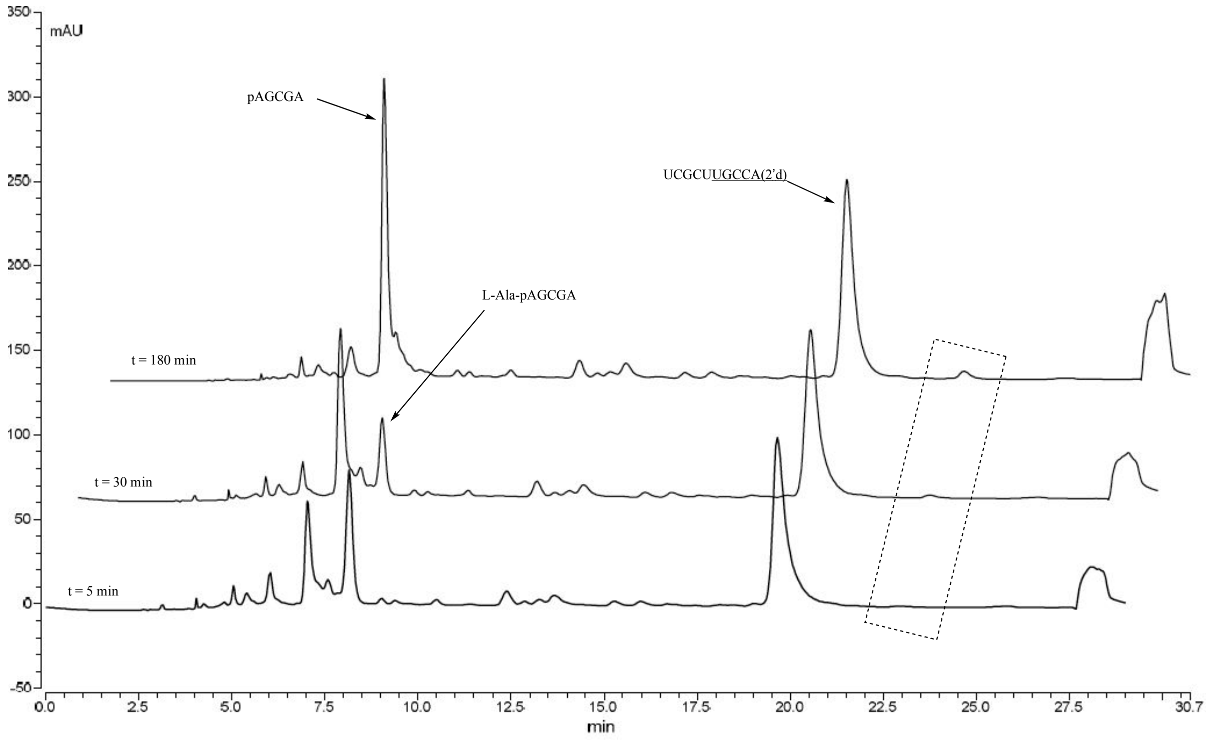

Fig. S19. L-Ala transfer in a tRNA acceptor arm mimic. Loop duplex sequence:

5′UCGCUUGCCA(2′d)

3′AGCGAp-L-Ala

Transfer was monitored using HPLC with 260 nm UV detection. The solution was incubated at 10°C and aliquots of 8 µL were injected into an HPLC at different time points. Peaks for the donor, the donor mixed anhydride and acceptor strands are indicated. The peak presumed to be due to the diol ester transfer product is highlighted by the dashed box. Conditions: both oligos (100 µM), NaCl (100 mM), $MgCl_2$ (5 mM), HEPES (50 mM, pH 6.8).

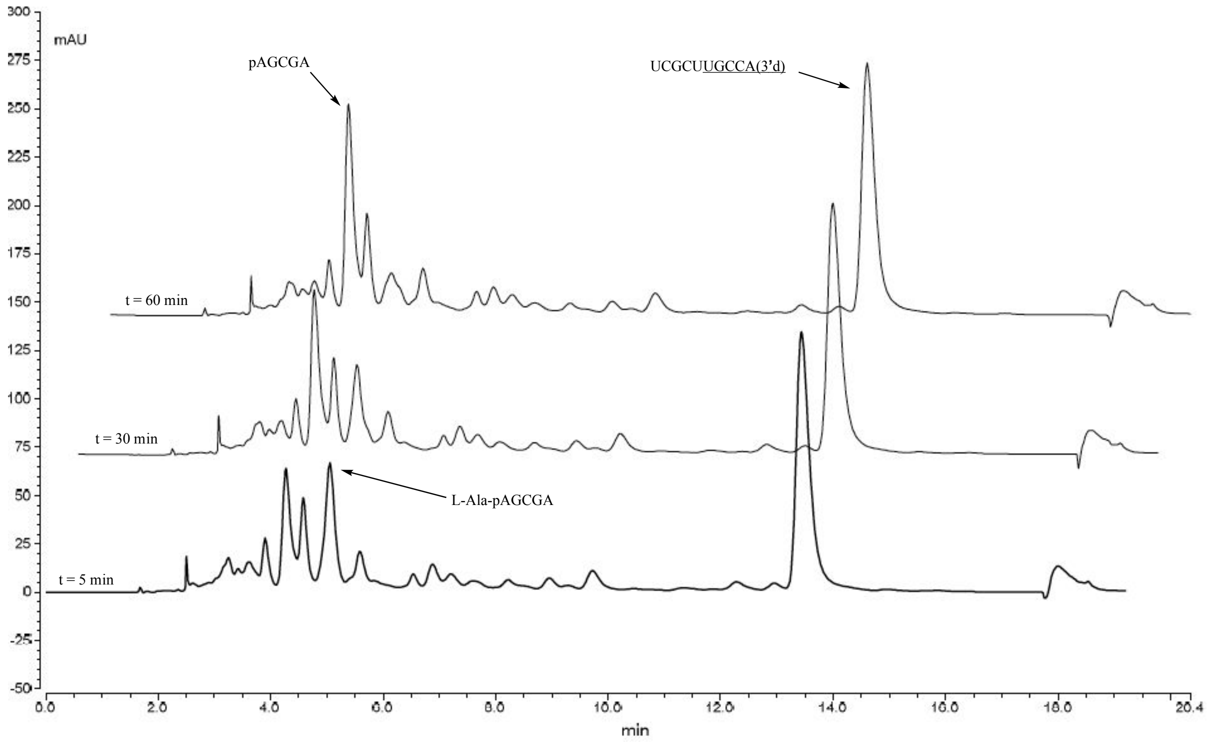

Fig. S20. L-Ala transfer in a tRNA acceptor arm mimic. Loop duplex sequence:

5′UCGCU<u>UGCCA(3′d)</u>

3′AGCGAp-L-Ala

Transfer was monitored using HPLC with 260 nm UV detection. The solution was incubated at 10°C and aliquots of 8 μL were injected into an HPLC at different time points. Peaks for the donor, the donor mixed anhydride and acceptor strands are indicated. Conditions: both oligos (100 μM), NaCl (100 mM), $MgCl_2$ (5 mM), HEPES (50 mM, pH 6.8).

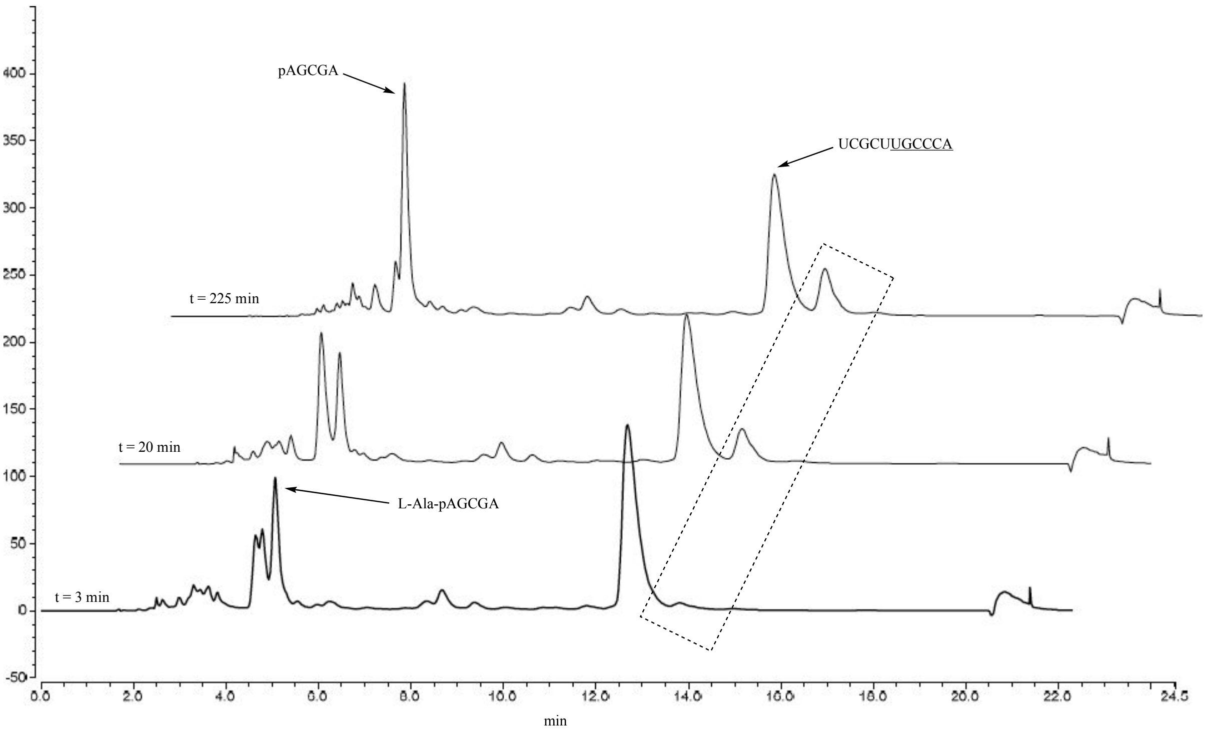

Fig. S21. L-Ala transfer in a tRNA acceptor arm mimic. Loop duplex sequence:

5′UCGCUUGCCCA

3′AGCGAp-L-Ala

Transfer was monitored using HPLC with 260 nm UV detection. The solution was incubated at 10°C and aliquots of 8 µL were injected into an HPLC at different time points. Peaks for the donor, the donor mixed anhydride and acceptor strands are indicated. The peak presumed to be due to the diol ester transfer product is highlighted by the dashed box. Conditions: both oligos (100 µM), NaCl (100 mM), $MgCl_2$ (5 mM), HEPES (50 mM, pH 6.8).

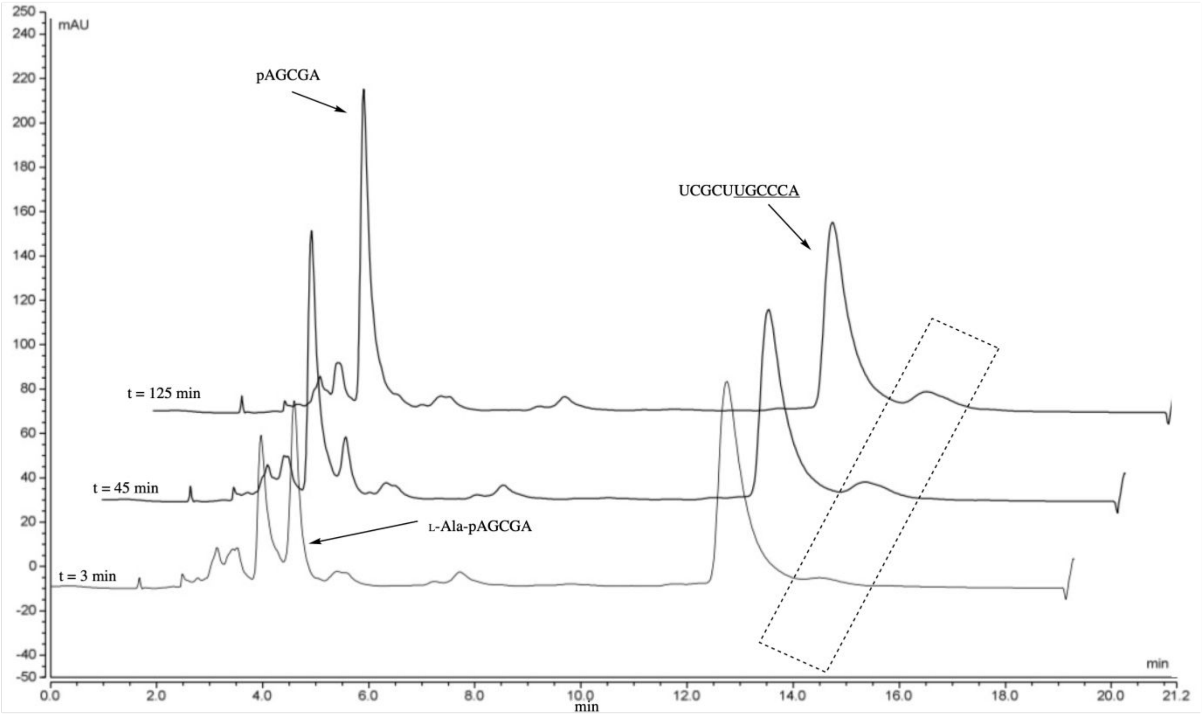

Fig. S22. D-Ala transfer in a tRNA acceptor arm mimic. Loop duplex sequence:

5′UCGCU<u>UGCCCA</u>

3′AGCGAp-D-Ala

Transfer was monitored using HPLC with 260 nm UV detection. The solution was incubated at 10°C and aliquots of 8 μL were injected into an HPLC at different time points. Peaks for the donor, the donor mixed anhydride and acceptor strands are indicated. The peak presumed to be due to the diol ester transfer product is highlighted by the dashed box. Conditions: both oligos (100 μM), NaCl (100 mM), $MgCl_2$ (5 mM), HEPES (50 mM, pH 6.8).

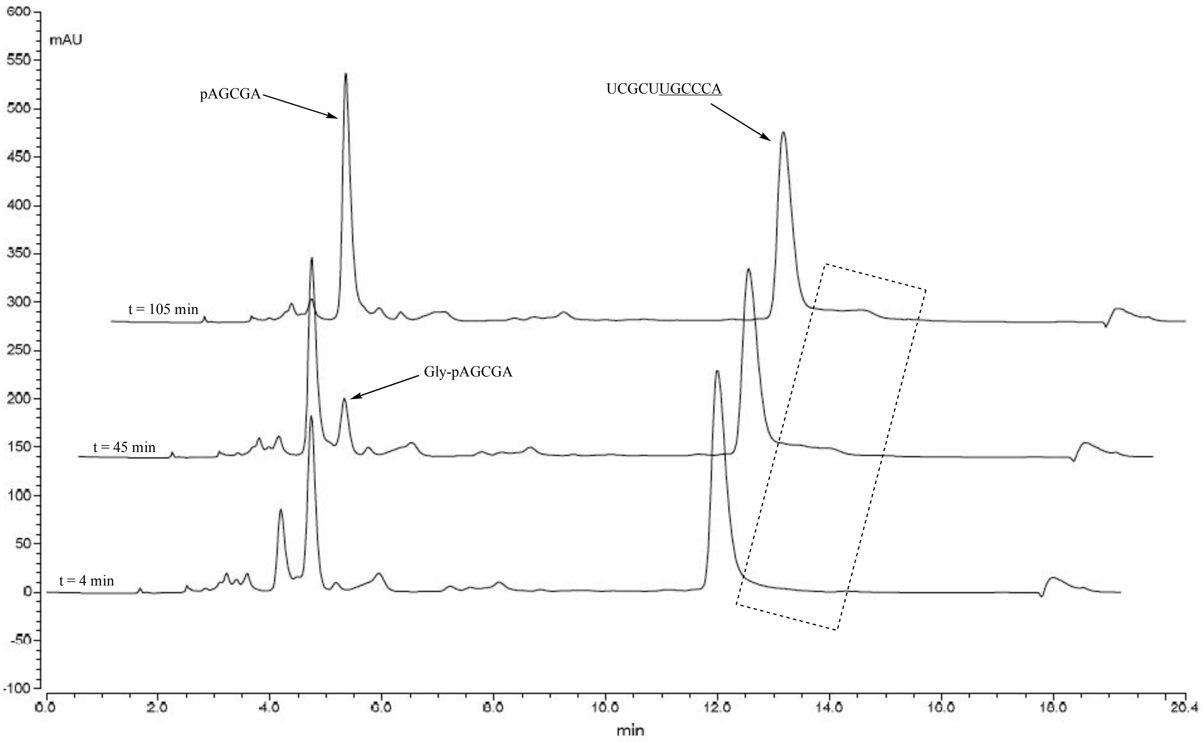

Fig. S23. Gly transfer in a tRNA acceptor arm mimic. Loop duplex sequence:

5′UCGCU<u>UGCCCA</u>

3′AGCGAp-Gly

Transfer was monitored using HPLC with 260 nm UV detection. The solution was incubated at 10°C and aliquots of 8 μL were injected into an HPLC at different time points. Peaks for the donor, the donor mixed anhydride and acceptor strands are indicated. The peak presumed to be due to the diol ester transfer product is highlighted by the dashed box. Conditions: both oligos (100 μM), NaCl (100 mM), $MgCl_2$ (5 mM), HEPES (50 mM, pH 6.8).

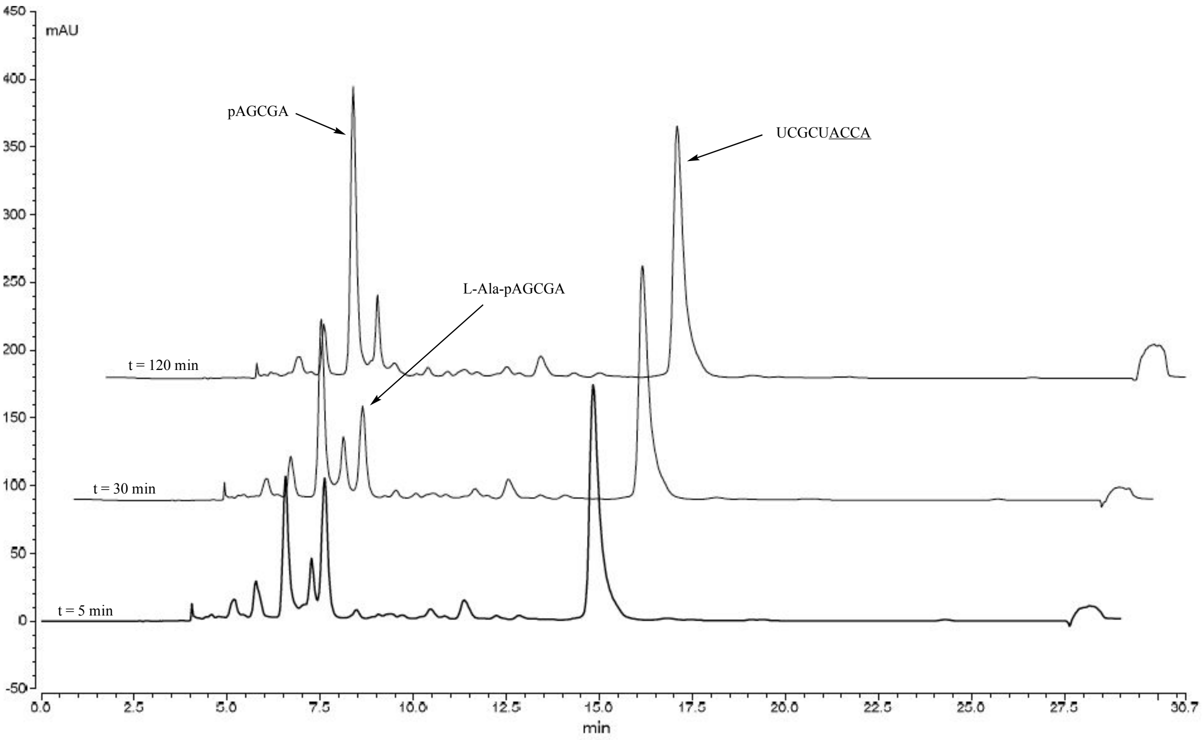

Fig. S24. L-Ala transfer in a tRNA acceptor arm mimic. Loop duplex sequence:

5′UCGCUACCA

3′AGCGAp-L-Ala

Transfer was monitored using HPLC with 260 nm UV detection. The solution was incubated at 10°C and aliquots of 8 μL were injected into an HPLC at different time points. Peaks for the donor, the donor mixed anhydride and acceptor strands are indicated. Conditions: both oligos (100 μM), NaCl (100 mM), $MgCl_2$ (5 mM), HEPES (50 mM, pH 6.8).

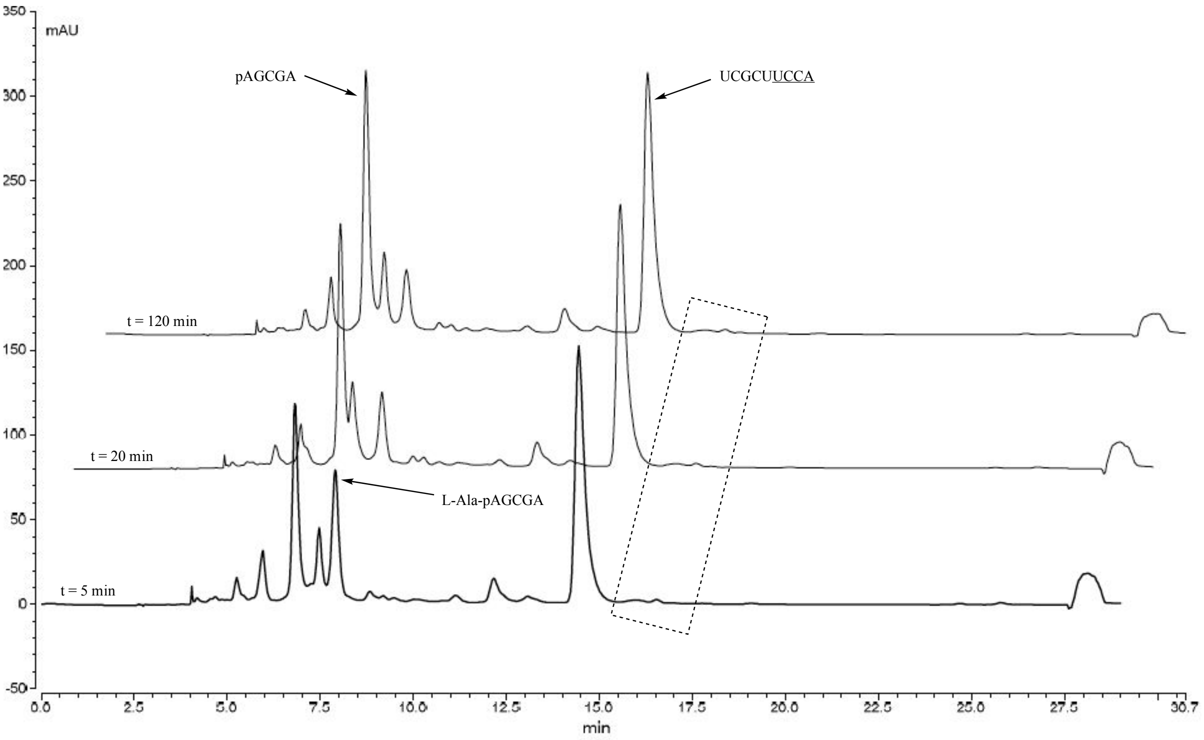

Fig. S25. L-Ala transfer in a tRNA acceptor arm mimic. Loop duplex sequence:

5′UCGCUUCCA

3′AGCGAp-L-Ala

Transfer was monitored using HPLC with 260 nm UV detection. The solution was incubated at 10°C and aliquots of 8 μL were injected into an HPLC at different time points. Peaks for the donor, the donor mixed anhydride and acceptor strands are indicated. The peak presumed to be due to the diol ester transfer product is highlighted by the dashed box. Conditions: both oligos (100 μM), NaCl (100 mM), $MgCl_2$ (5 mM), HEPES (50 mM, pH 6.8).

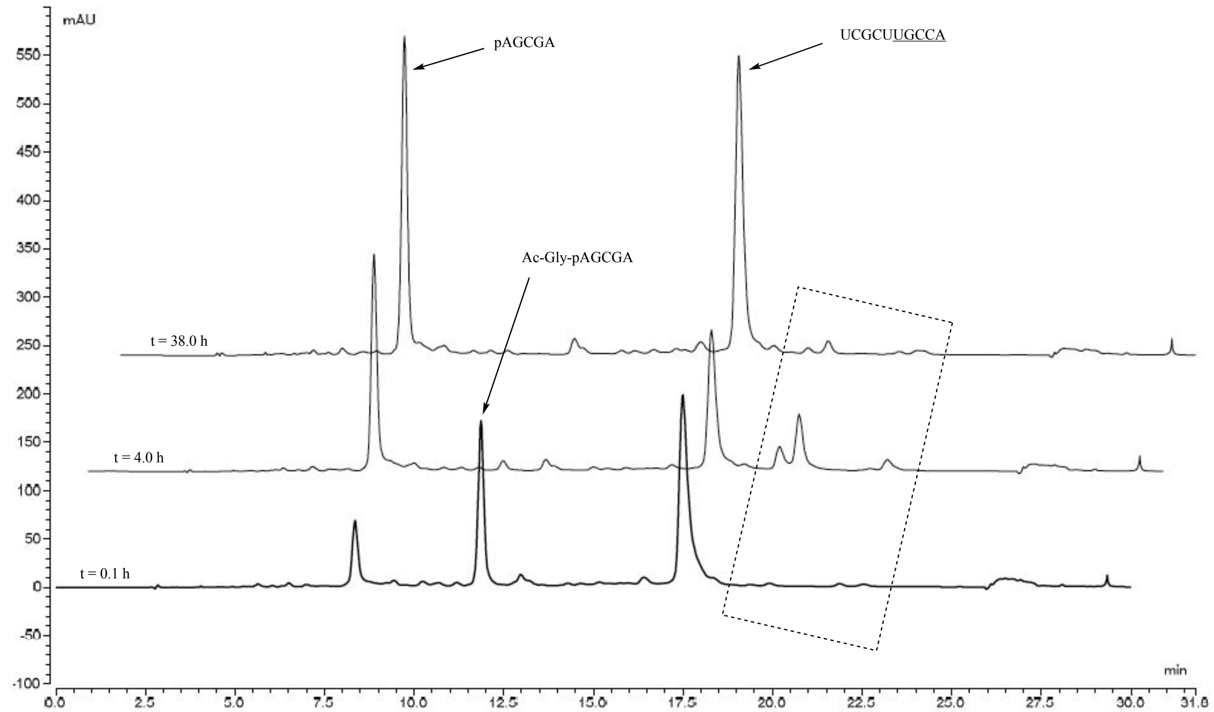

Fig. S26. Ac-Gly transfer in a tRNA acceptor arm mimic. Loop duplex sequence:

`5’UCGCU`<u>`UGCCA`</u>

`3’AGCGAp-Gly-Ac`

Transfer was monitored using HPLC with 260 nm UV detection. The solution was incubated at 20°C and aliquots of 8 μL were injected into an HPLC at different time points. Peaks for the donor, the donor mixed anhydride and acceptor strands are indicated. The peak presumed to be due to the diol ester transfer product is highlighted by the dashed box. Conditions: both oligos (100 μM), NaCl (1000 mM), HEPES (100 mM, pH 7.5).

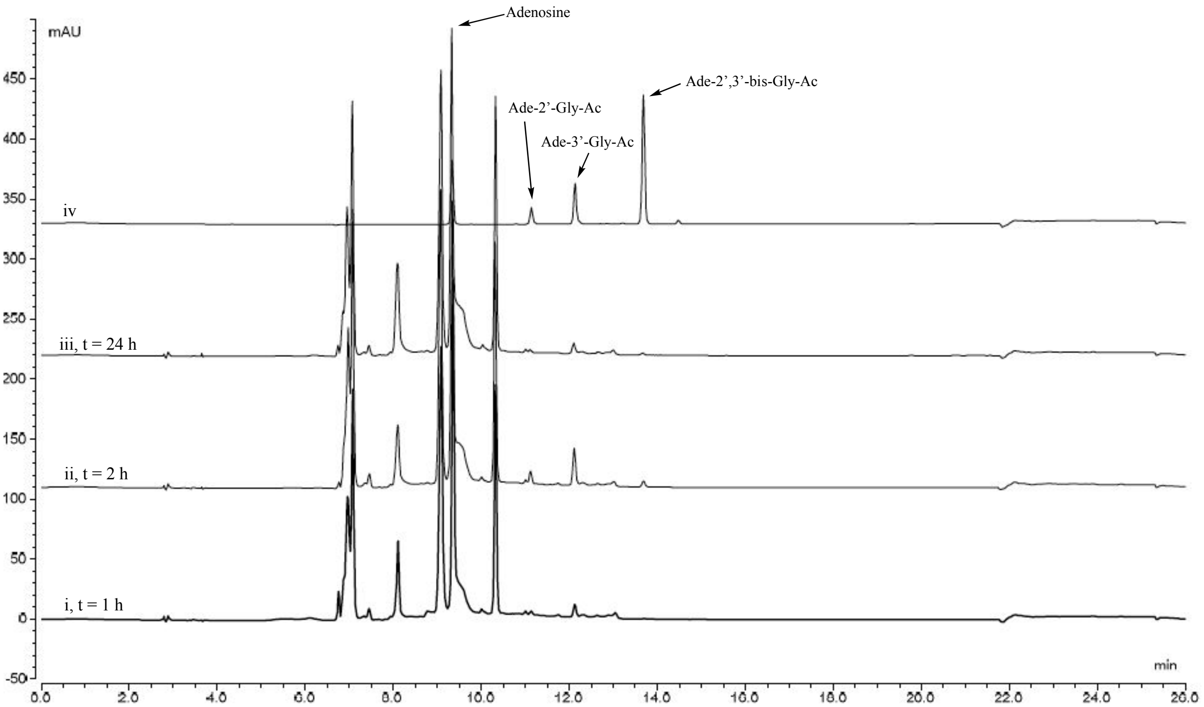

Fig. S27. Enzyme digestion experiment to confirm that Ac-Gly is transferred to the diol of the 3'-end in a tRNA acceptor arm mimic with overhanging sequence UGCCA. A reaction mixture comprising 100 µL with Ac-Gly-pAGCGA (100 µM), UGCGUUGCCA (100 µM), NaCl (1 M), HEPES (100 mM) at pH 7.5 was incubated at 20 °C. Then aliquots of 9 µL were added to 1 µL of quenching buffer at time i) t = 1 h, ii) t = 3 h, iii) t = 24 h. 0.2 µL of Rnase A (10 mg/mL) was then added to each aliquot, and the samples were incubated at 20°C for 30 min. 10 µL of methanol was added, and the resulting mixture was centrifugated. The supernatant was analyzed by HPLC at 260 nm UV detection and comparison to chromatograms of synthetic standards of Ade-2'/3'-Gly-Ac. iv) synthetic standard of Ade-2'/3'-Gly-Ac, including Ade-2',3'-bis-Gly-Ac and adenosine.

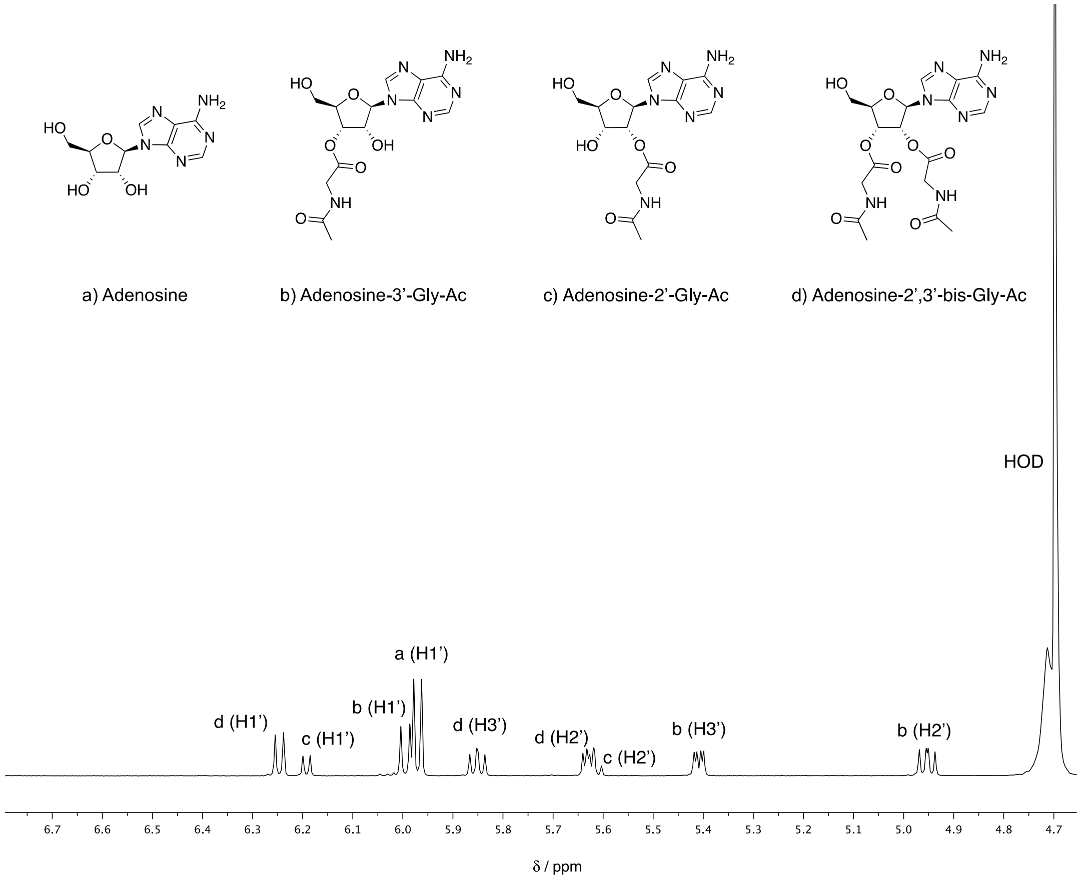

Fig. S28. ^{1}H-NMR spectrum of synthetic standard of *N*-acetyl-glycyl diol ester of adenosine (adenosine-3'-Gly-Ac and adenosine-2'-Gly-Ac), including adenosine and adenosine-2',3'-bis-Gly-Ac.

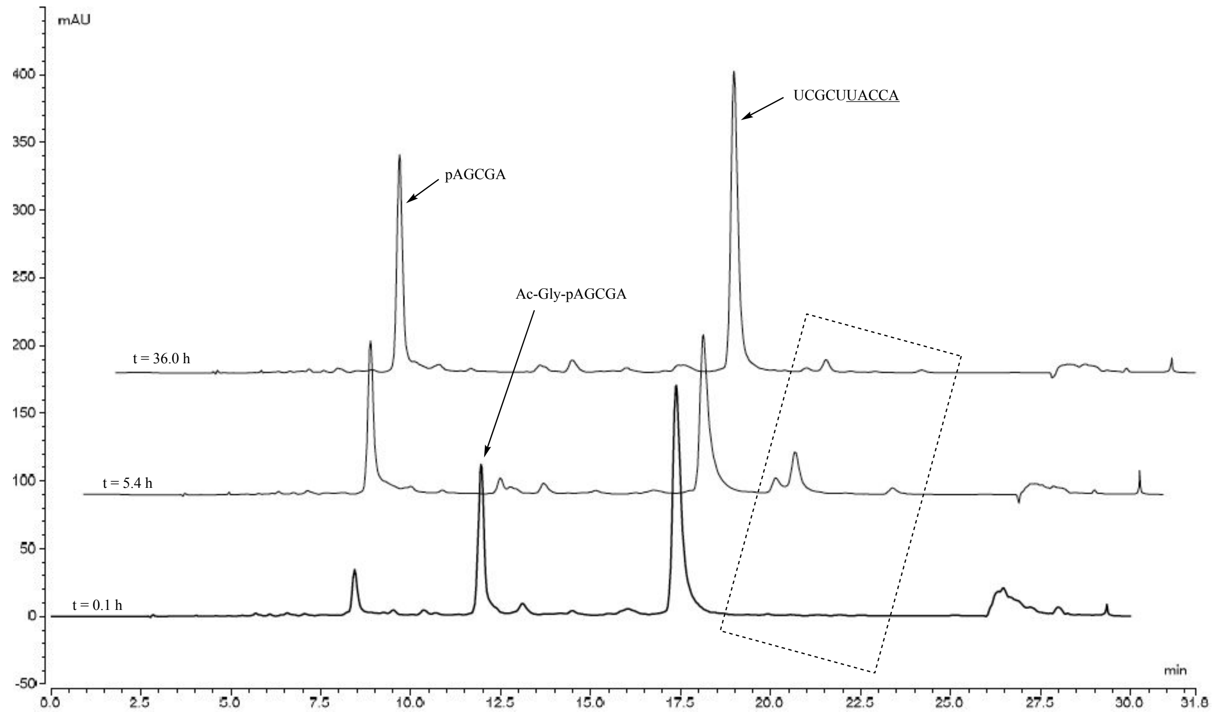

Fig. S29. Ac-Gly transfer in a tRNA acceptor arm mimic. Loop duplex sequence:

`5′UCGCUUACCA`

`3′AGCGAp-Gly-Ac`

Transfer was monitored using HPLC with 260 nm UV detection. The solution was incubated at 20°C and aliquots of 8 μL were injected into an HPLC at different time points. Peaks for the donor, the donor mixed anhydride and acceptor strands are indicated. The peak presumed to be due to the diol ester transfer product is highlighted by the dashed box. Conditions: both oligos (100 μM), NaCl (1000 mM), HEPES (100 mM, pH 7.5).

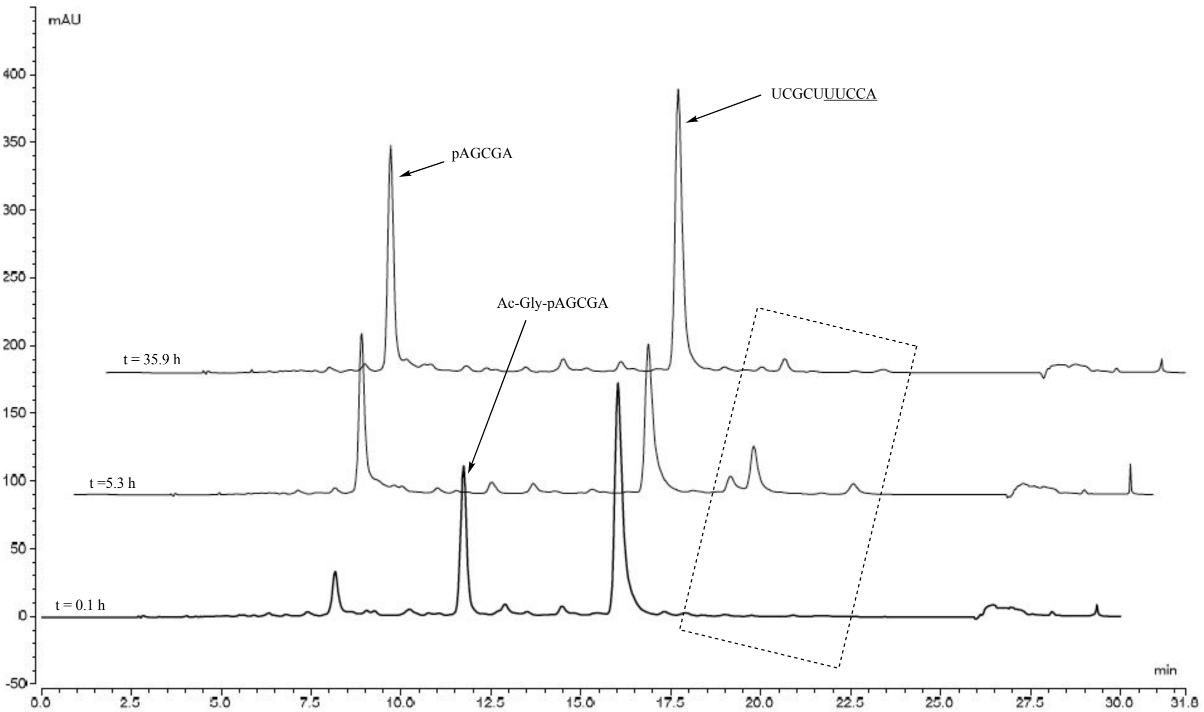

Fig. S30. Ac-Gly transfer in a tRNA acceptor arm mimic. Loop duplex sequence:

5′UCGCUUUCCA

3′AGCGAp-Gly-Ac

Transfer was monitored using HPLC with 260 nm UV detection. The solution was incubated at 20°C and aliquots of 8 μL were injected into an HPLC at different time points. Peaks for the donor, the donor mixed anhydride and acceptor strands are indicated. Peaks presumed to be due to the diol ester transfer product are highlighted by the dashed box. Conditions: both oligos (100 μM), NaCl (1000 mM), HEPES (100 mM, pH 7.5).

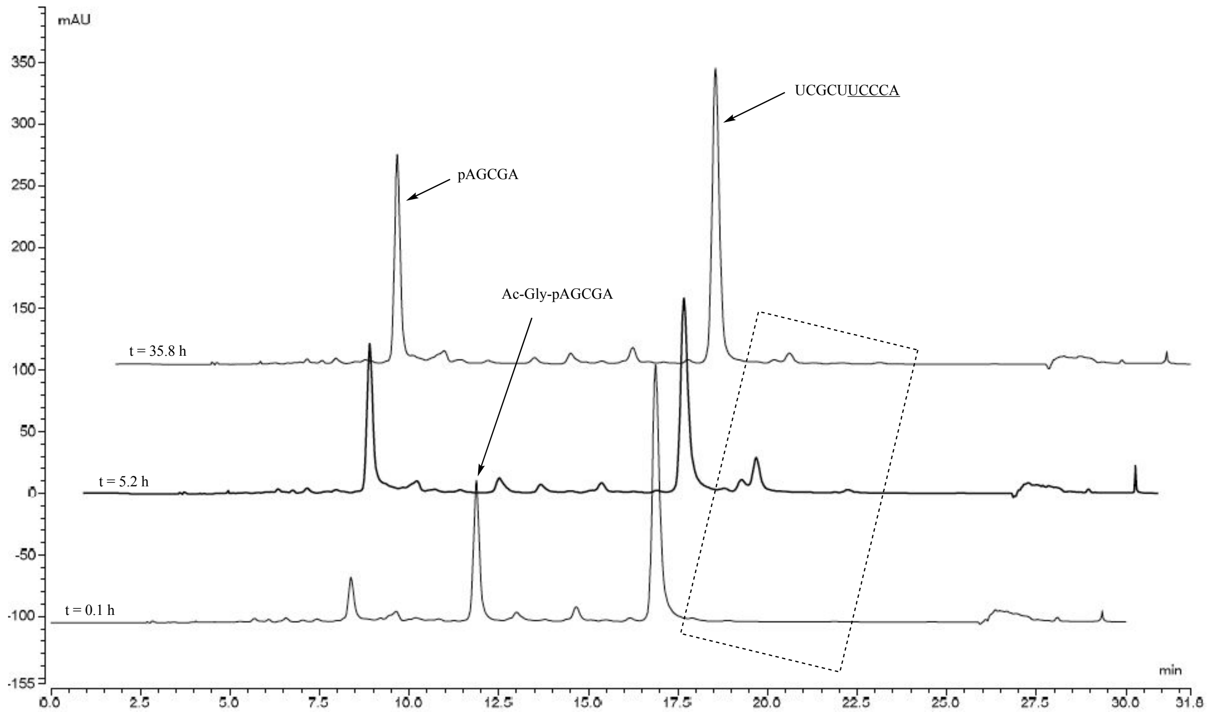

Fig. S31. Ac-Gly transfer in a tRNA acceptor arm mimic. Loop duplex sequence:

5′UCGCU<u>UCCCA</u>

3′AGCGAp-Gly-Ac

Transfer was monitored using HPLC with 260 nm UV detection. The solution was incubated at 20°C and aliquots of 8 µL were injected into an HPLC at different time points. Peaks for the donor, the donor mixed anhydride and acceptor strands are indicated. Peaks presumed to be due to the diol ester transfer product are highlighted by the dashed box. Conditions: both oligos (100 µM), NaCl (1000 mM), HEPES (100 mM, pH 7.5).

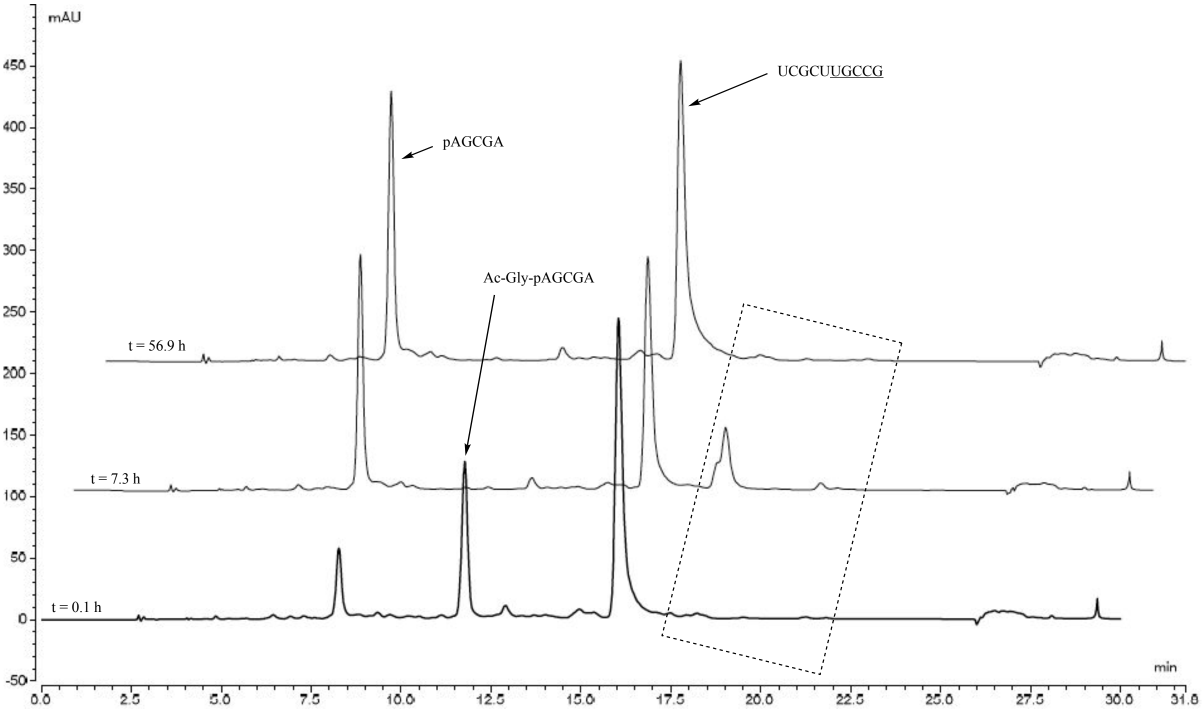

Fig. S32. Ac-Gly transfer in a tRNA acceptor arm mimic. Loop duplex sequence:

5′UCGCU<u>UGCCG</u>

3′AGCGAp-Gly-Ac

Transfer was monitored using HPLC with 260 nm UV detection. The solution was incubated at 20°C and aliquots of 8 μL were injected into an HPLC at different time points. Peaks for the donor, the donor mixed anhydride and acceptor strands are indicated. Peaks presumed to be due to the diol ester transfer product are highlighted by the dashed box. Conditions: both oligos (100 μM), NaCl (1000 mM), HEPES (100 mM, pH 7.5).

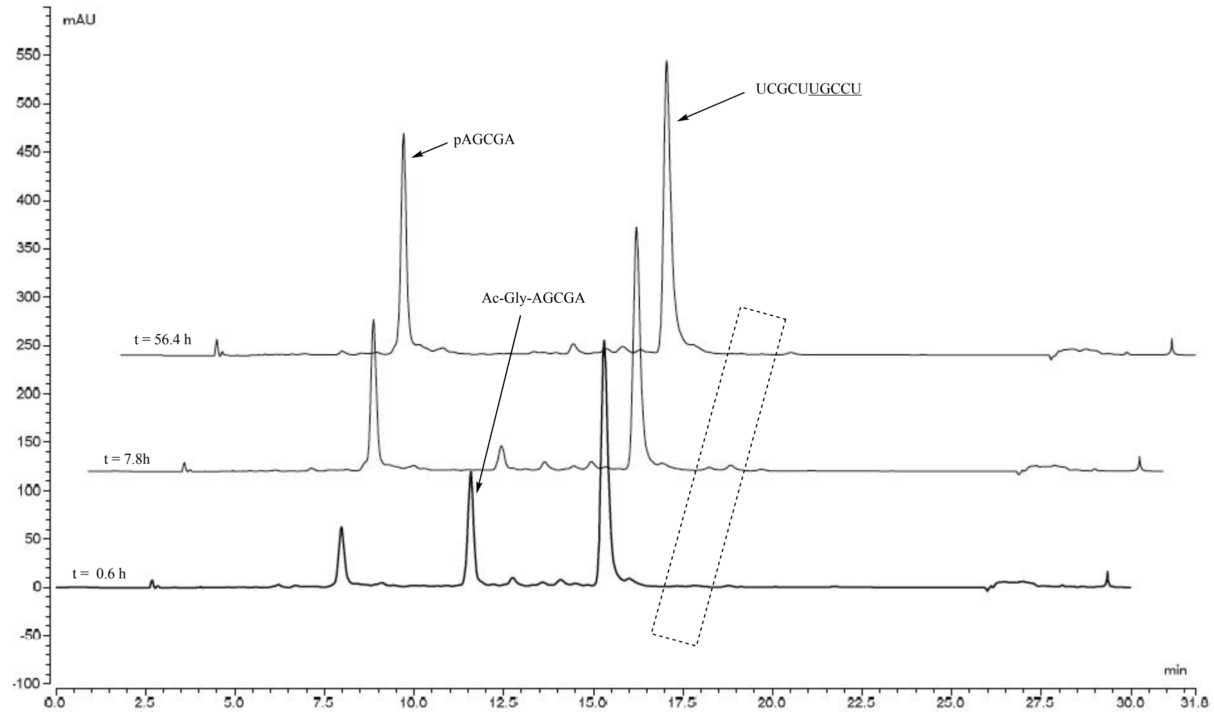

Fig. S33. Ac-Gly transfer in a tRNA acceptor arm mimic. Loop duplex sequence:

5′UCGCUUGCCU

3′AGCGAp-Gly-Ac

Transfer was monitored using HPLC with 260 nm UV detection. The solution was incubated at 20°C and aliquots of 8 μL were injected into an HPLC at different time points. Peaks for the donor, the donor mixed anhydride and acceptor strands are indicated. Peak presumed to be due to the diol ester transfer product are highlighted by the dashed box. Conditions: both oligos (100 μM), NaCl (1000 mM), HEPES (100 mM, pH 7.5).

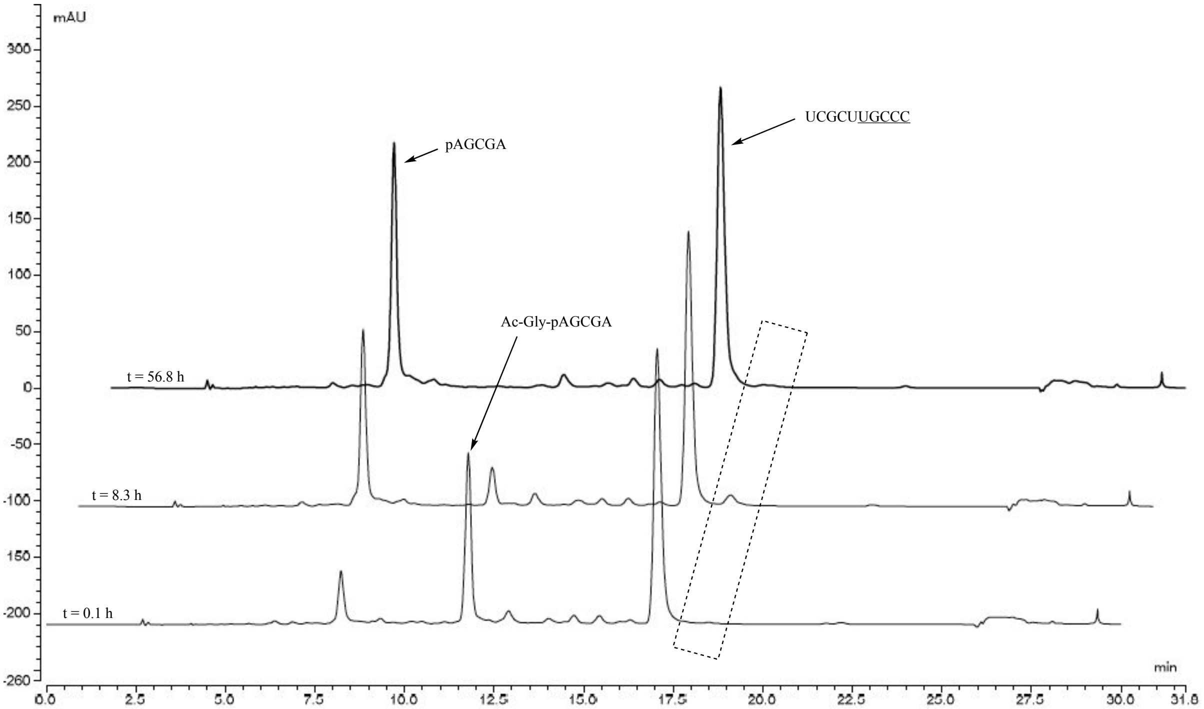

Fig. S34. Ac-Gly transfer in a tRNA acceptor arm mimic. Loop duplex sequence:

`5’UCGCUUGCCC`

`3’AGCGAp-Gly-Ac`

Transfer was monitored using HPLC with 260 nm UV detection. The solution was incubated at 20°C and aliquots of 8 μL were injected into an HPLC at different time points. Peaks for the donor, the donor mixed anhydride and acceptor strands are indicated. The peak presumed to be due to the diol ester transfer product is highlighted by the dashed box. Conditions: both oligos (100 μM), NaCl (1000 mM), HEPES (100 mM, pH 7.5).

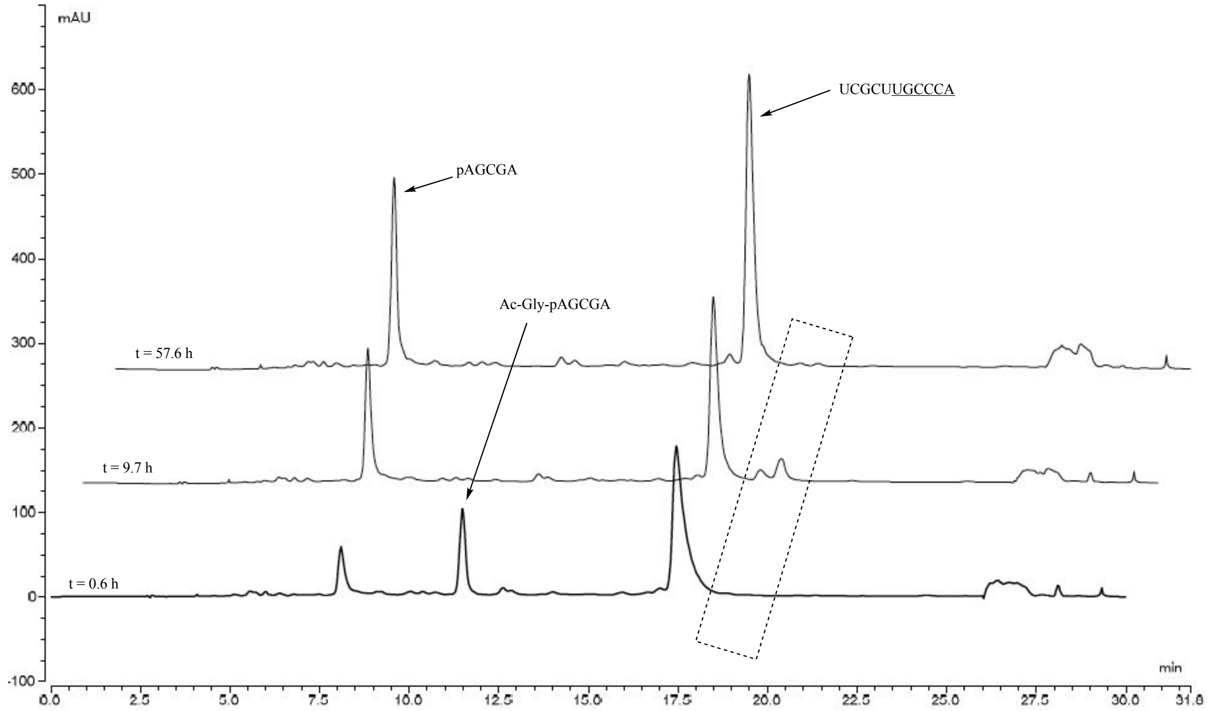

Fig. S35. Ac-Gly transfer in a tRNA acceptor arm mimic. Loop duplex sequence:

5′UCGCU<u>UGCCCA</u>

3′AGCGAp-Gly-Ac

Transfer was monitored using HPLC with 260 nm UV detection. The solution was incubated at 20°C and aliquots of 8 μL were injected into an HPLC at different time points. Peaks for the donor, the donor mixed anhydride and acceptor strands are indicated. Peaks presumed to be due to the diol ester transfer product are highlighted by the dashed box. Conditions: both oligos (100 μM), NaCl (1000 mM), HEPES (100 mM, pH 7.5).

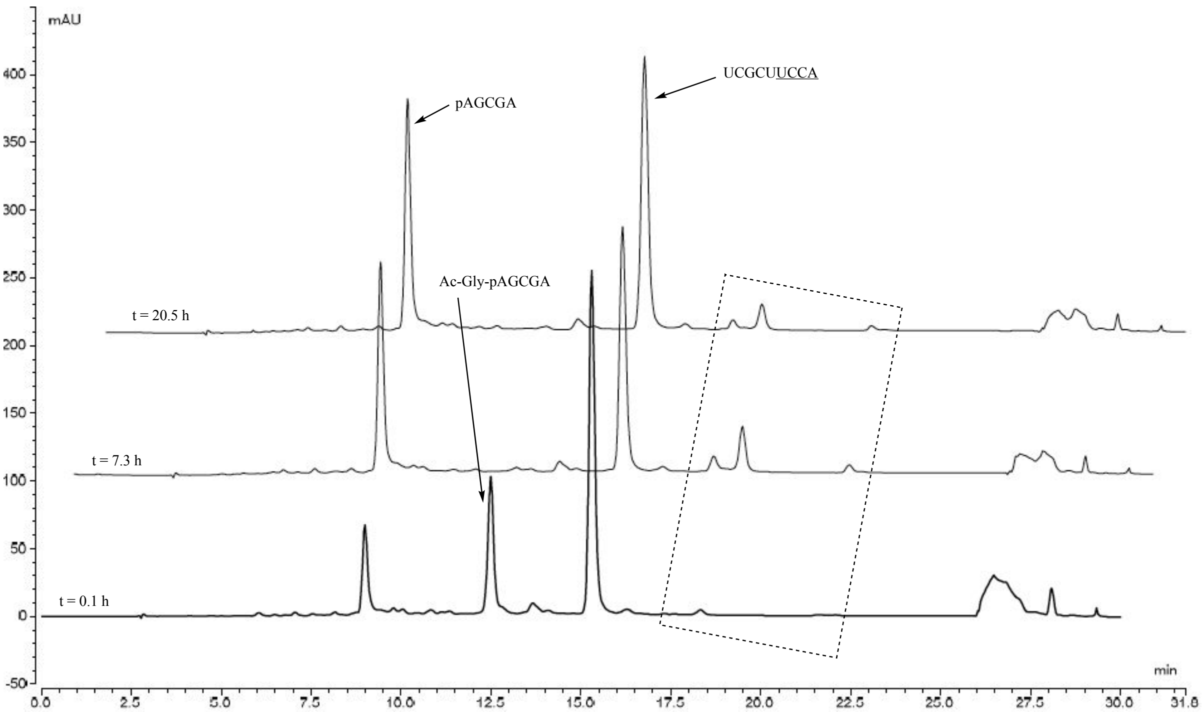

Fig. S36. Ac-Gly transfer in a tRNA acceptor arm mimic. Loop duplex sequence:

```
5’UCGCUUCCA
3’AGCGAp-Gly-Ac
```

Transfer was monitored using HPLC with 260 nm UV detection. The solution was incubated at 20°C and aliquots of 8 μL were injected into an HPLC at different time points. Peaks for the donor, the donor mixed anhydride and acceptor strands are indicated. Peaks presumed to be due to the diol ester transfer product are highlighted by the dashed box. Conditions: both oligos (100 μM), NaCl (1000 mM), HEPES (100 mM, pH 7.5).

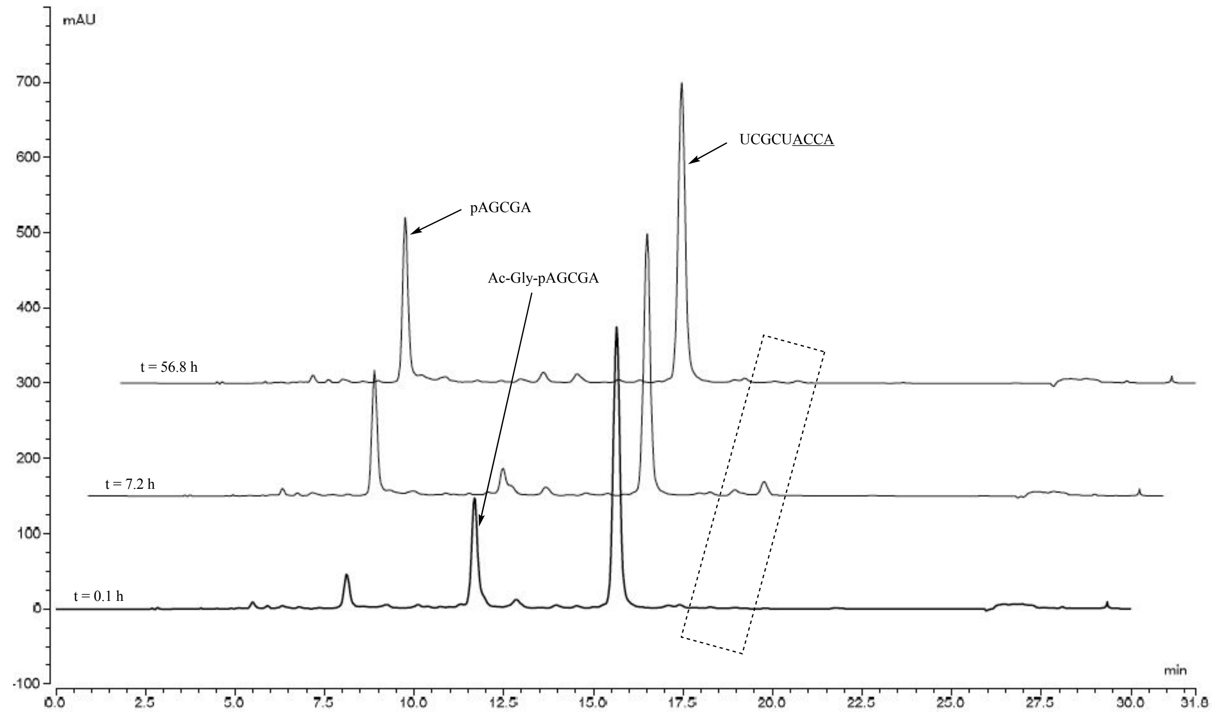

Fig. S37. Ac-Gly transfer in a tRNA acceptor arm mimic. Loop duplex sequence:

5′UCGCU<u>ACCA</u>

3′AGCGAp-Gly-Ac

Transfer was monitored using HPLC with 260 nm UV detection. The solution was incubated at 20°C and aliquots of 8 μL were injected into an HPLC at different time points. Peaks for the donor, the donor mixed anhydride and acceptor strands are indicated. Peaks presumed to be due to the diol ester transfer product are highlighted by the dashed box. Conditions: both oligos (100 μM), NaCl (1000 mM), HEPES (100 mM, pH 7.5).

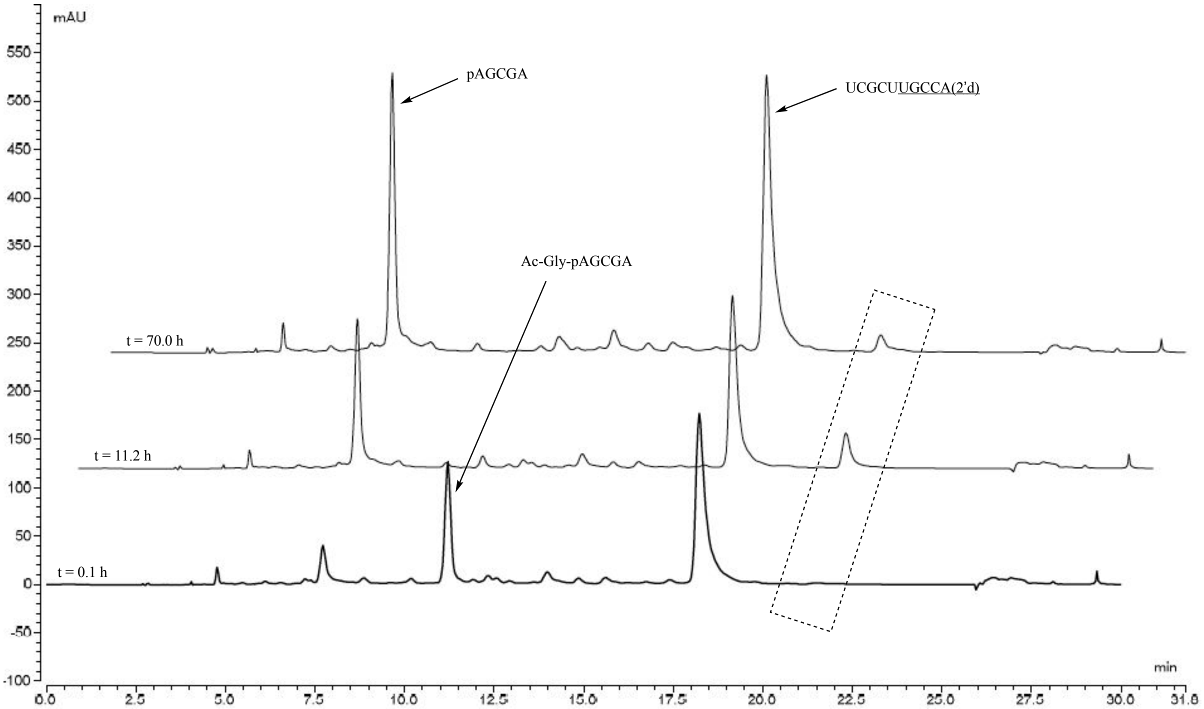

Fig. S38. Ac-Gly transfer in a tRNA acceptor arm mimic. Loop duplex sequence:

5′UCGCUUGCCA(2′d)

3′AGCGAp-Gly-Ac

Transfer was monitored using HPLC with 260 nm UV detection. The solution was incubated at 20°C and aliquots of 8 μL were injected into an HPLC at different time points. Peaks for the donor, the donor mixed anhydride and acceptor strands are indicated. The peak presumed to be due to the diol ester transfer product is highlighted by the dashed box. Conditions: both oligos (100 μM), NaCl (1000 mM), HEPES (100 mM, pH 7.5).

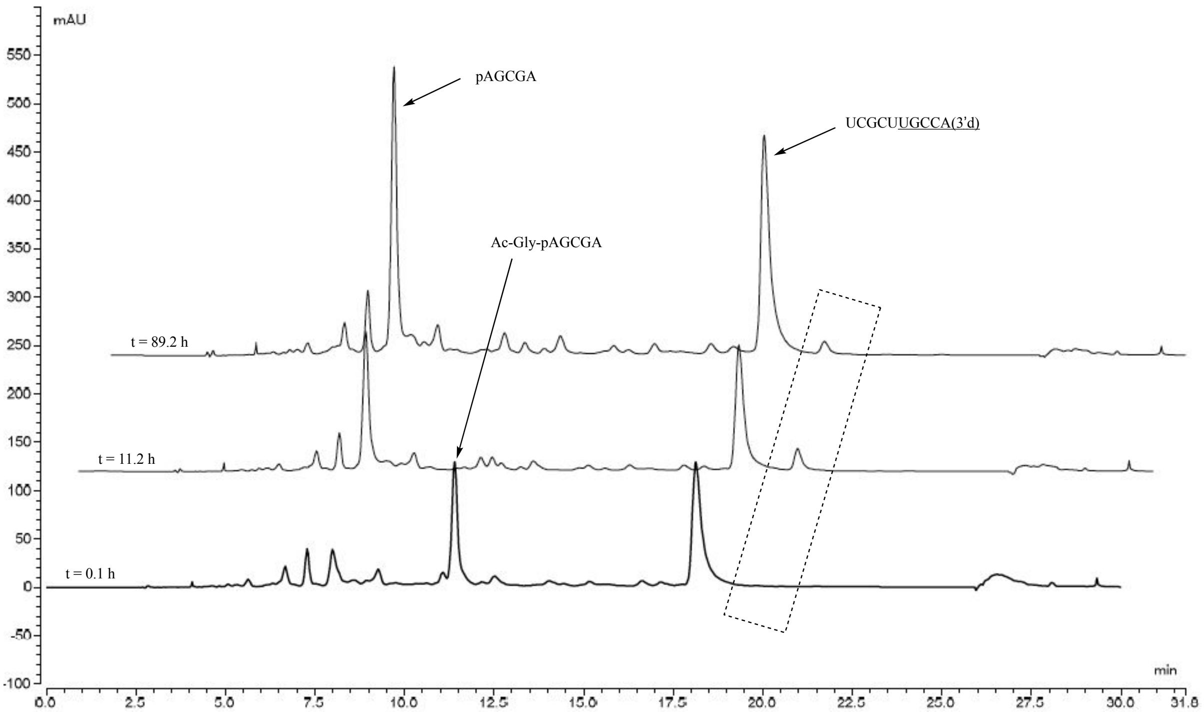

Fig. S39. Ac-Gly transfer in a tRNA acceptor arm mimic. Loop duplex sequence:

`5’UCGCUUGCCA(3’d)`

`3’AGCGAp-Gly-Ac`

Transfer was monitored using HPLC with 260 nm UV detection. The solution was incubated at 20°C and aliquots of 8 µL were injected into an HPLC at different time points. Peaks for the donor, the donor mixed anhydride and acceptor strands are indicated. The peak presumed to be due to the diol ester transfer product is highlighted by the dashed box. Conditions: both oligos (100 µM), NaCl (1000 mM), HEPES (100 mM, pH 7.5).

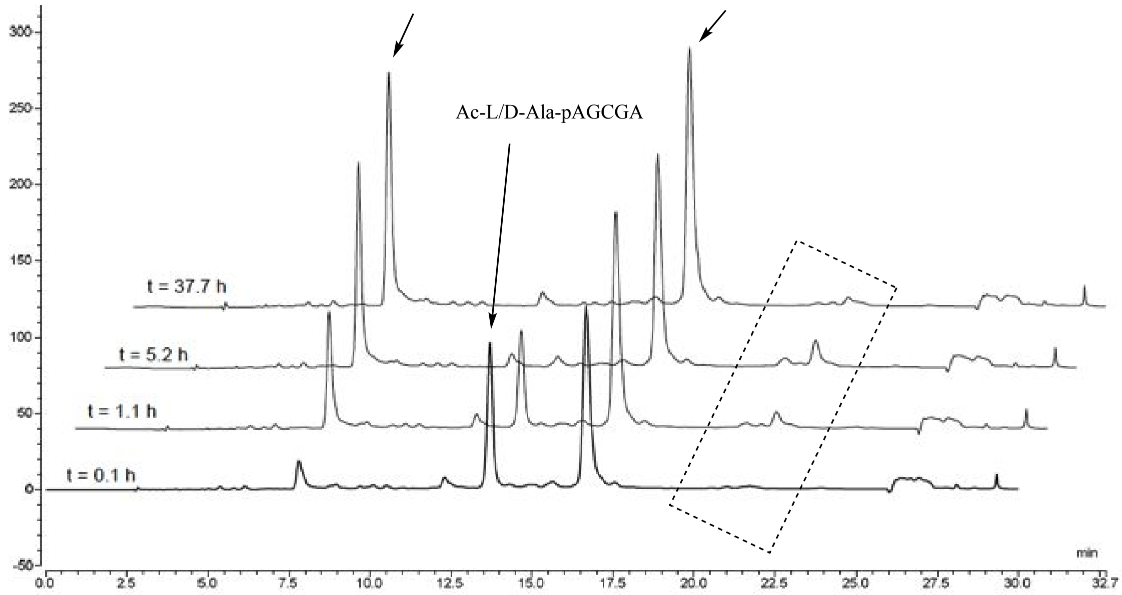

Fig. S40. Ac-L/D-Ala transfer in a tRNA acceptor arm. Loop duplex sequence:

5′UCGCUUGCCA

3′AGCGAp-L/D-Ala-Ac

Transfer was monitored using HPLC with 260 nm UV detection. The solution was incubated at 20 °C and aliquots of 8 μL were injected into an HPLC at different time points. Peaks for the donor, the donor mixed anhydride and acceptor strands are indicated. Peaks presumed to be due to the diol ester transfer products are highlighted by the dashed box. Conditions: both oligos (100 μM), NaCl (1000 mM), HEPES (100 mM, pH 7.5).

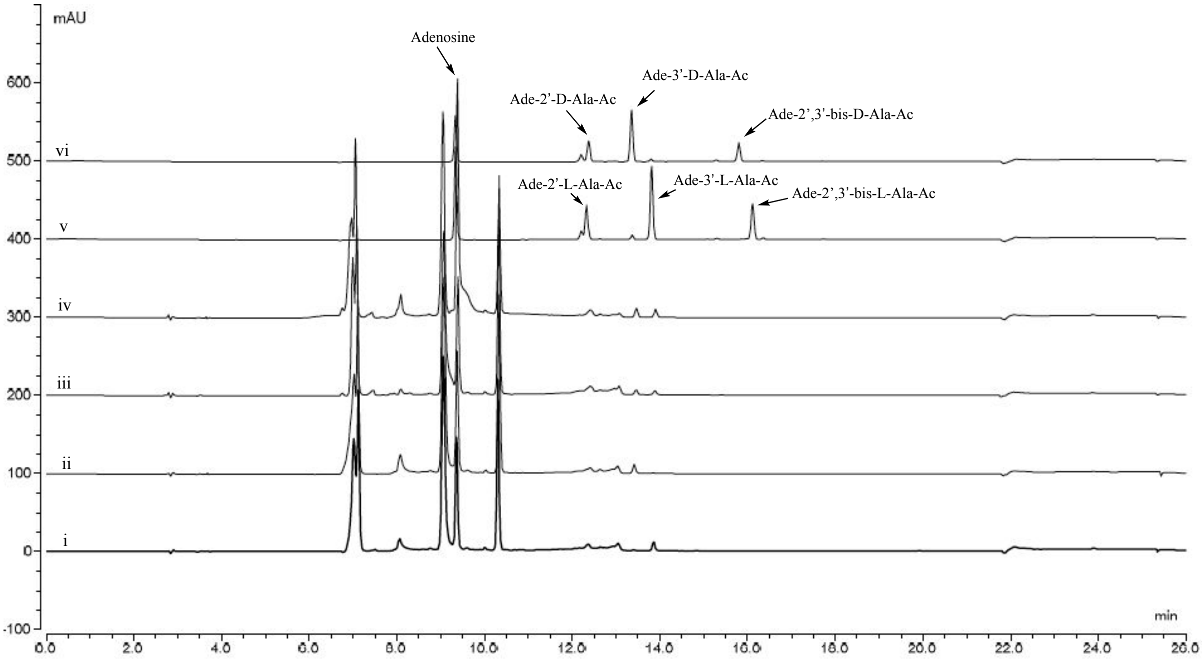

Fig. S41. Enzyme digestion experiment to confirm the acylation position and the stereoselectivity of *N*-Ac-Ala transfer. i) A reaction mixture (100 µL) comprising Ac-L-Ala-pAGCGA (100 µM), UGCGUUGCCA (100 µM), NaCl (1 M), HEPES (100 mM) at pH 7.5 was incubated for 2 hours at 20°C. Then aliquots of 9 µL were added to 1 µL of quenching buffer after 6 hours. 0.2 µL of RNase A (10 mg/mL) was added and incubated at 20°C for 30 min. 10 µL of methanol was added, and the resulting mixture was centrifugated. The supernatant was analyzed by HPLC with 260 nm UV detection and compared with chromoatograms of synthetic standards of Ade-2'/3'-L-Ala-Ac and Ade Ade-2'/3'-D-Ala-Ac, respectively. ii) Reaction as described in i), but using Ac-D-Ala-pAGCGA instead of Ac-L-Ala-pAGCGA. iii) Reaction as described in i), but using Ac-L-Ala-pAGCGA and Ac-D-pAGCGA in 1 : 1 ratio instead of Ac-L-Ala-pAGCGA. iv) Reaction as described in iii), but also including UGCCA (100 uM), which leads to a nicked duplex transfer. v) Synthetic standard of Ade-2'/3'-L-Ala-Ac, including Ade-2',3'-bis-L-Ala-Ac and adenosine. vi) Synthetic standard of Ade-2'/3'-D-Ala-Ac, including Ade-2',3'-bis-D-Ala-Ac and adenosine.

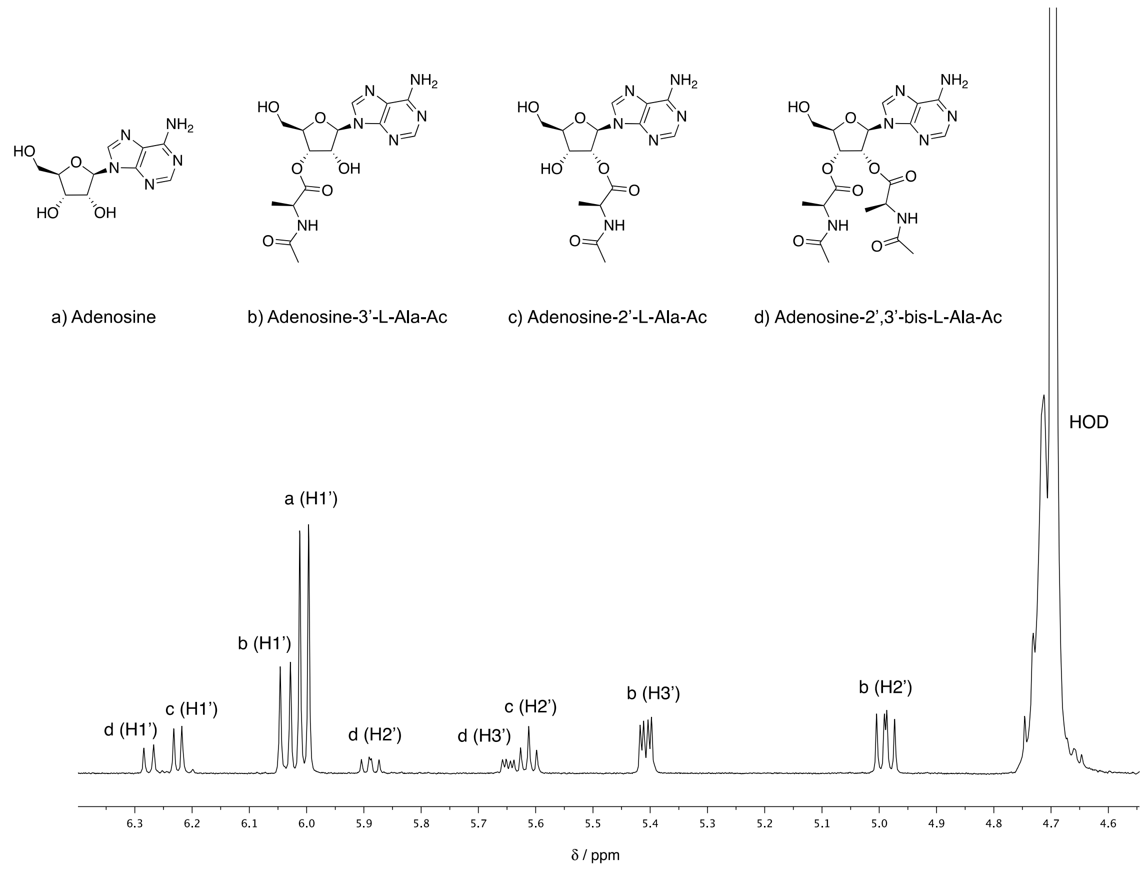

Fig. S42. ^{1}H-NMR spectrum of synthetic standard of *N*-acetyl-L-alanyl diol ester of adenosine (adenosine-3'-L-Ala-Ac and adenosine-2'-L-Ala-Ac), including adenosine and adenosine-2',3'-bis-L-Ala-Ac.

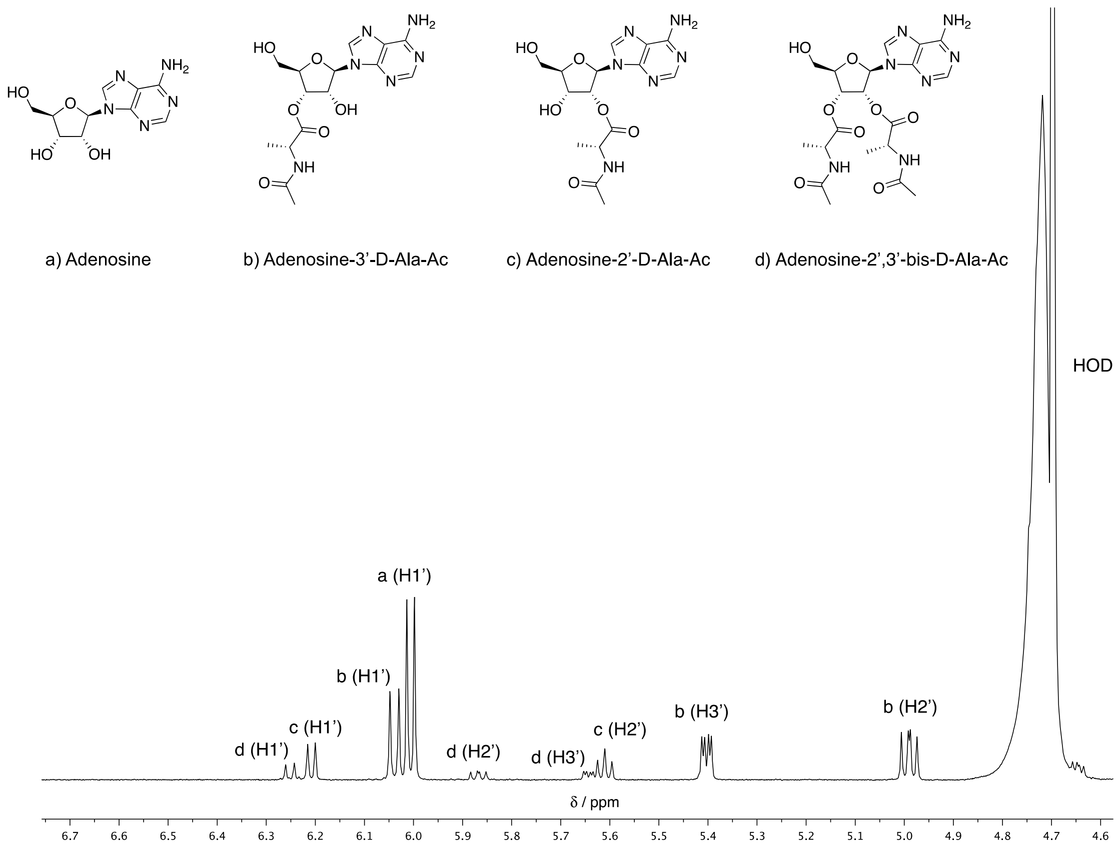

Fig. S43. ^{1}H-NMR spectrum of synthetic standard of *N*-acetyl-D-alanyl diol ester of adenosine (adenosine-3'-D-Ala-Ac and adenosine-2'-D-Ala-Ac), including adenosine and adenosine-2',3'-bis-D-Ala-Ac.

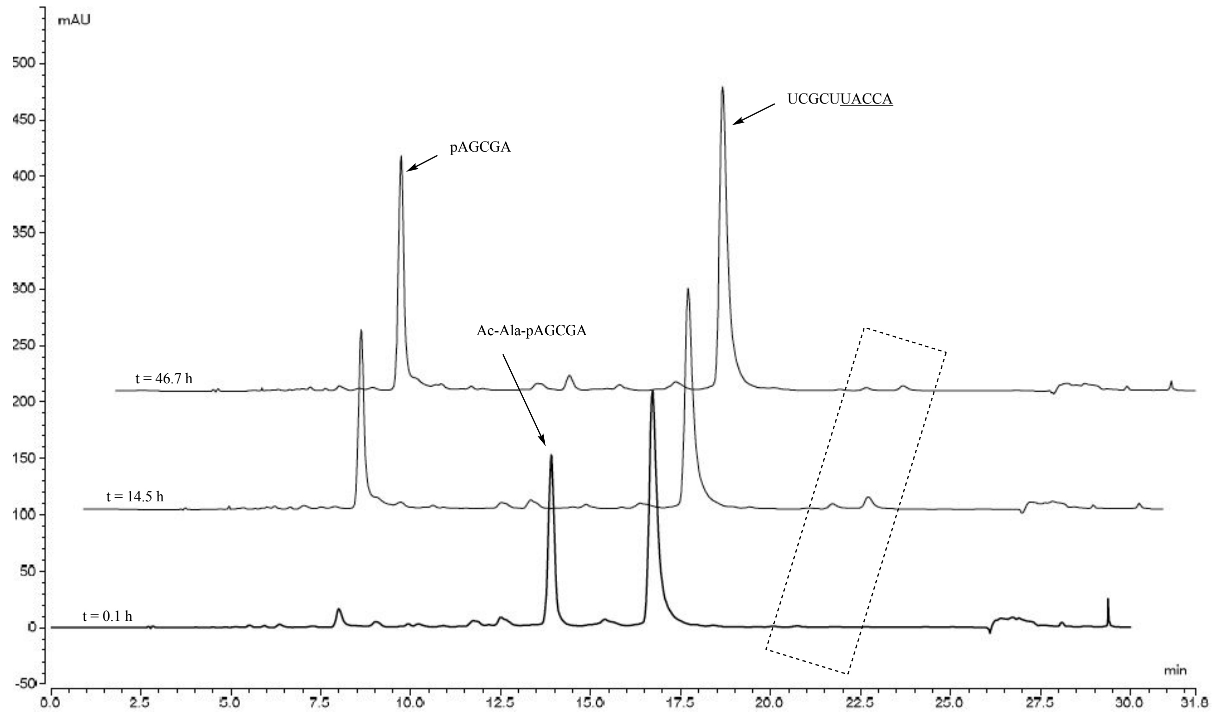

Fig. S44. Ac-L/D-Ala transfer in a tRNA acceptor arm mimic. Loop duplex sequence:

5′UCGCUUACCA

3′AGCGAp-L/D-Ala-Ac

Transfer was monitored using HPLC with 260 nm UV detection. The solution was incubated at 20°C and aliquots of 8 μL were injected into an HPLC at different time points. Peaks for the donor, the donor mixed anhydride and acceptor strands are indicated. Peaks presumed to be due to the diol ester transfer product are highlighted by the dashed box. Conditions: both oligos (100 μM), NaCl (1000 mM), HEPES (100 mM, pH 7.5).

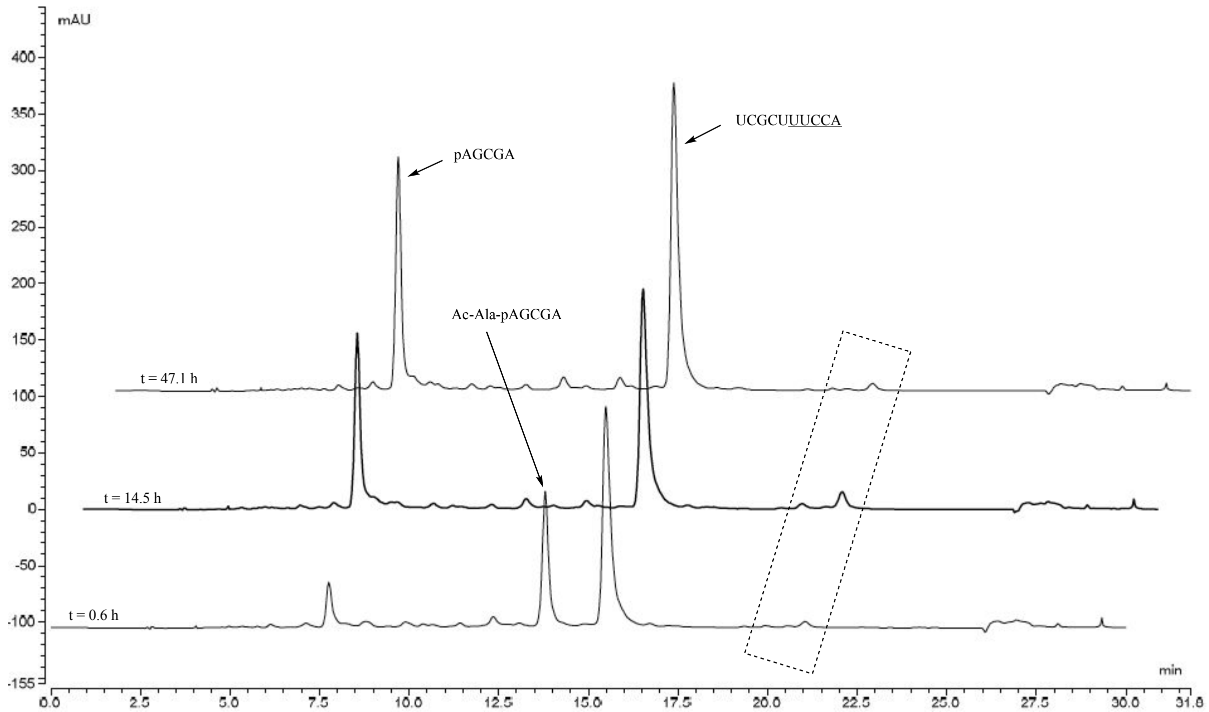

Fig. S45. Ac-L/D-Ala transfer in a tRNA acceptor arm mimic. Loop duplex sequence:

`5′UCGCUUUCCA`

`3′AGCGAp-L/D-Ala-Ac`

Transfer was monitored using HPLC with 260 nm UV detection. The solution was incubated at 20°C and aliquots of 8 µL were injected into an HPLC at different time points. Peaks for the donor, the donor mixed anhydride and acceptor strands are indicated. Peaks presumed to be due to the diol ester transfer product are highlighted by the dashed box. Conditions: both oligos (100 µM), NaCl (1000 mM), HEPES (100 mM, pH 7.5).

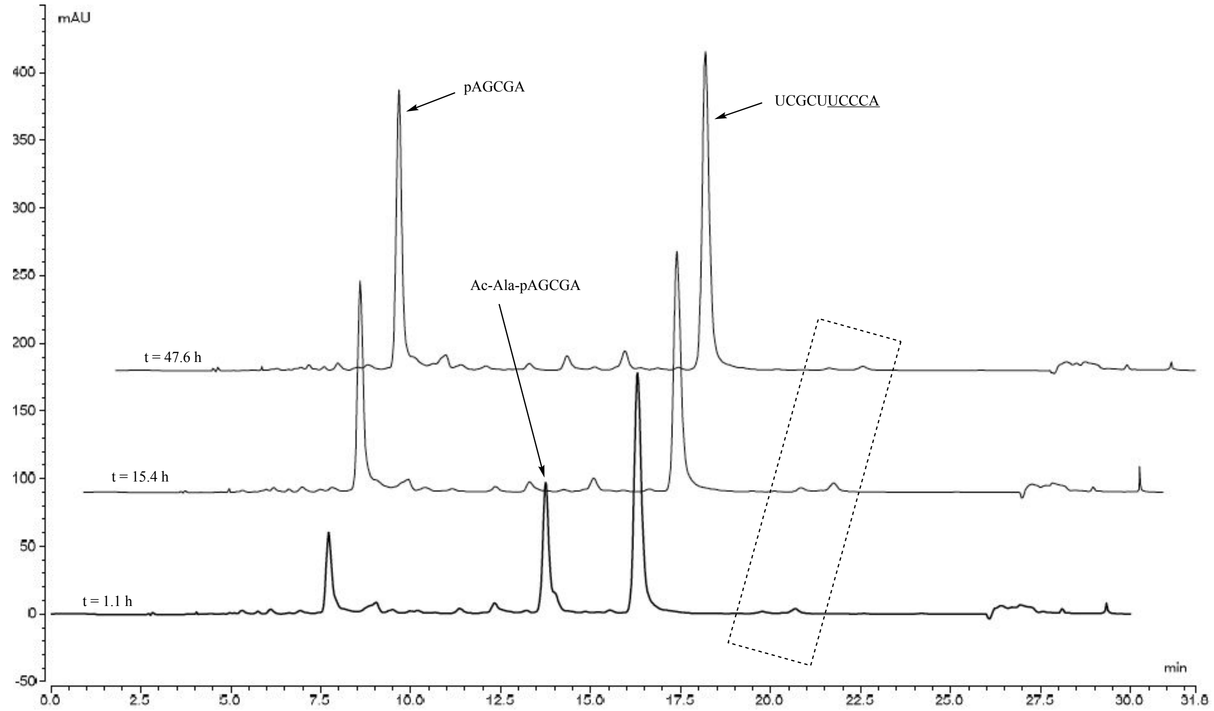

Fig. S46. Ac-L/D-Ala transfer in a tRNA acceptor arm mimic. Loop duplex sequence:

5′UCGCU<u>UCCCA</u>

3′AGCGAp-L/D-Ala-Ac

Transfer was monitored using HPLC with 260 nm UV detection. The solution was incubated at 20°C and aliquots of 8 µL were injected into an HPLC at different time points. Peaks for the donor, the donor mixed anhydride and acceptor strands are indicated. Peaks presumed to be due to the diol ester transfer product are highlighted by the dashed box. Conditions: both oligos (100 µM), NaCl (1000 mM), HEPES (100 mM, pH 7.5).

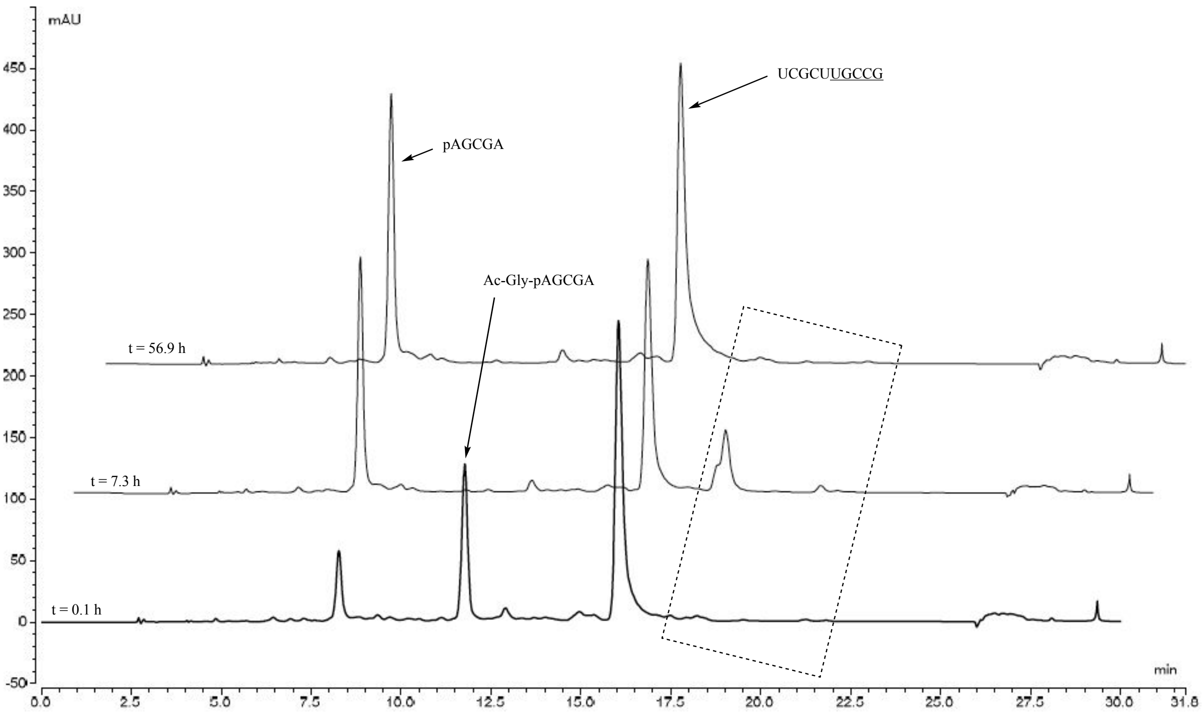

Fig. S47. Ac-L/D-Ala transfer in a tRNA acceptor arm mimic. Loop duplex sequence:

5′UCGCUUGCCG

3′AGCGAp-L/D-Ala-Ac

Transfer was monitored using HPLC with 260 nm UV detection. The solution was incubated at 20°C and aliquots of 8 μL were injected into an HPLC at different time points. Peaks for the donor, the donor mixed anhydride and acceptor strands are indicated. Peaks presumed to be due to the diol ester transfer product are highlighted by the dashed box. Conditions: both oligos (100 μM), NaCl (1000 mM), HEPES (100 mM, pH 7.5).

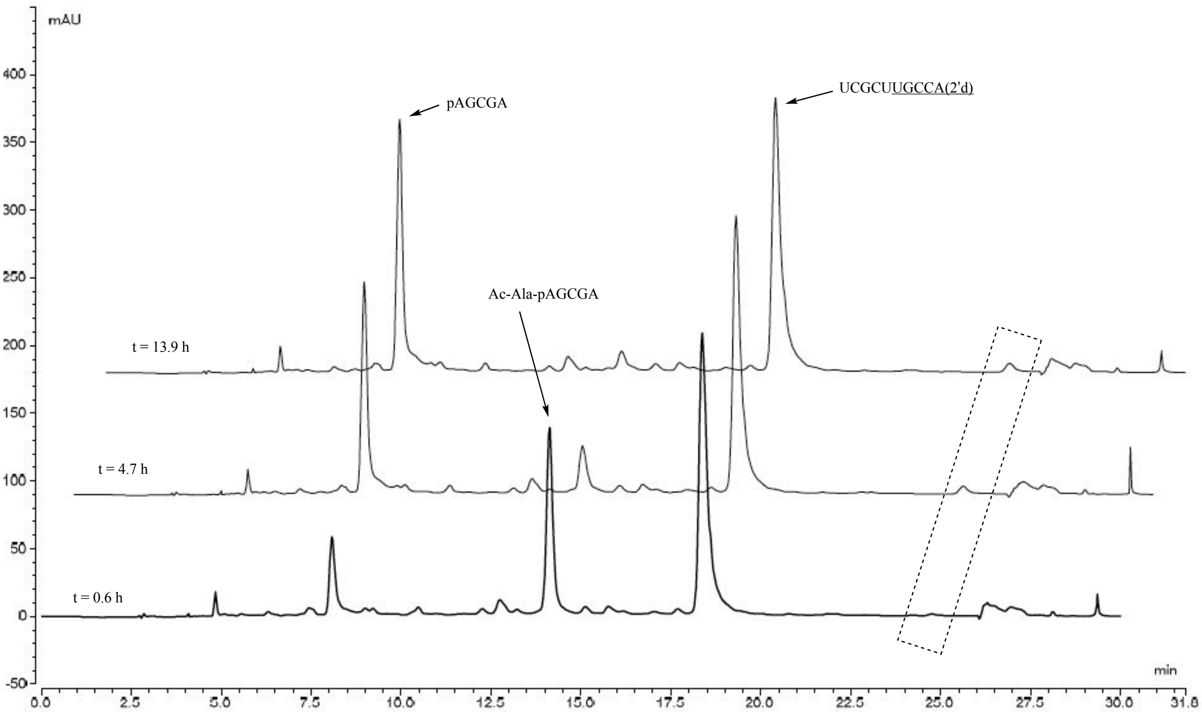

Fig. S48. Ac-L/D-Ala transfer in a tRNA acceptor arm mimic. Loop duplex sequence:

`5'UCGCUUGCCA(2'd)`

`3'AGCGAp-L/D-Ala-Ac`

Transfer was monitored using HPLC with 260 nm UV detection. The solution was incubated at 20°C and aliquots of 8 μL were injected into an HPLC at different time points. Peaks for the donor, the donor mixed anhydride and acceptor strands are indicated. Peaks presumed to be due to the diol ester transfer product are highlighted by the dashed box. Conditions: both oligos (100 μM), NaCl (1000 mM), HEPES (100 mM, pH 7.5).

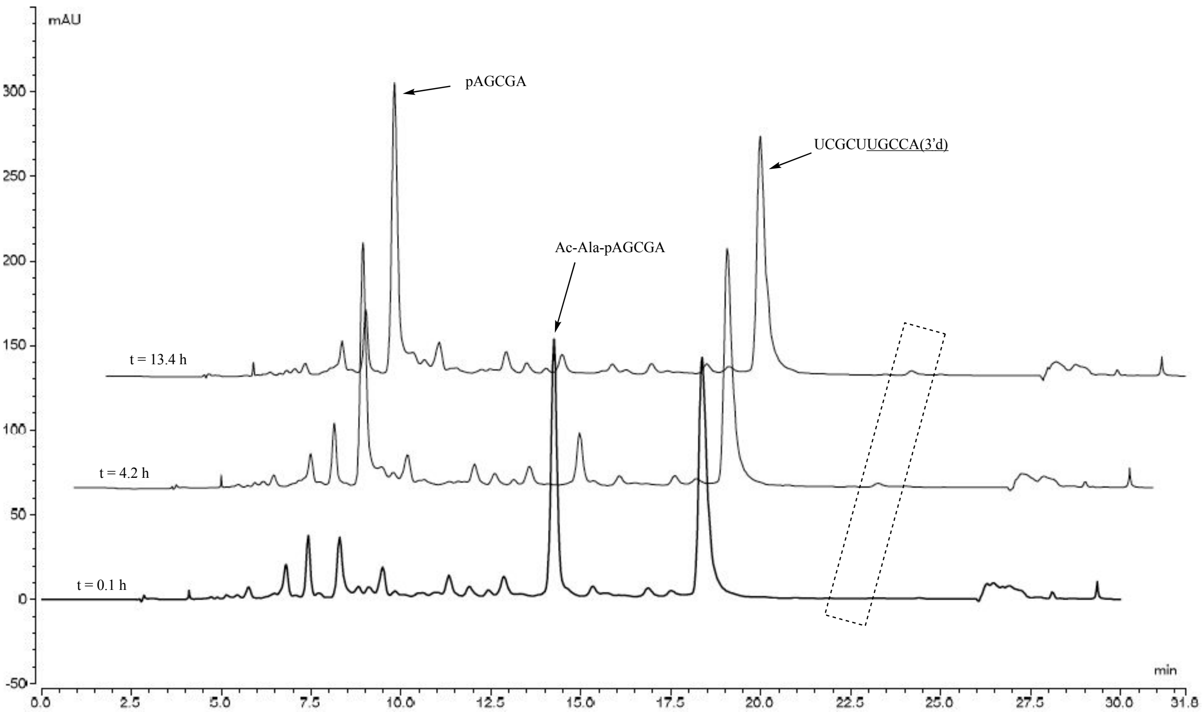

Fig. S49. Ac-L/D-Ala transfer in a tRNA acceptor arm mimic. Loop duplex sequence:

5′UCGCUUGCCA(3′d)

3′AGCGAp-L/D-Ala-Ac

Transfer was monitored using HPLC with 260 nm UV detection. The solution was incubated at 20°C and aliquots of 8 μL were injected into an HPLC at different time points. Peaks for the donor, the donor mixed anhydride and acceptor strands are indicated. The peak presumed to be due to the diol ester transfer product is highlighted by the dashed box. Conditions: both oligos (100 μM), NaCl (1000 mM), HEPES (100 mM, pH 7.5).

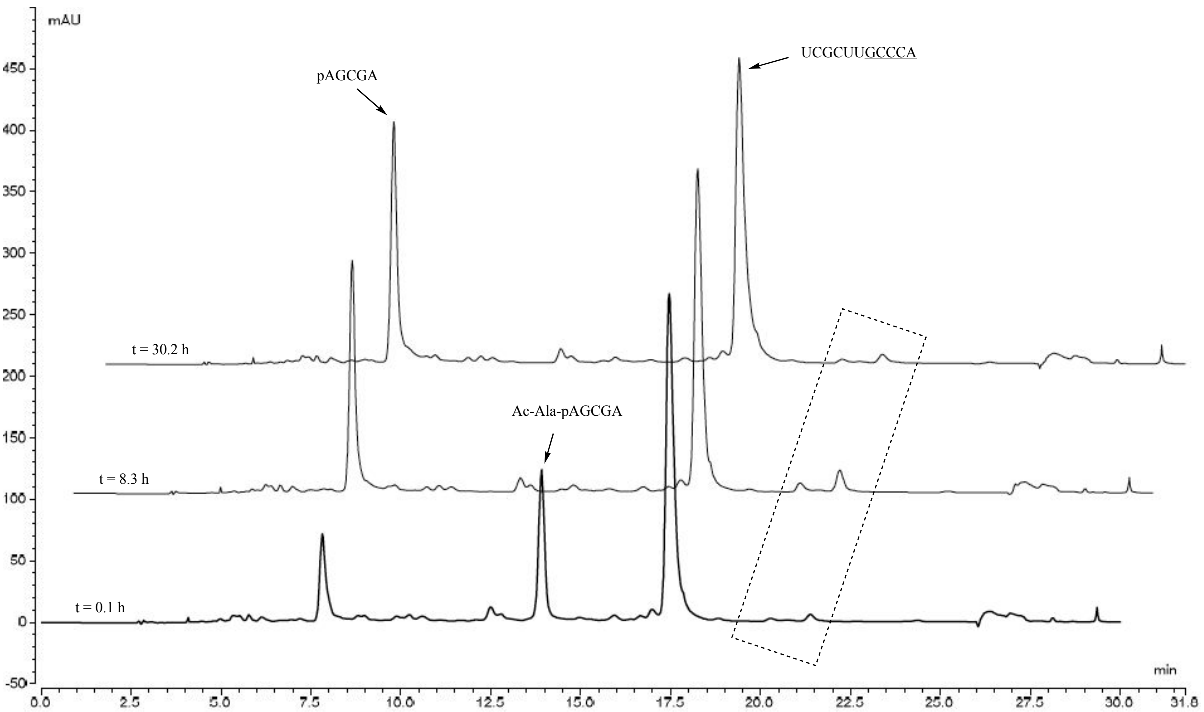

Fig. S50. Ac-L/D-Ala transfer in a tRNA acceptor arm mimic. Loop duplex sequence:

5′UCGCUUGCCCA

3′AGCGAp-L/D-Ala-Ac

Transfer was monitored using HPLC with 260 nm UV detection. The solution was incubated at 20°C and aliquots of 8 μL were injected into an HPLC at different time points. Peaks for the donor, the donor mixed anhydride and acceptor strands are indicated. Peaks presumed to be due to the diol ester transfer product are highlighted by the dashed box. Conditions: both oligos (100 μM), NaCl (1000 mM), HEPES (100 mM, pH 7.5).

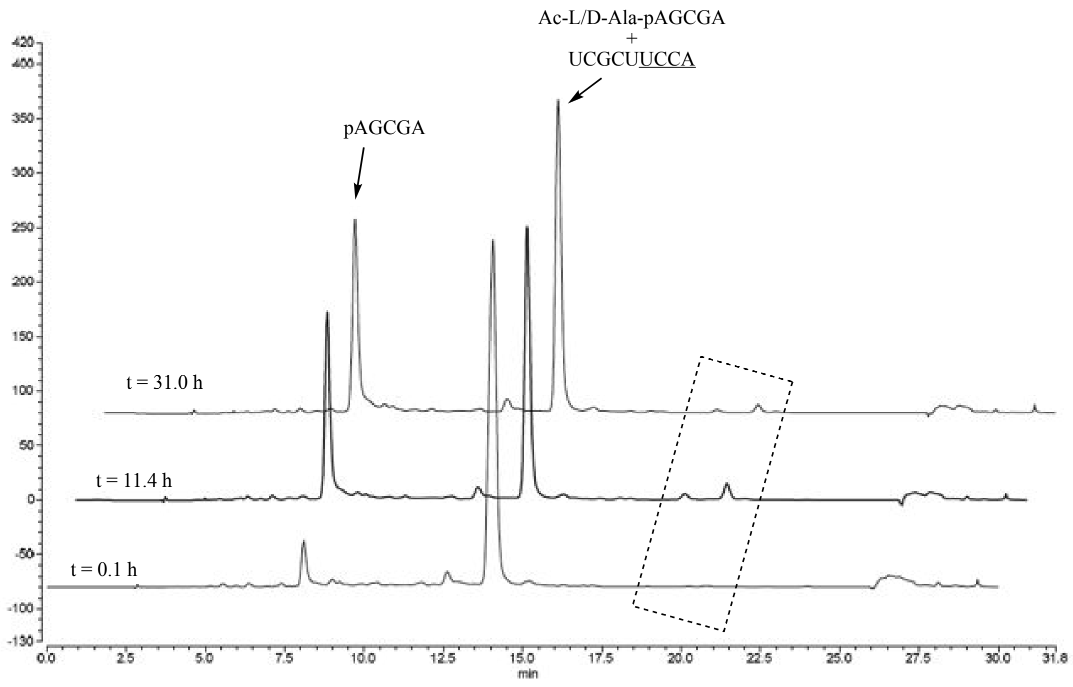

Fig. S51. Ac-L/D-Ala transfer in a tRNA acceptor arm mimic. Loop duplex sequence:

5′UCGCUUCCA

3′AGCGAp-L/D-Ala-Ac

Transfer was monitored using HPLC with 260 nm UV detection. The solution was incubated at 20°C and aliquots of 8 μL were injected into an HPLC at different time points. Peaks for the donor, the donor mixed anhydride and acceptor strands are indicated. Peaks presumed to be due to the diol ester transfer product are highlighted by the dashed box. Conditions: both oligos (100 μM), NaCl (1000 mM), HEPES (100 mM, pH 7.5).

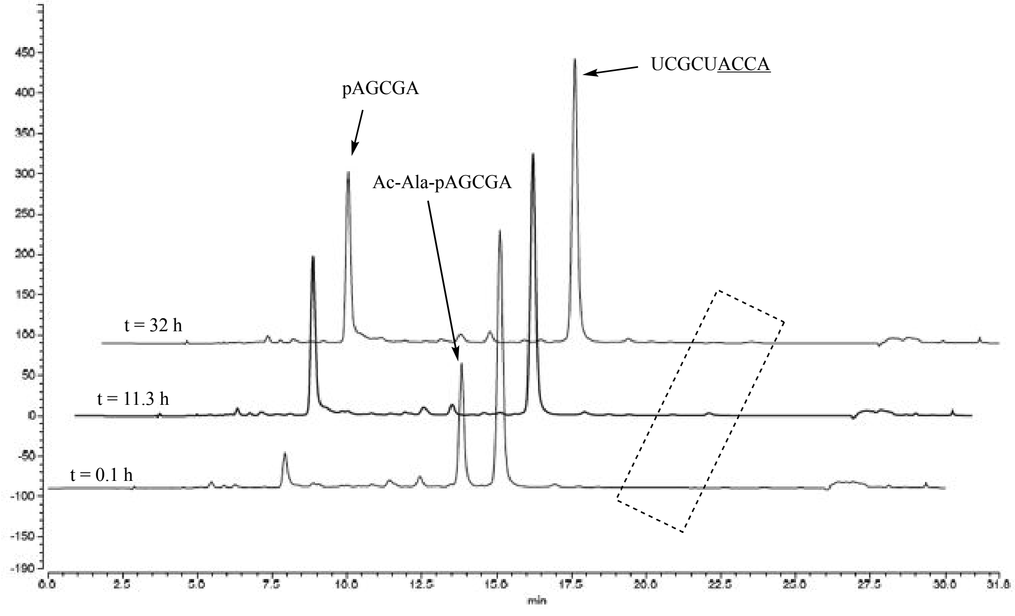

Fig. S52. Ac-L/D-Ala transfer in a tRNA acceptor arm mimic. Loop duplex sequence:

5′UCGCUACCA

3′AGCGAp-L/D-Ala-Ac

Transfer was monitored using HPLC with 260 nm UV detection. The solution was incubated at 20°C and aliquots of 8 μL were injected into an HPLC at different time points. Peaks for the donor, the donor mixed anhydride and acceptor strands are indicated. Peaks presumed to be due to the diol ester transfer product are highlighted by the dashed box. Conditions: both oligos (100 μM), NaCl (1000 mM), HEPES (100 mM, pH 7.5).

Table S1. Screening of conditions for L-Ala transfer. Oligo and buffer concentrations: 100 µM of (pAGCGA + L-Ala-pAGCGA), 100 µM of UCGCUUGCCA, 50 mM HEPES in a solution with different salts, pH and temperature.

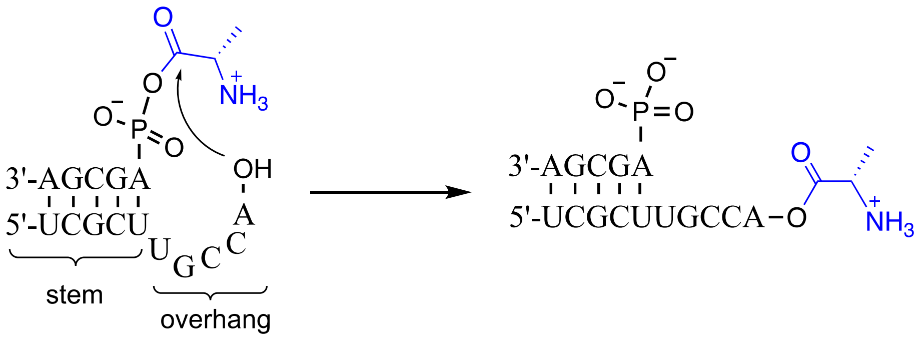

Amino acyl group	Acceptor sequence		pH, Temp.	NaCl (mM)	$MgCl_2$ (mM)	Observed yield	Corrected yield	Mixed anhydride $t_{1/2}$ (h)	Diol ester $t_{1/2}$ (h)
	Stem	Overhang							
L-Ala	UCGCU	UGCCA	6.8, 10 °C	0	0	15 %	27 %	1.2	5.9
			6.8, 10 °C	100	0	15 %	30 %	0.6	6.7
			6.8, 10 °C	1000	0	26 %	60 %	0.1	3.0
			6.8, 10 °C	0	5	27 %	50 %	0.1	3.8
			6.8, 10 °C	100	5	30 %	55 %	0.2	4.1
			6.4, 10 °C	100	5	23 %	44 %	0.3	11
			7.2, 10 °C	100	5	28 %	53 %	0.1	3.4
			6.8, 0 °C	100	5	28 %	56 %	0.3	12
			6.8, 20 °C	100	5	28 %	43 %	0.1	2.7

Table S2. Length and sequence dependence of L-Ala transfer. Conditions: 100 µM of (pAGCGA + L-Ala-pAGCGA), 100 µM of acyl acceptor RNA strand, NaCl 100 mM, $MgCl_2$ 5 mM, 50 mM of HEPES at pH 6.8, 10°C. "-", do not apply, or yields were too low to allow reliable quantification. a) no acceptor RNA was added. N.D., product not detected.

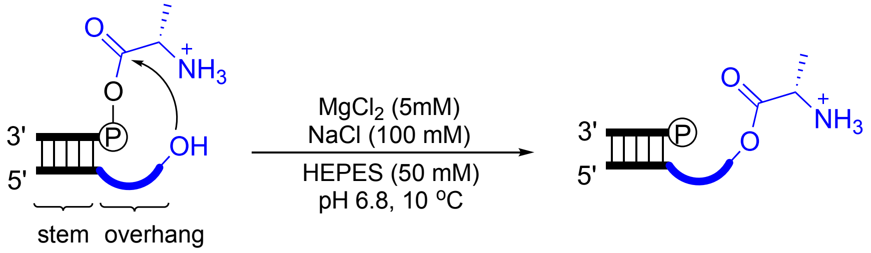

Amino acyl group	Acceptor sequence: Stem	Acceptor sequence: Overhang	Observed yield	Calculated yield	Mixed anhydride $t_{1/2}$ (h)	Diol ester $t_{1/2}$ (h)
L-Ala	UCGCU	**UGCCCA**	22 %	38 %	0.4	8.8
		UGCCA	30 %	55 %	0.2	4.1
		**UCCA**	1 %	2 %	0.3	-
		**ACCA**	N.D.	N.D.	0.6	-
		UGCC**A(2'd)**	4 %	6 %	0.4	-
		UGCC**A(3'd)**	N.D.	N.D.	0.4	-
		U**A**CCA	16 %	30 %	0.4	12
		U**C**CCA	N.D.	N.D.	0.2	-
		U**U**CCA	34 %	57 %	0.5	10
		UGCC**C**	N.D.	N.D.	0.8	-
		UGCC**G**	14 %	25 %	0.3	6.8
		UGCC**U**	N.D.	N.D.	0.5	-
	-[a]	-[a]	-	-	0.9	-

Table S3. Length and sequence dependence of D-Ala transfer. Conditions: 100 µM of (pAGCGA + D-Ala-pAGCGA), 100 µM of UCGCUUGCCA / UCGCUUGCCA, NaCl 100 mM, $MgCl_2$ 5 mM, 50 mM of HEPES at pH 6.8, 10°C. a) 1:1 mixture of the pre-synthesised L-Ala-pAGCGA and D-Ala-pAGCGA was used as acyl donor. b) no acceptor RNA was added. "-", do not apply , or yields were too low to allow reliable quantification. N.D., product not detected.

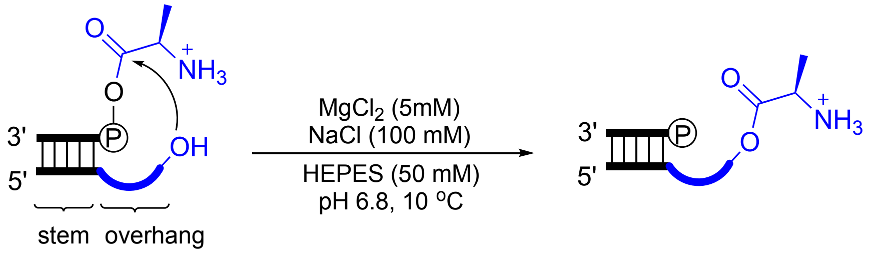

Amino acyl group	Acceptor sequence		Observed yield	Calculated yield	Mixed anhydride $t_{1/2}$ (h)	Diol ester $t_{1/2}$ (h)
	Stem	Overhang				
D-Ala	UCGCU	**UGCCCA**	15%	25%	0.4	9.4
		UGCCA	10%	15%	0.3	2.5
		U**A**CCA	N.D.	N.D.	0.3	--
		U**C**CCA	N.D.	N.D.	0.4	--
		U**U**CCA	4%	6%	0.4	1.4
		UGCC**C**	N.D.	N.D.	0.4	--
		UGCC**G**	8%	12%	0.3	4.1
		UGCC**U**	N.D.	N.D.	0.4	--
	–[b]	–[b]	--	--	0.9	--

Table S4. Length and sequence dependence of Gly transfer. Conditions: 100 µM of (pAGCGA + Gly-pAGCGA), 100 µM of acceptor oligo, NaCl 100 mM, $MgCl_2$ 5 mM, 50 mM of HEPES at pH 6.8, 10°C. a) no acceptor RNA was added. "-", do not apply, or yields were too low to allow reliable quantification. N.D., product not detected.

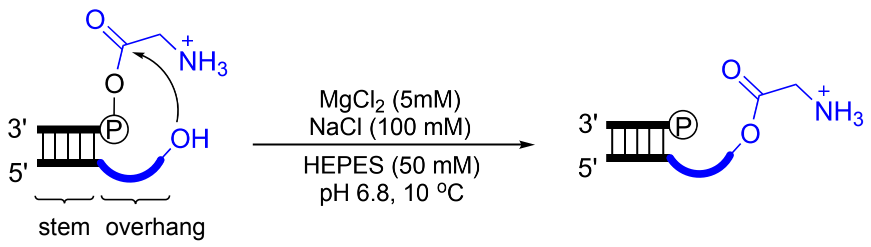

Amino acyl group	Acceptor sequence		Observed yield	Calculated yield	Mixed anhydride $t_{1/2}$ (h)	Diol ester $t_{1/2}$ (h)
	Stem	Overhang				
Gly	UCGCU	**UGCCCA**	17 %	23 %	0.5	15
		UGCCA	9 %	11 %	0.3	20
		**UCCA**	N.D.	N.D.	1.1	-
		**ACCA**	N.D.	N.D.	0.5	-
		U**A**CCA	N.D.	N.D.	0.7	-
		U**C**CCA	N.D.	N.D.	0.8	-
		U**U**CCA	2 %	3 %	0.7	-
		UGCC**C**	N.D.	N.D.	0.8	-
		UGCC**G**	N.D.	N.D.	0.4	-
		UGCC**U**	N.D.	N.D.	0.7	-
	-[a]	-[a]	-	-	0.9	-

Table S5. pH and salt dependence of Ac-Gly transfer. Conditions: 100 µM of (pAGCGA + Ac-Gly-pAGCGA), 100 µM of acyl acceptor RNA, 1 M NaCl, 50 mM of HEPES or MES buffer at pH indicated at 20°C. "-", Do not apply, or yields were too low to allow reliable quantification. a) 50 mM MES buffer. b) 50 mM HEPES buffer. c) The acceptor RNA has no overhang when forming a duplex. d) No acceptor RNA was added.

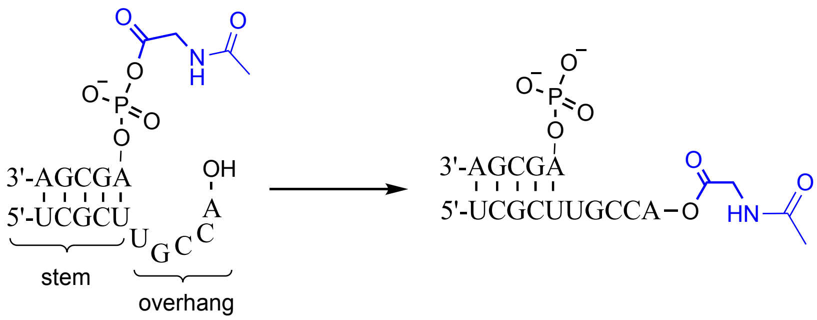

Amino acyl group	Acceptor sequence		pH	NaCl (mM)	$MgCl_2$ (mM)	Observed yield	Calculated yield	Mixed anhydride $t_{1/2}$ (h)	Diol ester $t_{1/2}$ (h)
	Stem	Overhang							
Ac-Gly	UCGCU	UGCCA	6.0[a]	1000	0	8 %	7 %	13.3	-
			7.0[b]	1000	0	17 %	40 %	2.4	27
			7.5[b]	1000	0	19 %	44 %	1.3	15
			8.0[b]	1000	0	23 %	53 %	0.6	8
			7.0[b]	500	0	13 %	30 %	5.5	29
			7.0[b]	0	10	13 %	30 %	2.3	37
	pUCGCU	-[c]	7.0[b]	1000	0	-	-	9.4	-
	-[d]	-[d]	7.0[b]	1000	0	-	-	9.2	-

Table S6. Length and sequence dependence of Ac-Gly transfer. Conditions: 100 µM of (pAGCGA + Ac-Gly-pAGCGA), 100 µM of acyl acceptor RNA, 1 M NaCl, 100 mM of HEPES pH 7.5 at 20°C. "-", do not apply, or yields were too low to allow reliable quantification. a) no acceptor RNA was added.

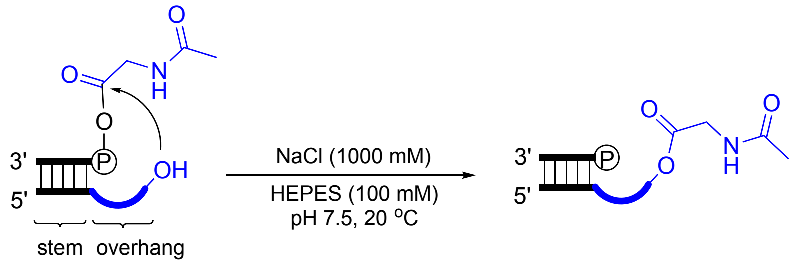

Amino acyl group	Acceptor sequence		Observed yield	Calculated yield	Mixed anhydride $t_{1/2}$ (h)	Diol ester $t_{1/2}$ (h)
	Stem	Overhang				
Ac-Gly	UCGCU	**UGCCCA**	20 %	32 %	1.7	18
		UGCCA	32 %	46 %	0.9	16
		**UCCA**	23 %	37 %	1.4	19
		**ACCA**	4 %	6 %	3.3	-
		UGCC**C**	5 %	7 %	3.9	-
		UGCC**G**	25 %	36 %	1.1	23
		UGCC**U**	5 %	7 %	3.3	-
		U**A**CCA	27 %	36 %	1.7	15
		U**C**CCA	36 %	46 %	1.6	16
		U**U**CCA	21 %	28 %	1.3	20
		UGCC**A(2'd)**	17 %	23 %	3.4	46
		UGCC**A(3'd)**	14 %	19 %	3.2	45
	- [a]	- [a]	-	-	4.2	-

Table S7. Length dependence of Ac-Ala transfer. Conditions: 100 µM of (pAGCGA + Ac-L/D-Ala-pAGCGA), 100 µM of acyl acceptor RNA, 1 M NaCl, 100 mM of HEPES pH 7.5 at 20°C. "-", do not apply, or yields were too low to allow reliable quantification. a) not determined because of overlapping of peaks in HPLC. b) no acceptor RNA was added.

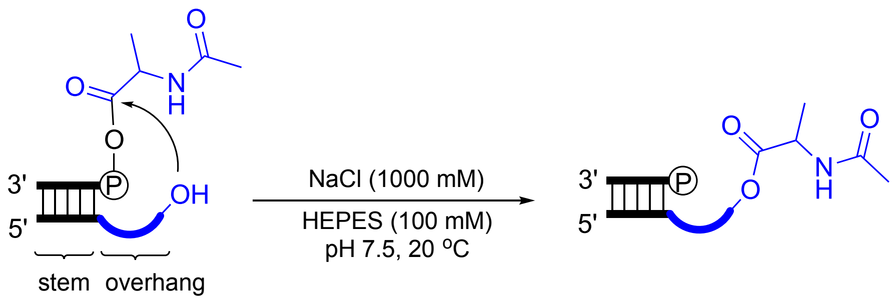

Amino acyl group	Acceptor sequence		Observed yield	Calculated yield	Mixed anhydride $t_{1/2}$ (h)	Diol ester $t_{1/2}$ (h)
	Stem	Overhang				
Ac-Ala	UCGCU	**UGCCCA**	11 %	14 %	1.4	25
		UGCCA	20 %	25 %	1.2	25
		**UCCA**	10 %	13 %	-[a]	22
		**ACCA**	2 %	3 %	1.6	-
	-[b]	-[b]	-	-	1.6	-

Table S8. Sequence dependence and stereoselectivity of Ac-Ala transfer. Conditions: 100 µM of (pAGCGA + Ac-L/D-Ala-pAGCGA), 100 µM of acyl acceptor RNA, 1 M NaCl, 100 mM of HEPES pH 7.5 at 20°C. "-", do not apply, or yields were too low to allow reliable quantification. a) Mixed anhydride was made from starting Ac-L-Ala. b) Mixed anhydride was made from starting Ac-D-Ala. c) 1:1 mixture of the mixed anhydride made from starting Ac-L-Ala and Ac-D-Ala, respectively. N.D., product not detected.

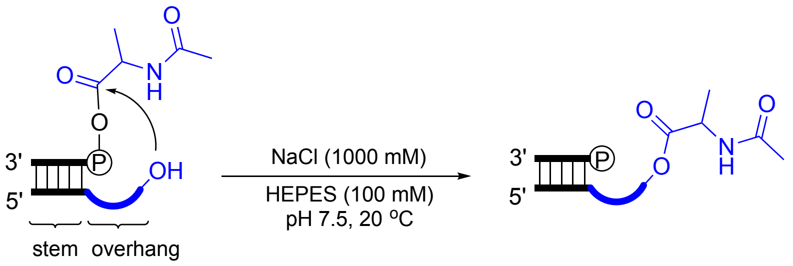

Amino acyl group	Acceptor sequence		Observed yield	Calculated yield	Mixed anhydride $t_{1/2}$ (h)	Diol ester $t_{1/2}$ (h)
	Stem	Overhang				
Ac-Ala	UCGCU	UGCCA	20 %	25 %	1.2	27
		UGCC**C**	N.D.	N.D.	1.7	-
		UGCC**G**	12 %	13 %	1.2	25
		UGCC**U**	N.D.	N.D.	1.8	-
		U**A**CCA	9 %	10 %	1.7	22
		U**C**CCA	8 %	9 %	1.6	27
		U**U**CCA	14 %	16 %	1.3	21
		UGCC**A(2'd)**	3 %	4 %	1.9	-
		UGCC**A(3'd)**	2 %	2 %	1.8	-
Ac-L-Ala[a]		UGCCA	17 %	24 %	0.9	24
Ac-D-Ala[b]		UGCCA	17 %	24 %	1.0	21
Ac-L/D-Ala[c]		UGCCA	17 %	24 %	1.2	20

References:

27. H. Grosjean & E. Westhof, An integrated, structure- and energy-based view of the genetic code. *Nucl. Acids Res.* **2016** *44*, 8020–8040.

28. G. J. Quigley & A. Rich, Structural domains of transfer RNA molecules. *Science* **1976** *194*, 796–804.
